# Mapping Theories, Models, and Frameworks to Evaluate Digital Health Interventions: Scoping Review

**DOI:** 10.2196/51098

**Published:** 2024-02-05

**Authors:** Geneviève Rouleau, Kelly Wu, Karishini Ramamoorthi, Cherish Boxall, Rebecca H Liu, Shelagh Maloney, Jennifer Zelmer, Ted Scott, Darren Larsen, Harindra C Wijeysundera, Daniela Ziegler, Sacha Bhatia, Vanessa Kishimoto, Carolyn Steele Gray, Laura Desveaux

**Affiliations:** 1 Nursing department Université du Québec en Outaouais Saint-Jérôme, QC Canada; 2 Institute for Health System Solutions and Virtual Care Toronto Women’s College Hospital Toronto, ON Canada; 3 Institut du Savoir Montfort Montfort Hospital Ottawa, ON Canada; 4 Southampton Clinical Trials Unit University of Southampton Southampton United Kingdom; 5 Canada Infoway Toronto, ON Canada; 6 Healthcare Excellence Canada Ottawa, ON Canada; 7 School of Nursing Hamilton Health Sciences McMaster University Hamilton, ON Canada; 8 Telus Healthcare Delivery Women's College Hospital Toronto, ON Canada; 9 Women's College Hospital Family Health Team Women's College Hospital Toronto, ON Canada; 10 Schulich Heart Program Sunnybrook Health Sciences Centre Toronto, ON Canada; 11 Centre Hospitalier de l'Université de Montréal Montreal, QC Canada; 12 Ontario Health Toronto, ON Canada; 13 Peter Munk Cardiac Centre University Health Network Toronto, ON Canada; 14 Lunenfeld-Tanenbaum Research Institute Sinai Health Toronto, ON Canada; 15 Institute for Health Policy, Management and Evaluation University of Toronto Toronto, ON Canada; 16 Institute for Better Health Trillium Health Partners Toronto, ON Canada

**Keywords:** digital health interventions, evaluation, implementation, integrated knowledge translation, theories, models, frameworks, scoping review

## Abstract

**Background:**

Digital health interventions (DHIs) are a central focus of health care transformation efforts, yet their uptake in practice continues to fall short of their potential. In order to achieve their desired outcomes and impact, DHIs need to reach their target population and need to be used. Many factors can rapidly intersect between this dynamic of users and interventions. The application of theories, models, and frameworks (TMFs) can facilitate the systematic understanding and explanation of the complex interactions between users, practices, technology, and health system factors that underpin research questions. There remains a gap in our understanding of how TMFs have been applied to guide the evaluation of DHIs with real-world health system operations.

**Objective:**

This study aims to map TMFs used in studies to guide the evaluation of DHIs. The objectives are to (1) describe the TMFs and the constructs they target, (2) identify how TMFs have been prospectively used (ie, their roles) in primary studies to evaluate DHIs, and (3) to reflect on the relevance and utility of our findings for knowledge users.

**Methods:**

This scoping review was conducted in partnership with knowledge users using an integrated knowledge translation approach. We included papers (eg, reports; empirical quantitative, qualitative, and mixed methods studies; conference proceedings; and dissertations) if primary insights resulting from the application of TMFs were presented. Any type of DHI was eligible. Papers published from 2000 and onward were mainly identified from the following databases: MEDLINE (Ovid), CINAHL Complete (EBSCOhost), PsycINFO (Ovid), EBM Reviews (Ovid), and Embase (Ovid).

**Results:**

A total of 156 studies published between 2000 and 2022 were included. A total of 68 distinct TMFs were identified across 85 individual studies. In more than half (85/156, 55%) of the included studies, 1 of following 6 prevailing TMFs were reported: Consolidated Framework for Implementation Research (n=39); the Reach, Effectiveness, Adoption, Implementation, and Maintenance Framework (n=17); the Technology of Acceptance Model (n=16); the Unified Theory on Acceptance and Use of Technology (n=12); the Diffusion of Innovation Theory (n=10); and Normalization Process Theory (n=9). The most common intended roles of the 6 TMFs were to inform data collection (n=86), to inform data analysis (n=69), and to identify key constructs that may serve as barriers and facilitators (n=52).

**Conclusions:**

As TMFs are most often reported to be applied to support data collection and analysis, researchers should consider more clearly synthesizing key insights as practical use cases to both increase the relevance and digestibility of their findings. There is also a need to adapt or develop guidelines for better reporting DHIs and the use of TMFs to guide evaluation. Hence, it would contribute to ensuring ongoing technology transformation efforts are evidence and theory informed rather than anecdotally driven.

## Introduction

### Background

Digital health interventions (DHIs) are a central focus of health care transformation efforts worldwide [1-4], yet their uptake in practice continues to fall short of their potential [5-8]. DHIs are complex interventions with multiple components that fulfill a range of functionalities such as supporting communication, decision-making, documentation and maintenance of patient records, diagnosis, and access to therapies. They target a range of patients, health care providers (HCPs), and health system users and are deployed in a variety of settings (eg, hospital, community, and home) [9] in hopes of delivering on the Quadruple Aim [10,11]. The Quadruple Aim is intended to improve population health, patients’ and caregivers’ experiences, and providers’ experience and to reduce costs. To achieve the desired outcomes, the ideal first step would involve the “determination and optimisation of reach and uptake by the intended population, in the context in which the DHI will be used” [10]. In reality, and at an increasing rate, DHIs are implemented in practice in the paucity of fulsome evidence of their effect; studies being limited to pilot or feasibility ones [12]. This is partly a product of the timelines of traditional research and the rapid pace of technology progress [13]. As a result, evaluations of DHIs may seek to answer various research questions about their effectiveness, associated implementation outcomes [10,14], or both at different stages of the research cycle. For instance, it is appropriate to evaluate the feasibility of DHIs by focusing on implementation outcomes such as reach, adoption, practicability, and acceptability, as well as to determine the impacts of DHIs components on the expected outcomes [10,15]. We refer to evaluation throughout this paper in this broad sense, consistent with our previous work [16], encompassing the systematic assessment of an intervention’s design, implementation, and outcomes that can judge merit, worth, or significance by combining evidence and values [17,18]. The term “evaluation” is then inclusive of various evaluation activities, different types of evaluation (eg, process evaluation, implementation evaluation, as well as impact and outcome evaluation) purposes, and research questions [17,18].

In reality, a variety of other factors influence the successful (or failed) implementation of DHIs [19], including but not limited to funding structure, policy, organizational settings, the complex interactions between users, existing routines and processes, the value proposition, and the technology itself [20]. One way of facilitating the systematic understanding and explanation of the complex interactions between users, practices, technology, and health system factors that underpin research questions [20,21] is to use theories, models, and frameworks (TMFs). There is a wide range of TMFs that have been used in studies of knowledge translation [22,23], and implementation science [24] (examples of more than 40 TMFs are cited). Heinsch et al [24] identified 36 theories for informing and explaining eHealth implementation, and Greenhalgh et al [20] identified 28 technology-specific implementation frameworks. In their systematic review, Bashi et al [12] identified 11 evaluation frameworks applied in the management of chronic diseases. The authors of those works use either the terms “implementation frameworks” or “evaluation frameworks.” Despite this body of knowledge on TMFs, there remains a gap in our understanding of how TMFs have been and could be applied to guide the reported prospective evaluation of DHIs with real-world health system operations.

### Objectives

The aim of this work was to map the TMFs used in studies to evaluate DHIs. Specifically, our objectives were to (1) describe the TMFs and the constructs they target, (2) identify how TMFs have been used in primary studies (hereafter referred to as the roles of the TMFs), and (3) reflect on the relevance and utility of our findings for knowledge user partners as a post hoc objective. A scoping review was the suited knowledge synthesis approach to map in a comprehensive way the current state of evidence in this given area and to cover a breadth of the literature [25,26].

## Methods

### Protocol and Registration

We conducted a scoping review, informed by the Joanna Briggs Institute methodology [27]. The protocol has been published previously [28]. We used the PRISMA-ScR (Preferred Reporting Items for Systematic Reviews and Meta-Analyses Extension for Scoping Reviews) checklist to inform reporting [29].

### Integrated Knowledge Translation Approach

**Figure 1:**
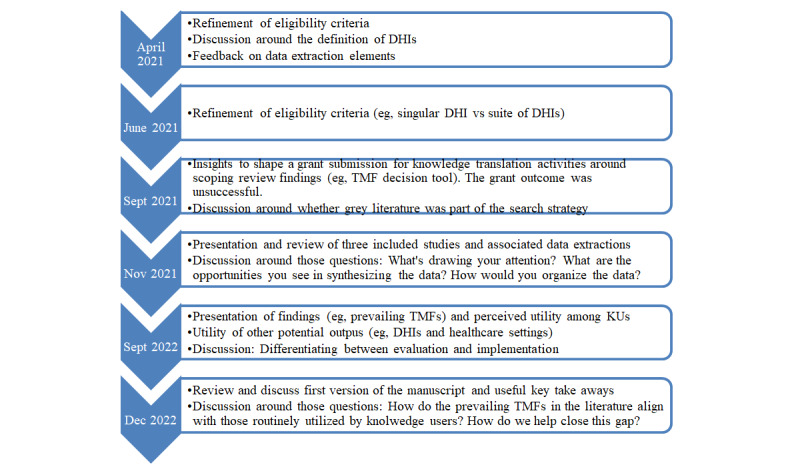
Timeline and content of meetings with knowledge users. DHIs: digital health interventions; TMF: theory, model, and framework.

This work was conducted in partnership with knowledge users using an integrated knowledge translation strategy [30,31]. The aim of the strategy was to inform the objectives and approach, develop a shared understanding of the findings, and work with knowledge users to understand how the resulting knowledge could be synthesized to support its application in practice. The knowledge users were identified by the leadership of the Centre for Digital Health Evaluation and, especially, by its director and scientific lead at the time of the initiation of the study. Only 1 person out of 7 declined the email invitation. The advisory panel included senior leaders (DL, HCW, SM, JZ, TS, and SB), policy makers (JZ and SB), a researcher (CSG), clinicians (DL, HCW, and SB), and a DHI developer (DL) who are involved in health decision making regarding the evaluation of DHIs. The knowledge users advisory panel provided input to the protocol of the scoping review [28], supported the refinement of the eligibility criteria of included papers, identified relevant data abstraction elements to prioritize, and assisted in the interpretation of findings. Furthermore, as a mechanism of reflexivity [32], they shared their vision and experience about using TMFs in their respective context of work. Consequently, it helped to inform the results. Details regarding knowledge users’ profiles, area of expertise, and their application of TMFs are available in Multimedia Appendix 1. Two newsletters were sent out to inform knowledge users about progress updates and upcoming activities (eg, titles and abstracts screening and data extraction). Knowledge users participated in 6 meetings over Zoom (Zoom Video Communications) between March 2021 and December 2022, with each meeting lasting 60 minutes. Figure 1 illustrates the timeline of the meetings, the topics covered, and some examples of questions discussed.

### Critical Appraisal of Individual Sources of Evidence

Given that the purpose of our scoping review was to provide a broad overview of the TMFs used in relation to DHIs and not to recommend evidence to guide clinical practice, the methodological quality of included studies was not assessed; this was prespecified in the protocol [28] and was still in line with methodological guidance [27].

### Eligibility Criteria

To enhance translation into practice, the eligibility criteria focused on papers in which authors reported having used TMFs prospectively to guide DHI evaluation. Retrospective application of TMFs was excluded. We were interested in understanding how TMFs can be prospectively used when undertaking a theoretical-based evaluation of DHIs.

### Type of Interventions

DHIs are defined as the use of various digital technologies (eg, eHealth, telemedicine, patient remote monitoring, smartphone apps, patient sensors, and artificial intelligence) to improve health care delivery [33]. We included papers that reported on a single DHI and excluded those in which a collection or suite of DHIs was presented (eg, variety of electronic medical records) and those in which DHIs were described broadly (eg, eHealth systems having no identified features or components). This exclusion criterion was mainly decided to narrow down the number of included studies in order to increase the feasibility of performing the knowledge synthesis with the constrained resources we had.

### Type of Literature

We included peer-reviewed quantitative, qualitative, and mixed methods empirical studies reporting on the application of TMFs to prospectively guide the evaluation of DHIs in health care. Published gray literature, including conference abstracts or proceedings, dissertations, reports, and white papers, have been included if primary insights resulting from the application of TMFs were presented. Reviews, study protocols, commentaries, and letters to the editor were excluded as they had no primary data.

### Type of Participants

No limitations were placed on the user population as long as the evaluation of DHIs in a health care context was described.

### Type of TMFs

We included studies that pursued different research questions in which TMFs were applied to guide the evaluation of DHIs. We excluded papers that described the theoretical underpinnings and the overall process of intervention development. We made this decision at the early stage of the scoping review given the abundance of papers focusing on the intervention development; and the limited resources we had in completing the knowledge synthesis.

### Information Sources and Search Strategy

The following electronic databases were searched: MEDLINE (Ovid), CINAHL Complete (EBSCOhost), PsycINFO (Ovid), EBM Reviews (Ovid), and Embase (Ovid). A complementary search of Google Scholar was conducted to identify relevant studies. Search strategies were designed by a librarian (DZ) and were peer-reviewed by another senior information specialist prior to execution using the PRESS Checklist [34]. We imposed no language restrictions and the search extended to studies published in 2000 and onward. The search was initially run on March 10, 2021, and then updated on March 16, 2022. The full electronic search strategy is presented in Multimedia Appendix 2. Due to resource constraints and delays introduced by the pandemic, we did not apply supplementary search strategies as planned in the protocol (ie, checking the reference lists of included studies, and conducting a forward citation search).

### Eligibility Screening Process

Citations obtained from the literature were stored in Endnote (Version X9; Clarivate) [35] and then uploaded to Covidence (Veritas Health Information) [36], a web-based collaboration software platform that streamlines the production of systematic and other literature reviews. This software allows multiple reviewers to participate in various stages of the review (ie, screening titles, abstracts, and full texts and identifying discrepancies). We applied a 2-step process for identifying relevant citations. At stage 1, titles and abstracts were independently assessed by 5 reviewers (RHL, GR, KR, CB, and VK). Studies with abstracts fulfilling the criteria were passed to level 2 full-text screening. At this stage, each full text was reviewed by 1 person (a total of 7 team members: RHL, GR, KR, VK, SM, CB, and KW). All reviewers flagged full texts they were unsure about, and these were validated by a second reviewer. A pilot test of the screening strategy was completed using a random sample of 10% of citations and full-text papers prior to full implementation, with the expressed purpose of assessing agreement between reviewers at each level (interrater reliability ≥80% was considered adequate). When agreement was not reached, a third reviewer (RHL and GR) mediated any disagreements.

### Data Extraction Process and Data Items

Studies fulfilling the eligibility criteria were extracted in Microsoft Excel. The following study characteristics were collected: reference, country of origin of the first author, study design, and DHI user. We also extracted data specific to the TMFs, including name; constructs; variables or mechanisms; and roles of framework in the study, that is, how it has been applied in research. We pilot-tested the data extraction form by extracting data from the same study as a team and iteratively adapted the form. We had biweekly working meetings to discuss the process, highlight challenges, and identify strategies to mitigate those challenges.

### Data Synthesis

We undertook a descriptive quantitative and qualitative data analysis. First, we did simple frequency counts in line with Joanna Briggs Institute guidance [27] of interventions, frameworks, and roles of the TMFs. Descriptive qualitative content analysis involved categorizing 2 sets of data: DHI users and roles of frameworks. We used the World Health Organization taxonomy [37] to categorize the DHIs according to their primary targeted users: (1) clients (potential or actual users of health services, including caregivers), (2) HCPs (deliverers of health services), (3) health system managers (“involved in the administration and oversight of public health systems”), and (4) data services (crosscutting functionality supporting various activities focusing on data collection, management, use, and exchange). A single DHI could be categorized in more than 1 domain (eg, it can target both clients and HCPs). We coded TMF roles according to the use classifications outlined by Birken et al [38] (Textbox 1).

The knowledge users advisory panel informed the synthesis of findings, including the level of detail abstracted from included papers and the approach to DHI classification.

**Roles of theories, models, and frameworks according to the classifications by Birken et al .**

- To identify key constructs that may serve as barriers and facilitators
- To inform data collection
- To guide implementation planning
- To enhance conceptual clarity
- To specify the process of implementation
- To frame an evaluation
- To inform data analysis
- To guide the selection of implementation strategies
- To specify outcomes
- To clarify terminology
- To convey the larger context of the study
- To specify hypothesized relationships between the constructs

## Results

### Search Results

**Figure 2:**
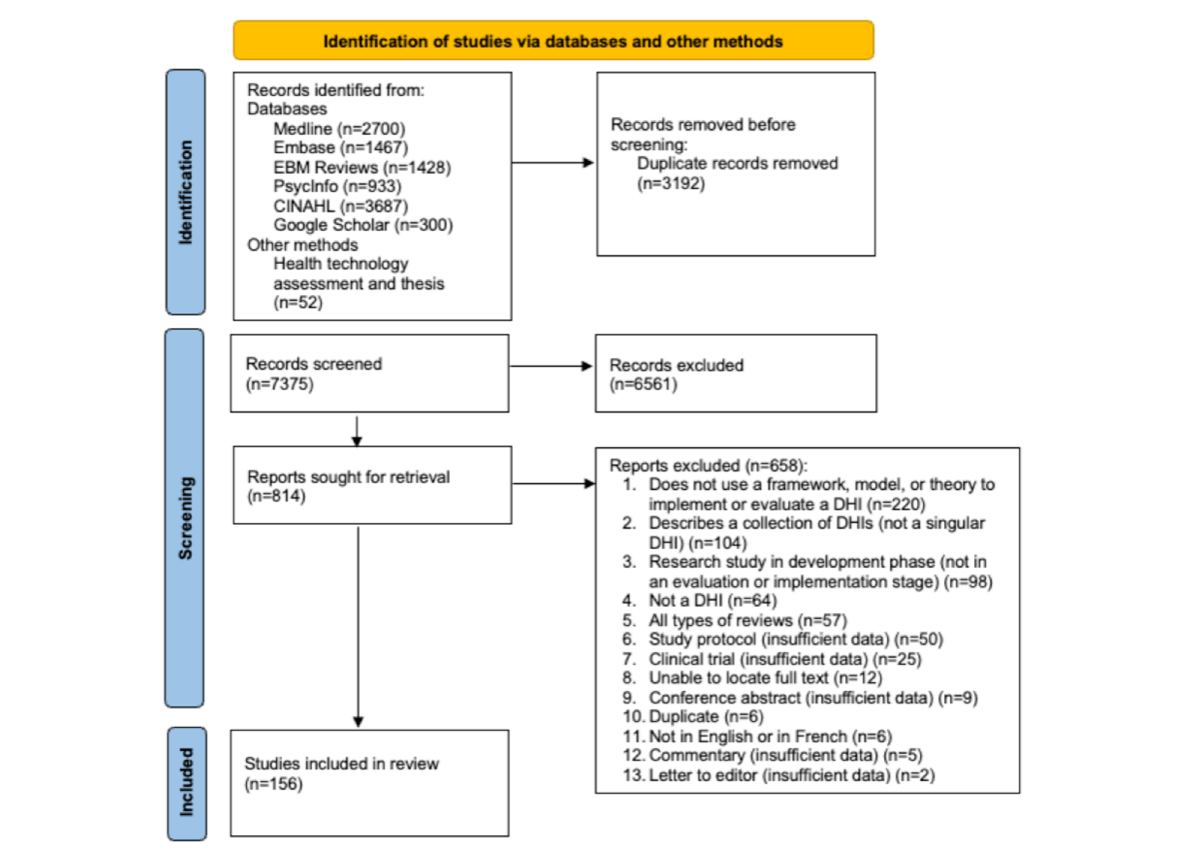
The PRISMA (preferred reporting items for systematic reviews and meta-analyses) study flow diagram. DHI: digital health intervention.

A total of 10,567 titles or abstracts were identified from the 5 databases, Google Scholar, and other methods, from which 3192 were removed in EndNote by the librarian (DZ). After removing duplicate references, 7375 titles or abstracts were assessed for eligibility. Of these, 6561 papers were excluded based on title and abstract screening and application of the eligibility criteria previously outlined. A total of 814 full-text papers were sought and screened, and 658 were excluded. The list of excluded studies is presented in Multimedia Appendix 3. Between 2000 and 2022, a total of 156 published papers met the eligibility criteria. The list of these included papers is presented in Multimedia Appendix 4. The PRISMA (Preferred Reporting Items for Systematic Reviews and Meta-Analyses) study flow diagram [39] is illustrated in Figure 2 to show the overall process of review selection.

### Study Characteristics

The majority of papers were published by researchers in the United States (n=64), Canada (n=19), the United Kingdom (n=14), the Netherlands (n=12), and Australia (n=7). Study designs were largely qualitative (n=63) and mixed methods (n=62), with a smaller number of quantitative studies (n=31). Most DHIs targeted either HCPs (n=67) or clients (n=63), with a few targeting health system managers (n=8), data services (n=3), and a combination of users (n=15).

### Identification of Theories and the Most Reported TMFs

**Figure 3:**
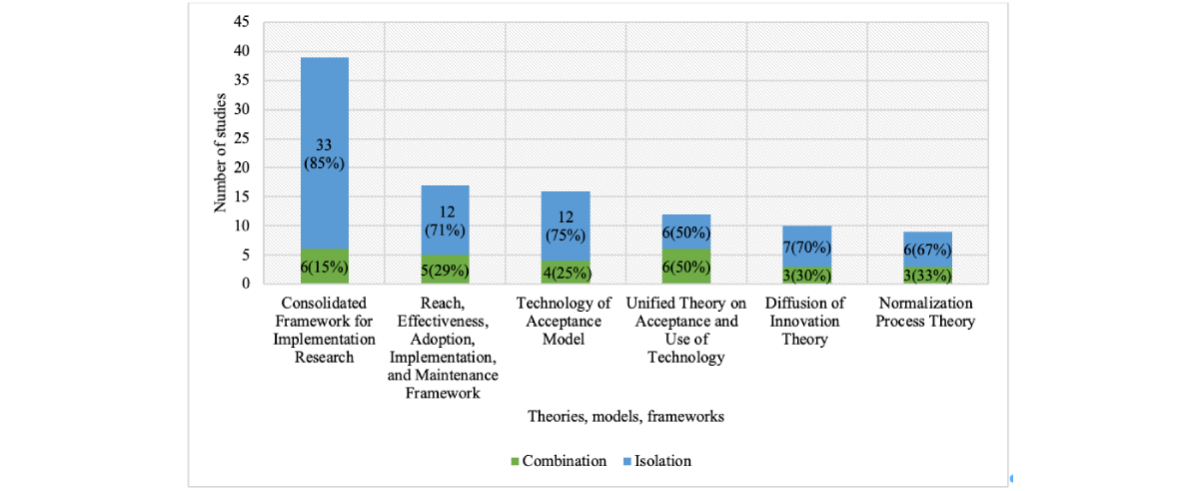
Nature of application of theories, models, and frameworks across included studies.

In total, 68 distinct TMFs were identified (see Multimedia Appendix 5) across 85 individual studies. More than half (85/156, 55%) of included studies used 1 of 6 TMFs, which included the CFIR (Consolidated Framework for Implementation Research; 39 studies), the RE-AIM (Reach, Effectiveness, Adoption, Implementation, and Maintenance Framework; 17 studies), the TAM (Technology of Acceptance Model; 16 studies), the UTAUT (Unified Theory on Acceptance and Use of Technology; 12 studies), the DOI (Diffusion of Innovation Theory; 10 studies), and the NPT (Normalization Process Theory; 9 studies). It should be noted that the number of studies across the 6 TMFs is 103; however, because TMFs are used in combination with other theoretical approaches (Figure 3), this number represents 85 individual studies. UTAUT is the theory most frequently used in combination with other TMFs. A descriptive table of those studies that includes references, DHI user, study type, TMF used in combination, and roles of TMFs is presented in Multimedia Appendix 6. Our results focus on synthesizing insights across the 6 prevailing TMFs, being reported in 9 studies or more, allowing us to synthesize their application across different studies and contexts of evaluation. The constructs of the prevailing TMFs are described and summarized in the Multimedia Appendix 7.

The most common intended roles of the 6 TMFs were to inform data collection (n=86), to inform data analysis (n=69), to identify key constructs that may serve as barriers and facilitators (n=52), to organize and report the study findings (n=47), and to frame an evaluation (n=18; see Table 1). TMFs were applied to pursue various roles, that is, they served multiple purposes. The average number of distinct roles per TMFs is as follows: RE-AIM (n=3), CFIR (n=2.9), NPT (n=2.56), DOI (n=1.9), UTAUT (n=1.75), and TAM (n=1.69).

**Table 1:** Intended roles of TMFs^a^ as specified across the studies.

Roles of TMFs	6 prevailing TMFs; number of roles identified across all studies					
	CFIR^b^	RE-AIM^c^	TAM^d^	UTAUT^e^	DOI^f^	NPT^g^
To identify barriers and facilitators	31	8	3	4	4	2
To inform data collection	25	26	14	9	3	8
To inform data analysis	32	18	7	3	3	6
To organize and report study findings	15	16	5	3	4	4
To guide implementation planning	7	2	0	0	0	1
To guide the selection of implementation strategies	3	1	0	1	1	1
To frame an evaluation	3	9	1	1	2	2
To clarify terminology	0	0	0	0	1	0
To specify the process of implementation	1	0	0	0	0	0
To specify hypothesized relationships between constructs	0	0	0	0	1	0

^a^TMF: theory, model, and framework.

^b^CFIR: Consolidated Framework for Implementation Research.

^c^RE-AIM: Reach, Effectiveness, Adoption, Implementation, and Maintenance Framework.

^d^TAM: Technology of Acceptance Model.

^e^UTAUT: Unified Theory on Acceptance and Use of Technology.

^f^DOI: Diffusion of Innovation Theory.

^g^NPT: Normalization Process Theory.

### DHIs and Intended Users Associated With Top 6 Frameworks

DHIs targeting clients and HCPs are the most frequently reported (see Table 2). RE-AIM and DOI were used for DHIs designed for clients, while CFIR, TAM, UTAUT, and NPT have been used primarily with DHIs involving HCPs. DHIs targeting clients included patient portals [40,41], web-based self-management interventions [42,43], and mobile health diet apps [44]. DHIs targeting HCPs included (but are not limited to) mobile apps targeting patients’ smoking cessation [45] and medication adherence counseling [46,47], telemedicine and telehealth [48,49], cancer prevention decision support tools [50], e-consultation between primary care providers and specialty care expertise [51,52], and e-learning for dementia caregiver education [53]. Two studies targeted health system managers and an information system for case-based surveillance [54] and a patient-reported outcome data collection system [55]. Two studies focused on data services including big data analytics [56], the former being reported in combination with clients, and an electronic patient falls reporting system [57].

**Table 2:** Target users of the DHI^a^ across included studies.

Framework	Clients, n	HCPs^b^, n	Health system managers, n	Data services, n	Combination, n
CFIR^c^	13	18	2	0	5
RE-AIM^d^	9	3	0	0	5
TAM^e^	3	9	2	1	1
UTAUT^f^	5	7	0	0	0
NPT^g^	3	5	0	0	1
DOI^h^	5	3	0	0	2

^a^DHI: digital health intervention.

^b^HCPs: health care providers.

^c^CFIR: Consolidated Framework for Implementation Research.

^d^RE-AIM: Reach, Effectiveness, Adoption, Implementation, and Maintenance Framework.

^e^TAM: Technology of Acceptance Model.

^f^UTAUT: Unified Theory on Acceptance and Use of Technology.

^g^NPT: Normalization Process Theory.

^h^DOI: Diffusion of Innovation Theory.

### Current Gaps Between Prevailing TMFs Used in Research and in Practice by Knowledge Users

The knowledge users reflected that most of the prevailing TMFs identified in this scoping review were not familiar to them. They have used different TMFs in practice (see Multimedia Appendix 1) that reflect their interest in capturing the process of implementation and for outcome-driven evaluation approaches that would help them understand whether DHIs work or not. One first example is the NPT, applied to implement and evaluate the effectiveness of the electronic patient-reported outcome (ePRO) mobile app and portal system. ePRO was designed to enable goal-oriented care delivery in interprofessional primary care practices [58]. In this study, many types of outcomes were of interest to produce early evidence of effectiveness (or ineffectiveness) of the ePRO and its mechanisms of action: the context (eg, sociodemographic data and barriers of adopting ePRO), process (eg, usability), and outcome measures (eg, patients’ quality of life, provider-level effectiveness in delivering care to patients with chronic illness). The Quadruple Aim [59] was also used by 5 knowledge users to evaluate the impact of DHIs on health system performance with outcomes such as equitable access, cost reduction, patient-provider relationships, providers’ burnout, and work-life balance [11]. The Benefits Evaluation Framework [60] was also well-known and used by knowledge users. Similarly to TAM and UTAUT, it aims to describe factors influencing eHealth success (eg, system quality, information quality, and user satisfaction), with the addition of the resulting impacts (or outcomes) of DHIs in terms of care quality (eg, effectiveness and health outcomes), access services, and productivity (eg, efficiency).

## Discussion

### Principal Findings

While a wide range of TMFs (n=68) have been used to guide the evaluation of DHIs, 6 main TMFs are used consistently by researchers. These TMFs were used in a variety of roles and were broadly applied across types of DHI and target user groups, demonstrating their flexibility in academic practice. These 6 TMFs were not commonly used by nor familiar to many of the knowledge users, highlighting the disconnect between academic and health system practice. Our discussion presents the 3 key insights from these conversations in relation to our results: specifically, how the application of prevailing TMFs in the literature could be used in health system decision-making, how to bridge the persistent gap between academic knowledge and health system practice, and lessons learned about how future work might bridge this gap.

### Insights From the Application of Prevailing TMFs

The findings allowed us to identify a higher number of TMFs (n=68) than those reported previously by Heinsch et al [24] (n=36) and Greenhalgh et al [20] (n=28). Our findings corroborate the ones in Heinsh et al [24], in which 5 of our prevailing TMFs (except RE-AIM) have been identified. Furthermore, at least 3 TMFs (TAM, DOI, and NPT) identified in our review were cited as a groundwork for technology implementation frameworks as identified by Greenhalgh et al [20]. While this highlights the variability of TMFs used in the evaluation process of DHIs, they are most often used to inform data collection and data analysis, aligning with the findings of Birken et al [38].

While different types of outcomes were of interest to knowledge users deriving from their use of TMFs (see Multimedia Appendix 1), that is, service (eg, efficiency or cost, effectiveness, and access to care), client (eg, patients’ quality of life), and implementation (eg, adoption and sustainability), as aligned with the literature [15], only RE-AIM included an explicit effectiveness outcome domain. Recently, CFIR has been extended to include implementation outcomes and innovation (ie, intervention) outcomes as part of CFIR 2.0 [61,62]. CFIR 2.0 outcomes are inclusive of both purchase and operating costs (the innovation cost) [61]—an element that is central to decision-making within resource-constrained systems [63]. As an example, the perceived advantage of a mobile app from the perspective of HCPs (an implementation determinant) may impact their uptake and referral rate to their patients (an implementation outcome). This is distinct from patient motivation to use the app (an innovation determinant) which will impact weight loss (an innovation outcome). These distinct categories were included to focus attention “squarely on the way that context shapes intermediate results and conditions, such as user acceptance, which in turn influence classic measures of an intervention’s ultimate aims or outcomes” [64]. This highlights the need to consider a chain of short-term proxy outcomes (eg, acceptability of DHIs and adaptation to novel contexts), including the attributes of context [65], if we want to capture the likely benefit of the DHIs [10]. This would help to address the disconnect between (less) attention paid to context in comparison with effectiveness outcomes [10,65].

### Bridging the Gap Between Academic and Health System Practice

Our work echoes the opportunity for researchers to better understand the realities of health care practice and operations [66]. This can be achieved by understanding the context in which knowledge users operate, their values and professional experience from the early beginning of the project [67,68], and assessing the usefulness of TMFs in supporting their routine decision-making. Relatedly, there is an opportunity for researchers to support knowledge users in understanding how to leverage insights from the literature to better achieve their desired outcomes. Greenhalgh et al [20] observed a tendency across DHI implementations “to assume the issues to be addressed were simple or complicated (hence knowable, predictable, and controllable) rather than complex (that is, inherently not knowable or predictable but dynamic and emergent).” Explicitly highlighting how TMFs can mitigate the inherent challenges that knowledge users face in evaluating interventions may help to address this gap.

Our knowledge user panel recommended presenting the TMFs as practical use cases to illustrate their real-world application potential—specifically in nonacademic settings, where implementation and evaluation activities are part of routine operations. The knowledge users shared an interest in TMFs that could support scaling up DHIs and understanding their effectiveness and impact on patient outcomes. Through our discussion with the advisory panel, the CFIR and RE-AIM frameworks were identified as aligning with the dual purpose of guiding the implementation effectiveness (CFIR), as well as scalability and sustainability (RE-AIM) [69]. The use cases were constructed to highlight the utility of the TMFs as well as to demonstrate how and to what end they have been used in research (Multimedia Appendices 8 and 9).

### Lessons Learned and Limitations

A natural evolution of this work would be to provide knowledge users with an easy-to-use tool to select a TMF that aligns with their operational needs and local context. The Theory, Model, and Framework Comparison and Selection Tool [70] can help scientists and practitioners select the most appropriate TMF to meet their needs and realize the potential that a given DHI may, or may not, bring in its intended context. Users are directed to a web tool and repository of TMFs [71] which includes a tutorial for novice users and guidance on how to address their research or practice questions.

Despite the desire among our team to classify DHIs according to their primary function (eg, to communicate with clients and to transmit information), we were constrained by the variability in how DHI-related information was reported. A standardized reporting structure inclusive of DHI function, setting, target users, and intended outcomes would help to facilitate learning across systems and studies as health care becomes increasingly technology enabled. Guideline for reporting evidence-based practice educational interventions and teaching [72] and the Template for Intervention Description and Replication checklist [73] are both used to better report interventions. However, adapting those guidelines to the specificities of DHIs would be valuable in future work. In addition, Krick et al [74] developed a comprehensive digital nursing technology outcome framework that allows the identification of effective outcomes. This outcome framework can be a good starting from which other types of outcomes (such as implementation) can be added. This would potentially address the desire of knowledge users to understand effectiveness outcomes at a categorical level (eg, the effectiveness of DHIs by functional category or setting), which we were unable to achieve due to the heterogeneity of outcomes and terminology used to describe DHIs.

While this work engaged knowledge users from the study conception, our search strategy did not capture the Benefits Evaluation Framework—the primary framework they used in practice despite its application in more than 50 organizationally-led evaluations [75]. This limited our ability to systematically compare TMFs routinely used in the academic literature with those routinely used in practice, which is likely to provide further insights into how knowledge users collect, synthesize, report, and digest evaluation insights. Future integrated knowledge translation projects would benefit from investing time upfront to better understand how knowledge users and team members approach their work and which resources and tools they rely on to ensure research is better positioned to address persistent gaps between academic knowledge to operational practice. Another limitation in the process is that we used the information as reported by the authors to classify the roles of TMFs. We did not interpret the various roles, such as “to convey the larger context of the study” or “to frame an evaluation.” Simply put, if authors did not clearly report their intended purposes for using TMFs, we did not extract the information. Hence, we did not explore to what extent the claimed theory was used. Birken et al [38] highlight that providing guidance for theory selection may encourage implementation scientists to use theories in a meaningful way and discourage superficial use and misuse. Our findings pointed out that the prevailing TMFs were used in combination with other TMFs: adding to the challenges of aligning and using meaningfully the use of multiple TMFs. Reporting guidelines for the use of TMFs to guide evaluation would be an avenue for future research.

### Conclusions

The findings of this scoping review illustrate the range of TMFs applied to support the evaluation of a breadth of DHIs. As TMFs are most often applied to support data collection and analysis, researchers should consider more clearly synthesizing key insights as practical use cases to both increase the relevance and digestibility of their findings. The opportunity to develop a standardized reporting structure inclusive of DHI function, setting, target users, and intended outcomes is quickly becoming a crucial need to ensure ongoing technology transformation efforts are evidence informed rather than anecdotally driven. Finally, guidance on how to effectively report the use of TMFs to guide evaluation would also be needed.

## Appendix

Multimedia Appendix 1 Knowledge users profiles and their use of theories, models, and frameworks.

Multimedia Appendix 2 Full search strategy.

Multimedia Appendix 3 List of excluded papers.

Multimedia Appendix 4 List of included studies.

Multimedia Appendix 5 List of all theories, models and frameworks.

Multimedia Appendix 6 Descriptive table most prevailing theories, models and frameworks.

Multimedia Appendix 7 Constructs of prevailing theories, models, and frameworks.

Multimedia Appendix 8 Use cases of the Consolidated Framework for Implementation Research.

Multimedia Appendix 9 Use cases of the Reach, Effectiveness, Adoption, Implementation, and Maintenance Framework.

Multimedia Appendix 10 PRISMA-ScR checklist.

## Abbreviations

CFIR: Consolidated Framework for Implementation Research
DHI: digital health intervention
DOI: Diffusion of Innovation Theory
ePRO: electronic patient-reported outcome
HCP: health care provider
NPT: Normalization Process Theory
PRISMA: Preferred Reporting Items for Systematic Reviews and Meta-Analyses
PRISMA-ScR: Preferred Reporting Items for Systematic Reviews and Meta-Analyses Extension for Scoping Reviews
RE-AIM: Reach, Effectiveness, Adoption, Implementation, and Maintenance Framework
TAM: Technology of Acceptance Model
TMF: theory, model, and framework
UTAUT: Unified Theory on Acceptance and Use of Technology
