# Supplementary material for: Mapping Theories, Models, and Frameworks to Evaluate Digital Health Interventions: Scoping Review
Source: J Med Internet Res. 2024 Feb 5;26:e51098. doi: 10.2196/51098 (PMC10877497; doi:10.2196/51098)
Supplement: Multimedia Appendix 1 [file jmir_v26i1e51098_app1.docx]

**Multimedia Appendix 1.** Knowledge users’ profiles and use cases of their application of theories, models, and frameworks.

| Knowledge users initials, credentials, geographic location, and roles | Affiliations | Experience and area of expertise | Use cases – How they approach digital health intervention evaluation |
| --- | --- | --- | --- |
| CSG  (MA, PhD)  Toronto, Ontario, Canada  Independent researcher | Lunenfeld-Tanenbaum Research Institute, Sinai Health | As Canada Research Chair in Implementing Digital Health Innovation, Dr. CSG is studying the impact of new digital health technologies and the factors that drive their adoption. She and her research team are trying to better understand the factors that influence technology implementation. They are also creating tools and frameworks to inform implementation and advancing innovative research methods to study these complex systems. By partnering with patients, communities and system leaders, they will ensure their research leads to the knowledge and tools we need to address pressing health system challenges. | She is familiar with Normalization Process Theory (NPT) because she used it in a study aimed to implement and evaluate the effectiveness of the electronic Patient-Reported Outcome (ePRO) mobile app and portal system [1]. NPT was useful in this study to support directed analysis for the purposes of this evaluation, and to understand implementation mechanisms. |
| DL  (MD, CCFP, MPLc)  Toronto, Ontario, Canada  Clinician, senior leader & technology developer | Women’s College Hospital; University of Toronto; TELUS Healthcare Delivery | Family physician and educator in Department of Family and Community Medicine, Women’s College Hospital, University of Toronto; Senior Fellow, Women’s College Hospital Institute for Health System Solutions  and Virtual Care (WIHV); Chief Medical Advisor, TELUS Healthcare Delivery (national). Former Senior Advisor, Canadian Healthcare, Accenture. Former Chief Medical Officer of OntarioMD, a leader in electronic medical records and digital health tools. Focuses on how data and technology can be used as a driver for change and improvement in health care. Plays key roles in many provincial and national health IT, quality and leadership initiatives from more than three decades of experience practising medicine and as a health technology leader. | In the application of digital health interventions in practice in both the clinical and public policy domains in my work, there has a large emphasis on Benefits Evaluation. This is because there is a direct application to the work before or after implementation, process timelines are short and evaluation changes can be implemented immediately. They are also rather uncomplicated in their design and outcome measures, so they answer moderately complex questions well. They provide concrete actionable clarity. They are also relatively cheap to implement and do not need deep skill sets. These are favoured by consultancies.  Quadruple Aim [2] is frequently cited in evaluative work in clinical and public policy domains as well, but direct evaluation under the four quadrants is rarely done. Results are frequently pointed and may be very difficult to change (i.e: physician and nursing burnout nearly always rises to the top in the fourth quadrant). Still, recognizing the framework and contemplating it alongside other tools is illuminating. |
| HCW  (MD, PhD, FRCPC FCCS, FAHA)  Toronto, Ontario, Canada  Clinician & senior leader | Sunnybrook Health Sciences Center  Canadian Agency for Drugs and Technologies in Health (CADTH)  Ontario Health-Corhealth | Provincial Cardiac Lead, Ontario Health-Corhealth (2022-present)  Vice-president of medical devices and clinical intervention (CADTH 2017-2020). Interventional cardiologist and clinician scientist with expertise in health technology assessment. Chief, Schulich Heart Program & Division Head, Cardiology, Sunnybrook Health Sciences Centre.  Brings his expertise knowledge translation and in evaluating digital health interventions. | Not reported |
| SM  (BSc Honours, CPHIMS-CA^a^)  Toronto, Ontario, Canada  Senior lead | Canada Health Infoway (2004-2023) | Executive Vice President, Digital Health Engagement and Marketing. Manages key stakeholder relationships at Canada Health Infoway. Recipient of Digital Health Canada's Inaugural Women Leaders in Digital Health Award in 2017. Expertise in digital health engagement and marketing strategies and a great asset in creating connections and networks. | As a strategic investor of digital health interventions that are implemented in provinces and territories, we mandate that an evaluation framework be used but we don’t dictate which one. The Quadruple/Quintuple aim is often used as is the Benefits Evaluation Framework.  Baseline system use and client satisfaction surveys are collected at the outset and data is collected at various stages of the intervention as well as post-study to assess impact in areas such as quality, efficiency and effectiveness. |
| JZ  (MA, PhD)  Ottawa, Ontario, Canada  Policymaker & senior leader | Healthcare Excellence Canada | President and CEO. Long-standing commitment to improving healthcare quality and safety, as well as expertise in spreading and scaling innovations, including digital health innovations, that deliver better outcomes. | She has had the opportunity to use a variety of implementation and evaluation frameworks over time, including Consolidated Framework for Implementation Research and Technology for Acceptance Model, as well as the Benefits Evaluation Framework [3]. Currently, she is more likely to consider digital health interventions as part of a broader practice or system change, rather than as stand-alone interventions. She therefore chooses to use frameworks (eg., Quadruple/Quintuple Aim [4,5]) that reflect this broader scope. |
| TS  (PhD, MAppSC, CPHIMS-CA)  Toronto, Ontario, Canada  Senior leader | Hamilton Health Sciences, School of Nursing, McMaster University | Chief Innovation Officer.  Hamilton Health Sciences Innovation Function, led by Dr. TS, aims to create new models of care that empower both patients and healthcare providers. The vision addresses the need for the healthcare industry to evolve the relationships between patients, and healthcare providers using digital solutions, artificial intelligence and data science. | When we evaluate our digital health interventions, our focus is on the Quadruple Aim.  Whether it is a complex digitally enabled change in the model of care, such as our surgical transitions program, or a relatively simple intervention like the Voyce translation tool, we approach evaluation through the lens of the Quadruple Aim.  This means we typically acquire qualitative data from patients and providers along with quantitative data describing healthcare outcomes and costs. Altogether, these data points define a shift towards the Quadruple Aim and determine the sustainability and value of new digitally enabled initiatives.  The Quadruple Aim measures are developed prior to project initiation, at the completion of the project and ongoing after procurement as well. This is intended to support the overall sustainability of the program which typically evolves or can branch into adjacent areas such as oncology for the example noted in the paragraph above. |
| SB  (MD, MBA)  Policymaker, senior leader, & clinician  Toronto, Ontario, Canada | Ontario Health | Population Health and Values Based Health Systems Executive. Supports in leading Ontario Health’s work in the implementation of Ontario Health Teams and enabling a better-integrated health system backed by evidence-based tools and digital technology. | In his experience, the Quadruple Aim is a good framework because it treats digital health intervention as no different from other health interventions, and he prefers not making the distinction, but it doesn’t address the adoption question. (eg., [6,7]) |

^a^CPHIMS-CA: Certified Professional in Healthcare Information and Management Systems–Canada

**References**

1. Steele Gray C, Gravesande J, Hans PK, Nie JX, Sharpe S, Loganathan M, Lyons R, Cott C. Using Exploratory Trials to Identify Relevant Contexts and Mechanisms in Complex Electronic Health Interventions: Evaluating the Electronic Patient-Reported Outcome Tool. JMIR Form Res 2019 Feb 27;3(1):e11950. PMID:30810532

2. Bodenheimer T, Sinsky C. From triple to quadruple aim: care of the patient requires care of the provider. Ann Fam Med 2014 Dec;12(6):573–576. PMID:25384822

3. Canada Health Infoway. Benefits Evaluation Framework & Tools. Understanding the effects of Canada’s investments in digital health. Available from: https://www.infoway-inforoute.ca/en/digital-health-initiatives/research-benefits-evaluation/benefits-evaluation-framework-tools [accessed Jan 28, 2023]

4. Nundy S, Cooper LA, Mate KS. The Quintuple Aim for Health Care Improvement: A New Imperative to Advance Health Equity. JAMA 2022 Feb 8;327(6):521–522. doi: 10.1001/jama.2021.25181

5. Itchhaporia D. The Evolution of the Quintuple Aim. J Am Coll Cardiol 2021 Nov 30;78(22):2262–2264. PMID:34823665

6. Laur C, Agarwal P, Thai K, Kishimoto V, Kelly S, Liang K, Bhatia RS, Bhattacharyya O, Martin D, Mukerji G. Implementation and Evaluation of COVIDCare@Home, a Family Medicine–Led Remote Monitoring Program for Patients With COVID-19: Multimethod Cross-sectional Study. JMIR Hum Factors 2022 Jun 28;9(2):e35091. doi: 10.2196/35091

7. Bhatti S, Dahrouge S, Muldoon L, Rayner J. Using the quadruple aim to understand the impact of virtual delivery of care within Ontario community health centres: a qualitative study. BJGP Open 6(4):BJGPO.2022.0031. PMID:36109022
