# Supplementary material for: Mapping Theories, Models, and Frameworks to Evaluate Digital Health Interventions: Scoping Review
Source: J Med Internet Res. 2024 Feb 5;26:e51098. doi: 10.2196/51098 (PMC10877497; doi:10.2196/51098)
Supplement: Multimedia Appendix 2 [file jmir_v26i1e51098_app2.docx]

**Multimedia appendix 2.** Full electronic search strategy

# Sources and search strategy

## Ovid MEDLINE(R) ALL [OVID] <1946 to March 15, 2022>

| 1 | exp Telemedicine/ | 39405 |
| --- | --- | --- |
| 2 | (telemed* or tele-med* or telecare or tele-care or teleconsult* or tele-consult* or telehealth* or tele-health* or telemonitor* or tele-monitor* or telerehab* or tele-rehab*).tw,kf,kw. | 31040 |
| 3 | (ehealth* or e-health* or mhealth* or m-health* or emental health* or e-mental health* or epsychiatr* or e-psychiatr* or epsychol* or e-psychol* or etherap* or e-therap*).tw,kf,kw. | 16987 |
| 4 | (emedicine or e-medicine*).tw,kf,kw. | 97 |
| 5 | (mobile health* or mobile care or mobile medicine).tw,kf,kw. | 6962 |
| 6 | (digital* adj3 (care or health* or healthcare or health-care)).tw,kf,kw. | 7020 |
| 7 | (digital* adj3 (consult* or diagnos* or intervention* or manag* or monitor* or palliat* or rehab* or surger* or surgic* or therap* or treatment*)).tw,kf,tw. | 9138 |
| 8 | (remote* adj3 (consult* or diagnos* or intervention* or manag* or monitor* or palliat* or rehab* or surger* or surgic* or therap* or treatment*)).tw,kf,kw. | 10804 |
| 9 | exp Therapy, Computer-Assisted/ | 43503 |
| 10 | ((internet* or app or apps or computer* or cyber* or digital* or e-application* or e-mail* or email* or electronic mail* or iphone* or i-phone* or (mobile adj2 application*) or mobile-based or mobile phone* or online or smarthome* or smart-home* or smartphone* or smart phone* or technology or technologies or webbased or web-based or webdeliver* or web-deliver* or "web 2.0") adj2 (care or health* or medicine or medical or consult* or diagnos* intervention* or manag* or monitor* or palliat* or rehab* or surger* or surgic* or therap* or treatment*)).tw,kw,kf. | 86626 |
| 11 | Wearable Electronic Devices/ | 5685 |
| 12 | (wearable* adj3 (device* or technology or technologies)).tw,kf,kw. | 8117 |
| 13 | exp Computers/ | 83030 |
| 14 | Electronic Mail/ | 2877 |
| 15 | Internet/ | 78594 |
| 16 | Telecommunications/ | 4992 |
| 17 | or/1-16 | 345995 |
| 18 | *Delivery of Health Care/ | 64684 |
| 19 | ((diffusion or spread* or adoption* or transfer* or implement* or disseminat* or deliver* or distribution* or investment* or develope*) adj4 (facilitator* or barrier* or dimensions or innovat* or technolog* or device* or idea* or app or apps or eclinic* or ehealth or ecare or econsult* or e-clinic* or e-health or e-care or e-consult* or digital health or DHIs)).tw,kf,kw. | 101625 |
| 20 | *Communication Barriers/ | 3255 |
| 21 | *Diffusion of Innovation/ | 8559 |
| 22 | or/18-21 | 174554 |
| 23 | 17 and 22 | 18470 |
| 24 | (evaluat* adj4 (design* or frame or frames or framework* or model or models or theor* or approach* or structur*)).tw,kf,kw. | 201976 |
| 25 | (design* adj3 (frame or frames or framework* or model or models or theor* or stud*)).tw,kf,kw. | 507166 |
| 26 | (apprais* adj3 (frame or frames or framework* or model or models or theor*)).tw,kf,kw. | 1170 |
| 27 | (apprais* adj (design* or guid* or schem* or strateg*)).tw,kf,kw. | 254 |
| 28 | (assess* adj2 (frame or frames or framework* or model or models or theor*)).tw,kf,kw. | 48677 |
| 29 | (assess* adj2 (design* or guid* or schem* or strateg*)).tw,kf,kw. | 38598 |
| 30 | ("framework for implementation research" or CFIR).tw,kf,kw. | 1006 |
| 31 | (evidence-based adj2 (frame or frames or framework* or model or models or theor*)).tw,kf,kw. | 2438 |
| 32 | (evidence-based adj2 (design* or guid* or schem* or strateg*)).tw,kf,kw. | 18666 |
| 33 | (service adj2 (frame or frames or framework* or model or models or theor*)).tw,kw,kf. | 5740 |
| 34 | (approach* adj2 framework*).tw,kw,kf. | 2570 |
| 35 | (service adj2 (design* or guid* or schem* or strateg*)).tw,kf,kw. | 2951 |
| 36 | *Technology Assessment, Biomedical/ or *"Evaluation Studies"/ or *Program Evaluation/ | 17744 |
| 37 | ((evaluat* or assess* or screening or impact* or efficacy or utilization) adj2 (innovation or technolog*)).tw,kw,kf. | 18057 |
| 38 | or/24-37 | 809109 |
| 39 | 23 and 38 | 2886 |
| 40 | limit 39 to yr="2000 -Current" | 2700 |

Haut du formulaire

Bas du formulaire

**Link to the search strategy :** <https://ovidsp.ovid.com/ovidweb.cgi?T=JS&NEWS=N&PAGE=main&SHAREDSEARCHID=GxPz5y2EX0gVQLaxBL2WPJhdExmX3a0sdwfGGsDPSq2l4HwjR0VVmtIKMlvijLsr>

## Embase [OVID] 1974 to 2022 Week 10

| 1 | exp telemedicine/ | 57968 |
| --- | --- | --- |
| 2 | (telemed* or tele-med* or telecare or tele-care or teleconsult* or tele-consult* or telehealth* or tele-health* or telemonitor* or tele-monitor* or telerehab* or tele-rehab*).tw,sh,kw. | 71473 |
| 3 | (ehealth* or e-health* or mhealth* or m-health* or emental health* or e-mental health* or epsychiatr* or e-psychiatr* or epsychol* or e-psychol* or etherap* or e-therap*).tw,sh,kw. | 17770 |
| 4 | (emedicine or e-medicine*).tw,sh,kw. | 162 |
| 5 | (mobile health* or mobile care or mobile medicine).tw,sh,kw. | 8395 |
| 6 | (digital* adj3 (care or health* or healthcare or health-care)).tw,sh,kw. | 10667 |
| 7 | (digital* adj3 (consult* or diagnos* or intervention* or manag* or monitor* or palliat* or rehab* or surger* or surgic* or therap* or treatment*)).tw,sh,tw. | 41936 |
| 8 | (remote* adj3 (consult* or diagnos* or intervention* or manag* or monitor* or palliat* or rehab* or surger* or surgic* or therap* or treatment*)).tw,sh,kw. | 15499 |
| 9 | exp computer assisted therapy/ | 15392 |
| 10 | ((internet* or app or apps or computer* or cyber* or digital* or e-application* or e-mail* or email* or electronic mail* or iphone* or i-phone* or (mobile adj2 application*) or mobile-based or mobile phone* or online or smarthome* or smart-home* or smartphone* or smart phone* or technology or technologies or webbased or web-based or webdeliver* or web-deliver* or "web 2.0") adj2 (care or health* or medicine or medical or consult* or diagnos* intervention* or manag* or monitor* or palliat* or rehab* or surger* or surgic* or therap* or treatment*)).tw,kw,sh. | 498536 |
| 11 | exp wearable computer/ | 6119 |
| 12 | (wearable* adj3 (device* or technology or technologies)).tw,sh,kw. | 8282 |
| 13 | exp computer/ | 149841 |
| 14 | e-mail/ | 26206 |
| 15 | Internet/ | 116580 |
| 16 | telecommunication/ | 26799 |
| 17 | or/1-16 | 808219 |
| 18 | *health care delivery/ | 61871 |
| 19 | ((diffusion or spread* or adoption* or transfer* or implement* or disseminat* or deliver* or distribution* or investment* or develope*) adj4 (facilitator* or barrier* or dimensions or innovat* or technolog* or device* or idea* or app or apps or eclinic* or ehealth or ecare or econsult* or e-clinic* or e-health or e-care or e-consult* or digital health or DHIs)).tw,sh,kw. | 130462 |
| 20 | *communication barrier/ | 636 |
| 21 | *mass communication/ | 6853 |
| 22 | or/18-21 | 197125 |
| 23 | 17 and 22 | 26746 |
| 24 | (evaluat* adj4 (design* or frame or frames or framework* or model or models or theor* or approach* or structur*)).tw,sh,kw. | 270823 |
| 25 | (design* adj3 (frame or frames or framework* or model or models or theor* or stud*)).tw,sh,kw. | 625344 |
| 26 | (apprais* adj3 (frame or frames or framework* or model or models or theor*)).tw,sh,kw. | 1346 |
| 27 | (apprais* adj (design* or guid* or schem* or strateg*)).tw,sh,kw. | 384 |
| 28 | (assess* adj2 (frame or frames or framework* or model or models or theor*)).tw,sh,kw. | 63985 |
| 29 | (assess* adj2 (design* or guid* or schem* or strateg*)).tw,sh,kw. | 53877 |
| 30 | ("framework for implementation research" or CFIR).tw,sh,kw. | 1116 |
| 31 | (evidence-based adj2 (frame or frames or framework* or model or models or theor*)).tw,sh,kw. | 3062 |
| 32 | (evidence-based adj2 (design* or guid* or schem* or strateg*)).tw,sh,kw. | 24961 |
| 33 | (service adj2 (frame or frames or framework* or model or models or theor*)).tw,kw,sh. | 7796 |
| 34 | (approach* adj2 framework*).tw,kw,sh. | 3180 |
| 35 | (service adj2 (design* or guid* or schem* or strateg*)).tw,sh,kw. | 4871 |
| 36 | *biomedical technology assessment/ or *"evaluation study"/ or *program evaluation/ | 9255 |
| 37 | ((evaluat* or assess* or screening or impact* or efficacy or utilization) adj2 (innovation or technolog*)).tw,kw,sh. | 27715 |
| 38 | or/24-37 | 1022232 |
| 39 | 23 and 38 | 3720 |
| 40 | limit 39 to yr="2000 -Current" | 3498 |
| 41 | limit 40 to embase | 1467 |

**Link to the search strategy :** <https://ovidsp.ovid.com/ovidweb.cgi?T=JS&NEWS=N&PAGE=main&SHAREDSEARCHID=1O1e5e1UPN53oUYcVUK4WwnrZEuH5QJ39tWfLAejdJyFLyAzNGL9lAmEhu04gLmhn>

## [OVID] Database Field Guide EBM Reviews - Cochrane Database of Systematic Reviews 2005 to March 9, 2022, Database Field Guide EBM Reviews - ACP Journal Club 1991 to February 2022, Database Field Guide EBM Reviews - Database of Abstracts of Reviews of Effects 1st Quarter 2016, Database Field Guide EBM Reviews - Cochrane Clinical Answers February 2022, Database Field Guide EBM Reviews - Cochrane Central Register of Controlled Trials January 2022, Database Field Guide EBM Reviews - Cochrane Methodology Register 3rd Quarter 2012, Database Field Guide EBM Reviews - Health Technology Assessment 4th Quarter 2016, Database Field Guide EBM Reviews - NHS Economic Evaluation Database 1st Quarter 2016

| 1 | exp Telemedicine/ | 3239 |
| --- | --- | --- |
| 2 | (telemed* or tele-med* or telecare or tele-care or teleconsult* or tele-consult* or telehealth* or tele-health* or telemonitor* or tele-monitor* or telerehab* or tele-rehab*).af. | 9998 |
| 3 | (ehealth* or e-health* or mhealth* or m-health* or emental health* or e-mental health* or epsychiatr* or e-psychiatr* or epsychol* or e-psychol* or etherap* or e-therap*).af. | 4376 |
| 4 | (emedicine or e-medicine*).af. | 71 |
| 5 | (mobile health* or mobile care or mobile medicine).af. | 1762 |
| 6 | (digital* adj3 (care or health* or healthcare or health-care)).af. | 979 |
| 7 | (digital* adj3 (consult* or diagnos* or intervention* or manag* or monitor* or palliat* or rehab* or surger* or surgic* or therap* or treatment*)).af. | 3196 |
| 8 | (remote* adj3 (consult* or diagnos* or intervention* or manag* or monitor* or palliat* or rehab* or surger* or surgic* or therap* or treatment*)).af. | 3261 |
| 9 | exp Therapy, Computer-Assisted/ | 3475 |
| 10 | ((internet* or app or apps or computer* or cyber* or digital* or e-application* or e-mail* or email* or electronic mail* or iphone* or i-phone* or (mobile adj2 application*) or mobile-based or mobile phone* or online or smarthome* or smart-home* or smartphone* or smart phone* or technology or technologies or webbased or web-based or webdeliver* or web-deliver* or "web 2.0") adj2 (care or health* or medicine or medical or consult* or diagnos* intervention* or manag* or monitor* or palliat* or rehab* or surger* or surgic* or therap* or treatment*)).af. | 57804 |
| 11 | Wearable Electronic Devices/ | 121 |
| 12 | (wearable* adj3 (device* or technology or technologies)).af. | 847 |
| 13 | exp Computers/ | 1439 |
| 14 | Electronic Mail/ | 354 |
| 15 | Internet/ | 4223 |
| 16 | Telecommunications/ | 99 |
| 17 | or/1-16 | 74628 |
| 18 | *Delivery of Health Care/ | 1 |
| 19 | ((diffusion or spread* or adoption* or transfer* or implement* or disseminat* or deliver* or distribution* or investment* or develope*) adj4 (facilitator* or barrier* or dimensions or innovat* or technolog* or device* or idea* or app or apps or eclinic* or ehealth or ecare or econsult* or e-clinic* or e-health or e-care or e-consult* or digital health or DHIs)).af. | 12973 |
| 20 | *Communication Barriers/ | 0 |
| 21 | *Diffusion of Innovation/ | 0 |
| 22 | or/18-21 | 12974 |
| 23 | 17 and 22 | 3527 |
| 24 | (evaluat* adj4 (design* or frame or frames or framework* or model or models or theor* or approach* or structur*)).af. | 35922 |
| 25 | (design* adj3 (frame or frames or framework* or model or models or theor* or stud*)).af. | 133453 |
| 26 | (apprais* adj3 (frame or frames or framework* or model or models or theor*)).af. | 134 |
| 27 | (apprais* adj (design* or guid* or schem* or strateg*)).af. | 113 |
| 28 | (assess* adj2 (frame or frames or framework* or model or models or theor*)).af. | 10991 |
| 29 | (assess* adj2 (design* or guid* or schem* or strateg*)).af. | 15454 |
| 30 | ("framework for implementation research" or CFIR).af. | 184 |
| 31 | (evidence-based adj2 (frame or frames or framework* or model or models or theor*)).af. | 636 |
| 32 | (evidence-based adj2 (design* or guid* or schem* or strateg*)).af. | 3497 |
| 33 | (service adj2 (frame or frames or framework* or model or models or theor*)).af. | 885 |
| 34 | (approach* adj2 framework*).af. | 286 |
| 35 | (service adj2 (design* or guid* or schem* or strateg*)).af. | 609 |
| 36 | *Technology Assessment, Biomedical/ or *"Evaluation Studies"/ or *Program Evaluation/ | 0 |
| 37 | ((evaluat* or assess* or screening or impact* or efficacy or utilization) adj2 (innovation or technolog*)).af. | 23936 |
| 38 | or/24-37 | 196766 |
| 39 | 23 and 38 | 1641 |
| 40 | limit 39 to yr="2000 -Current" [Limit not valid in DARE; records were retained] | 1428 |

**Link to the search strategy** : <https://ovidsp.ovid.com/ovidweb.cgi?T=JS&NEWS=N&PAGE=main&SHAREDSEARCHID=6K7xSbIi2iBvN2LsgGdvqwROHKsMvJuQxuqw0US3EaXlapRn6OsvpkuVD0f2vK3aU>

## PsychInfo 1806 to March Week 2 2022 [OVID]

| 1 | exp Telemedicine/ | 10819 |
| --- | --- | --- |
| 2 | (telemed* or tele-med* or telecare or tele-care or teleconsult* or tele-consult* or telehealth* or tele-health* or telemonitor* or tele-monitor* or telerehab* or tele-rehab*).tw,sh,hw,id. | 8203 |
| 3 | (ehealth* or e-health* or mhealth* or m-health* or emental health* or e-mental health* or epsychiatr* or e-psychiatr* or epsychol* or e-psychol* or etherap* or e-therap*).tw,sh,hw,id. | 4354 |
| 4 | (emedicine or e-medicine*).tw,sh,hw,id. | 6 |
| 5 | (mobile health* or mobile care or mobile medicine).tw,sh,hw,id. | 2015 |
| 6 | (digital* adj3 (care or health* or healthcare or health-care)).tw,sh,hw,id. | 1632 |
| 7 | (digital* adj3 (consult* or diagnos* or intervention* or manag* or monitor* or palliat* or rehab* or surger* or surgic* or therap* or treatment*)).tw,sh,tw. | 2257 |
| 8 | (remote* adj3 (consult* or diagnos* or intervention* or manag* or monitor* or palliat* or rehab* or surger* or surgic* or therap* or treatment*)).tw,sh,hw,id. | 1100 |
| 9 | exp Computer Assisted Therapy/ | 11795 |
| 10 | ((internet* or app or apps or computer* or cyber* or digital* or e-application* or e-mail* or email* or electronic mail* or iphone* or i-phone* or (mobile adj2 application*) or mobile-based or mobile phone* or online or smarthome* or smart-home* or smartphone* or smart phone* or technology or technologies or webbased or web-based or webdeliver* or web-deliver* or "web 2.0") adj2 (care or health* or medicine or medical or consult* or diagnos* intervention* or manag* or monitor* or palliat* or rehab* or surger* or surgic* or therap* or treatment*)).tw,sh,hw,id. | 31956 |
| 11 | (wearable* adj3 (device* or technology or technologies)).tw,sh,hw,id. | 1037 |
| 12 | exp Computers/ | 46919 |
| 13 | Electronic Communication/ | 3253 |
| 14 | exp Internet/ | 30755 |
| 15 | Telecommunications Media/ | 1499 |
| 16 | or/1-15 | 109770 |
| 17 | *Health Care Delivery/ | 16869 |
| 18 | ((diffusion or spread* or adoption* or transfer* or implement* or disseminat* or deliver* or distribution* or investment* or develope*) adj4 (facilitator* or barrier* or dimensions or innovat* or technolog* or device* or idea* or app or apps or eclinic* or ehealth or ecare or econsult* or e-clinic* or e-health or e-care or e-consult* or digital health or DHIs)).tw,sh,hw,id. | 28663 |
| 19 | *Communication Barriers/ | 531 |
| 20 | *Communication/ | 22070 |
| 21 | or/17-20 | 66973 |
| 22 | 16 and 21 | 7587 |
| 23 | (evaluat* adj4 (design* or frame or frames or framework* or model or models or theor* or approach* or structur*)).tw,sh,hw,id. | 52853 |
| 24 | (design* adj3 (frame or frames or framework* or model or models or theor* or stud*)).tw,sh,hw,id. | 91596 |
| 25 | (apprais* adj3 (frame or frames or framework* or model or models or theor*)).tw,sh,hw,id. | 2014 |
| 26 | (apprais* adj (design* or guid* or schem* or strateg*)).tw,sh,hw,id. | 274 |
| 27 | (assess* adj2 (design* or guid* or schem* or strateg*)).tw,sh,hw,id. | 19667 |
| 28 | ("framework for implementation research" or CFIR).tw,sh,hw,id. | 217 |
| 29 | (evidence-based adj2 (frame or frames or framework* or model or models or theor*)).tw,sh,hw,id. | 1808 |
| 30 | (evidence-based adj2 (design* or guid* or schem* or strateg*)).tw,sh,hw,id. | 4240 |
| 31 | (service adj2 (frame or frames or framework* or model or models or theor*)).tw,sh,hw,id. | 4775 |
| 32 | (approach* adj2 framework*).tw,sh,hw,id. | 1314 |
| 33 | (service adj2 (design* or guid* or schem* or strateg*)).tw,sh,hw,id. | 2876 |
| 34 | *biomedical technology assessment/ or *"evaluation study"/ or *program evaluation/ | 8941 |
| 35 | ((evaluat* or assess* or screening or impact* or efficacy or utilization) adj2 (innovation or technolog*)).tw,sh,hw,id. | 5392 |
| 36 | or/23-35 | 183146 |
| 37 | 22 and 36 | 965 |
| 38 | limit 37 to yr="2000 -Current" | 933 |

**Link to the search strategy** : <https://ovidsp.ovid.com/ovidweb.cgi?T=JS&NEWS=N&PAGE=main&SHAREDSEARCHID=5BkPhHWu9j9EKwjRD2YJb1fqXuaj7V9cOTNCQGU5iUJNkx3outyaufBVF76Web1dr>

## CINAHL COMPLETE [EBSCO]

| S1 | (MH "Telemedicine+") OR (MH "Computers and Computerization+") OR (MH "Email") OR (MH "Internet") OR (MH "Telecommunications") | 791,004 |
| --- | --- | --- |
| S2 | SU ( ( (telemed* or tele-med* or telecare or tele-care or teleconsult* or tele-consult* or telehealth* or tele-health* or telemonitor* or tele-monitor* or telerehab* or tele-rehab*) ) OR ( (ehealth* or e-health* or mhealth* or m-health* or emental health* or e-mental health* or epsychiatr* or e-psychiatr* or epsychol* or e-psychol* or etherap* or e-therap*) ) OR ( (emedicine or e-medicine*) ) OR ( (mobile health* or mobile care or mobile medicine) ) OR ( (digital* N3 (care or health* or healthcare or health-care)) ) OR ( (digital* N3 (consult* or diagnos* or intervention* or manag* or monitor* or palliat* or rehab* or surger* or surgic* or therap* or treatment*)) ) OR ( (remote* N3 (consult* or diagnos* or intervention* or manag* or monitor* or palliat* or rehab* or surger* or surgic* or therap* or treatment*)) ) OR ( ((internet* or app or apps or computer* or cyber* or digital* or e-application* or e-mail* or email* or electronic mail* or iphone* or i-phone* or (mobile N2 application*) or mobile-based or mobile phone* or online or smarthome* or smart-home* or smartphone* or smart phone* or technology or technologies or webbased or web-based or webdeliver* or web-deliver* or "web 2.0") N2 (care or health* or medicine or medical or consult* or diagnos* intervention* or manag* or monitor* or palliat* or rehab* or surger* or surgic* or therap* or treatment*)) ) ) OR TI ( ( (telemed* or tele-med* or telecare or tele-care or teleconsult* or tele-consult* or telehealth* or tele-health* or telemonitor* or tele-monitor* or telerehab* or tele-rehab*) ) OR ( (ehealth* or e-health* or mhealth* or m-health* or emental health* or e-mental health* or epsychiatr* or e-psychiatr* or epsychol* or e-psychol* or etherap* or e-therap*) ) OR ( (emedicine or e-medicine*) ) OR ( (mobile health* or mobile care or mobile medicine) ) OR ( (digital* N3 (care or health* or healthcare or health-care)) ) OR ( (digital* N3 (consult* or diagnos* or intervention* or manag* or monitor* or palliat* or rehab* or surger* or surgic* or therap* or treatment*)) ) OR ( (remote* N3 (consult* or diagnos* or intervention* or manag* or monitor* or palliat* or rehab* or surger* or surgic* or therap* or treatment*)) ) OR ( ((internet* or app or apps or computer* or cyber* or digital* or e-application* or e-mail* or email* or electronic mail* or iphone* or i-phone* or (mobile N2 application*) or mobile-based or mobile phone* or online or smarthome* or smart-home* or smartphone* or smart phone* or technology or technologies or webbased or web-based or webdeliver* or web-deliver* or "web 2.0") N2 (care or health* or medicine or medical or consult* or diagnos* intervention* or manag* or monitor* or palliat* or rehab* or surger* or surgic* or therap* or treatment*)) ) ) OR AB ( ( (telemed* or tele-med* or telecare or tele-care or teleconsult* or tele-consult* or telehealth* or tele-health* or telemonitor* or tele-monitor* or telerehab* or tele-rehab*) ) OR ( (ehealth* or e-health* or mhealth* or m-health* or emental health* or e-mental health* or epsychiatr* or e-psychiatr* or epsychol* or e-psychol* or etherap* or e-therap*) ) OR ( (emedicine or e-medicine*) ) OR ( (mobile health* or mobile care or mobile medicine) ) OR ( (digital* N3 (care or health* or healthcare or health-care)) ) OR ( (digital* N3 (consult* or diagnos* or intervention* or manag* or monitor* or palliat* or rehab* or surger* or surgic* or therap* or treatment*)) ) OR ( (remote* N3 (consult* or diagnos* or intervention* or manag* or monitor* or palliat* or rehab* or surger* or surgic* or therap* or treatment*)) ) OR ( ((internet* or app or apps or computer* or cyber* or digital* or e-application* or e-mail* or email* or electronic mail* or iphone* or i-phone* or (mobile N2 application*) or mobile-based or mobile phone* or online or smarthome* or smart-home* or smartphone* or smart phone* or technology or technologies or webbased or web-based or webdeliver* or web-deliver* or "web 2.0") N2 (care or health* or medicine or medical or consult* or diagnos* intervention* or manag* or monitor* or palliat* or rehab* or surger* or surgic* or therap* or treatment*)) ) ) | 110,479 |
| S3 | (MH "Therapy, Computer Assisted+") | 20,681 |
| S4 | S1 OR S2 OR S3 | 841,557 |
| S5 | (MM "Health Care Delivery") OR (MM "Communication Barriers") OR (MM "Diffusion of Innovation") | 46,272 |
| S6 | TI ( ((diffusion or spread* or adoption* or transfer* or implement* or disseminat* or deliver* or distribution* or investment* or develope*) N4 (facilitator* or barrier* or dimensions or innovat* or technolog* or device* or idea* or app or apps or eclinic* or ehealth or ecare or econsult* or e-clinic* or e-health or e-care or e-consult* or digital health or DHIs)) ) OR AB ( ((diffusion or spread* or adoption* or transfer* or implement* or disseminat* or deliver* or distribution* or investment* or develope*) N4 (facilitator* or barrier* or dimensions or innovat* or technolog* or device* or idea* or app or apps or eclinic* or ehealth or ecare or econsult* or e-clinic* or e-health or e-care or e-consult* or digital health or DHIs)) ) OR SU ( ((diffusion or spread* or adoption* or transfer* or implement* or disseminat* or deliver* or distribution* or investment* or develope*) N4 (facilitator* or barrier* or dimensions or innovat* or technolog* or device* or idea* or app or apps or eclinic* or ehealth or ecare or econsult* or e-clinic* or e-health or e-care or e-consult* or digital health or DHIs)) ) | 49,732 |
| S7 | S5 OR S6 | 87,215 |
| S8 | S4 AND S7 | 21,135 |
| S9 | (MM "Evaluation Research") OR (MM "Program Evaluation") | 16,066 |
| S10 | TI ( ( (evaluat* N4 (design* or frame or frames or framework* or model or models or theor* or approach* or structur*)) ) OR ( (design* N3 (frame or frames or framework* or model or models or theor* or stud*)) ) OR ( (apprais* N3 (frame or frames or framework* or model or models or theor*)) ) OR ( (apprais* N (design* or guid* or schem* or strateg*)) ) OR ( (assess* N2 (frame or frames or framework* or model or models or theor*)) ) OR ( (assess* N2 (design* or guid* or schem* or strateg*)) ) OR ( ("framework for implementation research" or CFIR) ) OR ( (evidence-based N2 (frame or frames or framework* or model or models or theor*)) ) OR ( (evidence-based N2 (design* or guid* or schem* or strateg*)) ) OR ( (service N2 (frame or frames or framework* or model or models or theor*)) ) OR (approach* N2 framework*) OR ( (service N2 (design* or guid* or schem* or strateg*)) ) ) OR AB ( ( (evaluat* N4 (design* or frame or frames or framework* or model or models or theor* or approach* or structur*)) ) OR ( (design* N3 (frame or frames or framework* or model or models or theor* or stud*)) ) OR ( (apprais* N3 (frame or frames or framework* or model or models or theor*)) ) OR ( (apprais* N (design* or guid* or schem* or strateg*)) ) OR ( (assess* N2 (frame or frames or framework* or model or models or theor*)) ) OR ( (assess* N2 (design* or guid* or schem* or strateg*)) ) OR ( ("framework for implementation research" or CFIR) ) OR ( (evidence-based N2 (frame or frames or framework* or model or models or theor*)) ) OR ( (evidence-based N2 (design* or guid* or schem* or strateg*)) ) OR ( (service N2 (frame or frames or framework* or model or models or theor*)) ) OR (approach* N2 framework*) OR ( (service N2 (design* or guid* or schem* or strateg*)) ) ) OR SU ( ( (evaluat* N4 (design* or frame or frames or framework* or model or models or theor* or approach* or structur*)) ) OR ( (design* N3 (frame or frames or framework* or model or models or theor* or stud*)) ) OR ( (apprais* N3 (frame or frames or framework* or model or models or theor*)) ) OR ( (apprais* N (design* or guid* or schem* or strateg*)) ) OR ( (assess* N2 (frame or frames or framework* or model or models or theor*)) ) OR ( (assess* N2 (design* or guid* or schem* or strateg*)) ) OR ( ("framework for implementation research" or CFIR) ) OR ( (evidence-based N2 (frame or frames or framework* or model or models or theor*)) ) OR ( (evidence-based N2 (design* or guid* or schem* or strateg*)) ) OR ( (service N2 (frame or frames or framework* or model or models or theor*)) ) OR (approach* N2 framework*) OR ( (service N2 (design* or guid* or schem* or strateg*)) ) ) | 310,698 |
| S11 | TI ( ((evaluat* or assess* or screening or impact* or efficacy or utilization) N2 (innovation or technolog*)) ) OR AB ( ((evaluat* or assess* or screening or impact* or efficacy or utilization) N2 (innovation or technolog*)) ) OR SU ( ((evaluat* or assess* or screening or impact* or efficacy or utilization) N2 (innovation or technolog*)) ) | 17,242 |
| S12 | S9 OR S10 OR S11 | 339,517 |
| S13 | S8 AND S12 | 3,756 |
| S14 | S8 AND S12 Date de publication: 20000101-20221231 | 3,687 |

**Link to the search strategy :** <https://tinyurl.com/ye26fxb7>
