# Supplementary material for: Mapping Theories, Models, and Frameworks to Evaluate Digital Health Interventions: Scoping Review
Source: J Med Internet Res. 2024 Feb 5;26:e51098. doi: 10.2196/51098 (PMC10877497; doi:10.2196/51098)
Supplement: Multimedia Appendix 3 [file jmir_v26i1e51098_app3.docx]

**Multimedia Appendix 3.** List of excluded papers and reasons.

# Does not use a framework, model, or theory to implement or evaluate a DHI (n=220)

1. Abd-Alrazaq A, Safi Z, Alajlani M, Warren J, Househ M, Denecke K. Technical metrics used to evaluate health care chatbots: Scoping review. *J Med Internet Res*. 2020;22(6):e18301. doi:10.2196/18301
2. Abdalrahim A, Carter T, Abu Khait A, Clissett P, Blake H. The use of digital touch screen technology to deliver reminiscence therapy among people with dementia in Jordanian care homes: a mixed-method feasibility study. *Psychogeriatrics.* 2022;22(2):187-201. doi:10.1111/psyg.12798
3. Abramson EL, Edwards A, Silver M, Kaushai R, HITEC investigators. Trending health information technology adoption among New York nursing homes. *Am J Manag Care*. 2014;20(11 Spec17):eSP53-9.
4. Ackerman SL, Tebb K, Stein JC, et al. Benefit or burden? A sociotechnical analysis of diagnostic computer kiosks in four California hospital emergency departments. *Soc Sci Med.* 2012;75(12):2378-2385. doi:10.1016/j.socscimed.2012.09.013
5. Adaji A, Schattner P, Piterman L. Web based diabetes care planning - sociotechnical barriers to implementation in general practice. *Aust Fam Physician*. 2011;40(11):915-918.
6. Adepoju IOO, Albersen BJA, De Brouwere V, van Roosmalen J, Zweekhorst M. MHealth for clinical decision-making in sub-Saharan Africa: A scoping review. *JMIR MHealth UHealth.* 2017;5(3):e38. doi:10.2196/mhealth.7185
7. Aji M, Gordon C, Stratton E, et al. Framework for the design engineering and clinical implementation and evaluation of mHealth apps for sleep disturbance: Systematic review. *J Med Internet Res.* 2021;23(2):e24607. doi:10.2196/24607.
8. AlDossary S, Martin-Khan MG, Bradford NK, Smith AC. A systematic review of the methodologies used to evaluate telemedicine service initiatives in hospital facilities. *Int J Med Inform*. 2017;97:171-194. doi:10.1016/j.ijmedinf.2016.10.012
9. Alharbi A, Alzuwaed J, Qasem H. Evaluation of e-health (Seha) application: a cross-sectional study in Saudi Arabia. *BMC Med Inform Decis Mak*. 2021;21(1):103. doi:10.1186/s12911-021-01437-6
10. Almond H, Cummings E, Turner P. Avoiding failure for Australia’s digital health record: The findings from a rural E-health participatory research project. *Stud Health Technol Inform*. 2016;227:8-13.
11. Alodhayani AA, Hassounah MM, Qadri FR, Abouammoh NA, Ahmed Z, Aldahmash AM. Culture-specific observations in a Saudi Arabian digital Home Health Care program: Focus group discussions with patients and their caregivers. *J Med Internet Res.* 2021;23(12):e26002. doi:10.2196/26002
12. Ames HM, Glenton C, Lewin S, Tamrat T, Akama E, Leon N. Clients’ perceptions and experiences of targeted digital communication accessible via mobile devices for reproductive, maternal, newborn, child, and adolescent health: a qualitative evidence synthesis. *Cochrane Database Syst Rev*. 2019;10(10):CD013447. doi:10.1002/14651858.CD013447
13. Amin S, Gupta V, Du G, et al. Developing and demonstrating the viability and availability of the Multilevel Implementation Strategy for Syncope optImal care thrOugh eNgagement (MISSION) syncope app: Evidence-based clinical decision support tool. *J Med Internet Res.* 2021;23(11):e25192. doi:10.2196/25192
14. Ariss SMB, Taylor P, Fitzsimmons D, Kyeremateng S, Mawson S. Mobile technology and delegated work in specialist community services: the EnComPaSS Integration project. *BMJ Support Palliat Care.* Published online 2021:bmjspcare-2020-002288. doi:10.1136/bmjspcare-2020-002288
15. Arnaud N, Bröning S, Drechsel M, Thomasius R, Baldus C. Web-based screening and brief intervention for poly-drug use among teenagers: study protocol of a multicentre two-arm randomized controlled trial. *BMC Public Health.* 2012;12(1):826. doi:10.1186/1471-2458-12-826
16. Ayatollahi H. Patients’ and physicians’ perspectives about using health information technology in diabetes management in Iran: A qualitative study. *Perspectives in Health Information Management*. 2021:1-13.
17. Bakas T, Sampsel D, Israel J, et al. Satisfaction and technology evaluation of a telehealth robotic program to optimize healthy independent living for older adults. *J Nurs Scholarsh.* 2018;50(6):666-675. doi:10.1111/jnu.12436
18. Ballinger C, Taylor A, Loudon D, Macdonald AS. Rehabilitation professionals’ perceptions of the use of new visualisation software tools with people with stroke. *Disabil Rehabil Assist Technol.* 2016;11(2):139-149. doi:10.3109/17483107.2015.1111941
19. Banbury A, Parkinson L, Gordon S, Wood D. Implementing a peer-support programme by group videoconferencing for isolated carers of people with dementia. *J Telemed Telecare.* 2019;25(9):572-577. doi:10.1177/1357633X19873793
20. Bauer MS, Krawczyk L, Miller CJ, et al. Team-based telecare for bipolar disorder. *Telemed J E Health.* 2016;22(10):855-864. doi:10.1089/tmj.2015.0255
21. Beleigoli AM, Andrade AQ, Diniz M de FH, et al. Using the Behaviour Change Wheel for designing an online platform for healthy weight loss - “POEmaS.” *Stud Health Technol Inform.* 2018;254:1-10.
22. Bengtson AM, Kumwenda W, Lurie M, et al. Beyond mobile phones: exploring using technology to support sustained engagement in care for HIV-infected women on antiretroviral therapy. *AIDS Care.* 2020;32(8):959-964. doi:10.1080/09540121.2020.1737639
23. Beresford L, Norwood T. The effect of mobile care delivery on clinically meaningful outcomes, satisfaction, and engagement among physical therapy patients: Observational retrospective study. *JMIR Rehabil Assist Technol.* 2022;9(1):e31349. doi:10.2196/31349
24. Bergin AD, Vallejos EP, Davies EB, et al. Preventive digital mental health interventions for children and young people: a review of the design and reporting of research. *NPJ Digit Med*. 2020;3(1):133. doi:10.1038/s41746-020-00339-7
25. Berlin A, Lovas M, Truong T, et al. Implementation and outcomes of virtual care across a tertiary cancer center during COVID-19. *JAMA Oncol*. 2021;7(4):597-602. doi:10.1001/jamaoncol.2020.6982
26. Bester P, Smit K, De Beer M, Myburgh PH. When online learning becomes compulsory: Student nurses’ adoption of information communication technology in a private nursing education institution. *Curationis.* 2021;44(1):e1-e9. doi:10.4102/curationis.v44i1.2152
27. Biello KB, Hill-Rorie J, Valente PK, et al. Development and evaluation of a mobile app designed to increase HIV testing and pre-exposure prophylaxis use among young men who have sex with men in the United States: Open pilot trial. *J Med Internet Res.* 2021;23(3):e25107. doi:10.2196/25107
28. Bird M, Li L, Ouellette C, Hopkins K, McGillion MH, Carter N. Use of synchronous digital health technologies for the care of children with special health care needs and their families: Scoping review. *JMIR Pediatr Parent.* 2019;2(2):e15106. doi:10.2196/15106
29. Braileanu G, Sa’id J, Higgins C, Rousseau B. Pmu95 clinical trials landscape for digital health interventions: A descriptive analysis. *Value Health*. 2020;23:S250. doi:10.1016/j.jval.2020.04.861
30. Bramley G, Mangan C, Conroy M. Using telemonitoring to support personal care planning for adults with learning disabilities. *J Telemed Telecare*. 2019;25(10):602-610. doi:10.1177/1357633X18784419
31. Briand C, Sablier J, Therrien JA, Charbonneau K, Pelletier JF, Weiss-Lambrou R. Use of a mobile device in mental health rehabilitation: A clinical and comprehensive analysis of 11 cases. *Neuropsychol Rehabil.* 2018;28(5):832-863. doi:10.1080/09602011.2015.1106954
32. Brokel JM, Shaw MG, Nicholson C. Expert clinical rules automate steps in delivering evidence-based care in the electronic health record. Comput Inform Nurs. 2006;24(4):196-205; quiz 206-207. doi:10.1097/00024665-200607000-00005
33. Browne S, Kechadi MT, O’Donnell S, et al. Mobile health apps in pediatric obesity treatment: Process outcomes from a feasibility study of a multicomponent intervention. *JMIR MHealth UHealth.* 2020;8(7):e16925. doi:10.2196/16925
34. Burton M, Lifford KJ, Wyld L, et al. Process evaluation of the Bridging the Age Gap in Breast Cancer decision support intervention cluster randomised trial. *Trials.* 2021;22(1):447. doi:10.1186/s13063-021-05360-z
35. Butler Tobah YS, LeBlanc A, Branda ME, et al. Randomized comparison of a reduced-visit prenatal care model enhanced with remote monitoring. *Am J Obstet Gynecol.* 2019;221(6):638.e1-638.e8. doi:10.1016/j.ajog.2019.06.034
36. Cai RA, Beste D, Chaplin H, et al. Developing and evaluating JIApp: Acceptability and usability of a smartphone app system to improve self-management in young people with juvenile idiopathic arthritis. *JMIR mHealth and uHealth*. 2017;5(8).
37. Camden C, Silva M. Pediatric teleheath: Opportunities created by the COVID-19 and suggestions to sustain its use to support families of children with disabilities. *Phys Occup Ther Pediatr*. 2021;41(1):1-17
38. Camerini L, Schulz PJ. Effects of functional interactivity on patients’ knowledge, empowerment, and health outcomes: an experimental model-driven evaluation of a web-based intervention. *J Med Internet Res*. 2012;14(4):e105. doi:10.2196/jmir.1953
39. Campbell DR, Goldstein H. Evolution of telehealth technology, evaluations, and therapy: Effects of the COVID-19 pandemic on pediatric speech-language pathology services. *Am J Speech Lang Pathol.* 2022;31(1):271-286. doi:10.1044/2021_AJSLP-21-00069
40. Cancela J, Fico G, Arredondo Waldmeyer MT. Using the Analytic Hierarchy Process (AHP) to understand the most important factors to design and evaluate a telehealth system for Parkinson’s disease. *BMC Med Inform Decis Mak*. 2015;15 Suppl 3(S3):S7. doi:10.1186/1472-6947-15-S3-S7
41. Caplan S, Sosa Lovera A, Reyna Liberato P. A feasibility study of a mental health mobile app in the Dominican Republic: The untold story. *Int J Ment Health.* 2018;47(4):311-345. doi:10.1080/00207411.2018.1553486
42. Carlisle N, Watson HA, Carter J, et al. Clinicians’ experiences of using and implementing a medical mobile phone app (QUiPP V2) designed to predict the risk of preterm birth and aid clinical decision making. *BMC Med Inform Decis Mak*. 2021;21(1):320. doi:10.1186/s12911-021-01681-w
43. Carpenter AL, Pincus DB, Furr JM, Comer JS. Working from home: An initial pilot examination of videoconferencing-based cognitive behavioral therapy for anxious youth delivered to the home setting. *Behav Ther.* 2018;49(6):917-930. doi:10.1016/j.beth.2018.01.00740.
44. Cervera Peris M, Alonso Rorís VM, Santos Gago JM, Álvarez Sabucedo L, Wanden-Berghe C, Sanz-Valero J. Management of the general process of parenteral nutrition using mHealth technologies: Evaluation and validation study. *JMIR MHealth UHealth*. 2018;6(4):e79. doi:10.2196/mhealth.9896
45. Charani E, Kyratsis Y, Lawson W, et al. An analysis of the development and implementation of a smartphone application for the delivery of antimicrobial prescribing policy: lessons learnt. *J Antimicrob Chemother*. 2013;68(4):960-967. doi:10.1093/jac/dks492
46. Chávez A, Borrego G, Gutierrez-Garcia JO, Rodríguez LF. Design and evaluation of a mobile application for monitoring patients with Alzheimer’s disease: A day center case study. *Int J Med Inform.* 2019;131(103972):103972. doi:10.1016/j.ijmedinf.2019.103972
47. Chen KY, Harniss M, Patel S, Johnson K. Implementing technology-based embedded assessment in the home and community life of individuals aging with disabilities: a participatory research and development study. *Disabil Rehabil Assist Technol.* 2014;9(2):112-120. doi:10.3109/17483107.2013.805824
48. Chen LC, Chen CW, Weng YC, et al. An information technology framework for strengthening telehealthcare service delivery. *Telemed J E Health*. 2012;18(8):596-603. doi:10.1089/tmj.2011.0267
49. Cheong YL, Rosilawati R, Mohd-Khairuddin CI, et al. PesTrapp mobile app: A trap setting application for real-time entomological field and laboratory study. *Trop Biomed.* 2021;38(2):171-179. doi:10.47665/tb.38.2.054
50. Chin WSY, Kurowski A, Gore R, Chen G, Punnett L, SHIFT Research Team. Use of a mobile app for the process evaluation of an intervention in health care: Development and usability study. *JMIR Form Res*. 2021;5(10):e20739. doi:10.2196/20739
51. Chiu T, Marziali E, Colantonio A, et al. Internet-based caregiver support for Chinese Canadians taking care of a family member with alzheimer disease and related dementia. *Can J Aging.* 2009;28(4):323-336. doi:10.1017/S0714980809990158
52. Choi JS, Lee JH, Park JH, et al. Design and implementation of a seamless and comprehensive integrated medical device interface system for outpatient electronic medical records in a general hospital. *Int J Med Inform*. 2011;80(4):274-285. doi:10.1016/j.ijmedinf.2010.11.007
53. Chung J. *In-home use of home-based sensor technology for monitoring mobility in community-dwelling korean american older adults.*[Order No. 3641504]. University of Washington; 2014
54. Cochran GL, Lander L, Morien M, et al. Consumer opinions of health information exchange, e-prescribing, and personal health records. *Perspect Health Inf Manag*. 2015;12:1e.
55. Cockayne NL, Christensen HM, Griffiths KM, et al. The Sleep Or Mood Novel Adjunctive therapy (SOMNA) trial: a study protocol for a randomised controlled trial evaluating an internet-delivered cognitive behavioural therapy program for insomnia on outcomes of standard treatment for depression in men. *BMC Psychiatry.* 2015;15(1):16. doi:10.1186/s12888-015-0397-x
56. Constantinescu L, Kim J, Feng DD. SparkMed: a framework for dynamic integration of multimedia medical data into distributed m-Health systems. *IEEE Trans Inf Technol Biomed*. 2012;16(1):40-52. doi:10.1109/TITB.2011.2174064
57. Cook EJ, Randhawa G, Large S, Ali N, Chater AM, Guppy A. Satisfaction of using a nurse led telephone helpline among mothers and caregivers of young children. *Health Policy Technol.* 2016;5(2):113-122. doi:10.1016/j.hlpt.2015.12.002
58. Coorey G, Peiris D, Scaria A, et al. An internet-based intervention for cardiovascular disease management integrated with primary care electronic health records: Mixed methods evaluation of implementation fidelity and user engagement. *J Med Internet Res*. 2021;23(4):e25333. doi:10.2196/25333
59. Corazza F, Snijders D, Arpone M, et al. Development and usability of a novel interactive tablet app (PediAppRREST) to support the management of pediatric cardiac arrest: Pilot high-fidelity simulation-based study. *JMIR mHealth and uHealth.* 2020;8(10):1
60. Cottrell MA, Hill AJ, O’Leary SP, Raymer ME, Russell TG. Service provider perceptions of telerehabilitation as an additional service delivery option within an Australian neurosurgical and orthopaedic physiotherapy screening clinic: A qualitative study. *Musculoskelet Sci Pract*. 2017;32:7-16. doi:10.1016/j.msksp.2017.07.008
61. Cowie J, Calveley E, Bowers G, Bowers J. Evaluation of a digital consultation and self-care advice tool in primary care: A multi-methods study. *Int J Environ Res Public Health.* 2018;15(5). doi:10.3390/ijerph15050896
62. Cowie MR, Flett A, Cowburn P, et al. Real-world evidence in a national health service: results of the UK CardioMEMS HF System Post-Market Study. ESC Heart Fail. 2022;9(1):48-56. doi:10.1002/ehf2.13748
63. Cox CE, Olsen MK, Gallis JA, et al. Optimizing a self-directed mobile mindfulness intervention for improving cardiorespiratory failure survivors’ psychological distress (LIFT2): Design and rationale of a randomized factorial experimental clinical trial. *Contemp Clin Trials*. 2020;96(106119):106119. doi:10.1016/j.cct.2020.106119
64. Cresswell K, Worth A, Sheikh A. Implementing and adopting electronic health record systems: How actor-network theory can support evaluation. *Clinical Governance.* 2011;16(4):320-336
65. Dang MT, Whitney KD, Virata MCD, Binger MM, Miller E. A web-based personal health information system for homeless youth and young adults. *Public Health Nurs*. 2012;29(4):313-319. doi:10.1111/j.1525-1446.2011.00998.x
66. Davidson TM, Bunnell BE, Saunders BE, et al. Pilot evaluation of a tablet-based application to improve quality of care in child mental health treatment. *Behav Ther.* 2019;50(2):367-379. doi:10.1016/j.beth.2018.07.005
67. Davis A, Hopkins T, Abrahams Y. Maximizing the impact of telepractice through a multifaceted service delivery model at the shepherd centre, Australia. *The Volta Review*. 2012;112(3):383-391
68. Deady M, Johnston DA, Glozier N, et al. A smartphone application for treating depressive symptoms: study protocol for a randomised controlled trial. *BMC Psychiatry*. 2018;18(1). doi:10.1186/s12888-018-1752-5
69. Deeds SA, Dowdell KJ, Chew LD, Ackerman SL. Implementing an opt-in eConsult program at seven academic medical centers: A qualitative analysis of primary care provider experiences. *J Gen Intern Med*. 2019;34(8):1427-1433. doi:10.1007/s11606-019-05067-7
70. del Río-Lanza AB, Suárez-Vázquez A, Suárez-Álvarez L, Iglesias-Argüelles V. Mobile health (mhealth): facilitators and barriers of the intention of use in patients with chronic illnesses. *J Commun Healthc*. 2020;13(2):138-146. doi:10.1080/17538068.2020.1777513
71. den Haan M, Brankaert R, Kenning G, Lu Y. Creating a social learning environment for and by older adults in the use and adoption of smartphone technology to age in place. Front Public Health. 2021;9:568822. doi:10.3389/fpubh.2021.568822
72. Depatie A, Bigbee JL. Rural older adult readiness to adopt mobile health technology: A descriptive study. *Online J Rural Nurs Health Care*. 2015;15(1):150-184. doi:10.14574/ojrnhc.v15i1.346
73. Derks YP, Klaassen R, Westerhof GJ, Bohlmeijer ET, Noordzij ML. Development of an ambulatory biofeedback app to enhance emotional awareness in patients with borderline personality disorder: Multicycle usability testing study. *JMIR MHealth UHealth*. 2019;7(10):e13479. doi:10.2196/13479
74. Deutscher D, Hart DL, Dickstein R, Horn SD, Gutvirtz M. Implementing an integrated electronic outcomes and electronic health record process to create a foundation for clinical practice improvement. *Phys Ther*. 2008;8(9–12)8(2):270-285. doi:10.2522/ptj.20060280
75. Diez-Canseco F, Toyama M, Ipince A, et al. Integration of a technology-based mental health screening program into routine practices of primary health care services in Peru (the Allillanchu Project): Development and implementation. *J Med Internet Res.* 2018;20(3):e100. doi:10.2196/jmir.9208
76. Dolatabadi E, Zhi YX, Flint AJ, Mansfield A, Iaboni A, Taati B. The feasibility of a vision-based sensor for longitudinal monitoring of mobility in older adults with dementia. *Arch Gerontol Geriatr.* 2019;82:200-206. doi:10.1016/j.archger.2019.02.004
77. Egan KJ, Clark P, Deen Z, et al. Understanding current needs and future expectations of informal caregivers for technology to support health and well-being: National survey study. *JMIR Aging*. 2022;5(1):e15413. doi:10.2196/15413
78. Egede LE, Frueh CB, Richardson LK, et al. Rationale and design: telepsychology service delivery for depressed elderly veterans. *Trials.* 2009;10(1). doi:10.1186/1745-6215-10-22
79. Eisenstein EL, Juzwishin D, Kushniruk AW, Nahm M. Defining a framework for health information technology evaluation. *Stud Health Technol Inform.* 2011;164:94-99.
80. Elnaggar A, von Oppenfeld J, Whooley MA, Merek S, Park LG. Applying mobile technology to sustain physical activity after completion of cardiac rehabilitation: Acceptability study. *JMIR Hum Factors.* 2021;8(3):e25356. doi:10.2196/25356
81. Enrique A, Duffy D, Lawler K, Richards D, Jones S. An internet-delivered self-management programme for bipolar disorder in mental health services in Ireland: Results and learnings from a feasibility trial. *Clin Psychol Psychother*. 2020;27(6):925-939. doi:10.1002/cpp.248080.
82. Escobar MF, Henao JF, Prieto D, et al. Teleconsultation for outpatient care of patients during the Covid-19 pandemic at a University Hospital in Colombia*. Int J Med Inform*. 2021;155(104589):104589. doi:10.1016/j.ijmedinf.2021.104589
83. Evans BA, Beverly CJ, Tsai PF, Rettiganti M, Lefler LL, Parks RF. Older adults’ live demonstration of electronic personal health record use: Factors mediating initial proficiency: Factors mediating initial proficiency. *Comput Inform Nurs.* 2018;36(12):603-609. doi:10.1097/CIN.0000000000000448
84. Everitt N, Broadbent J, Richardson B, et al. Exploring the features of an app-based just-in-time intervention for depression. *J Affect Disord*. 2021;291:279-287. doi:10.1016/j.jad.2021.05.021
85. Fedele DA, McConville A, Moon J, Thomas JG. Topical review: Design considerations when creating pediatric mobile health interventions: Applying the IDEAS framework. *J Pediatr Psychol.* 2019;44(3):343-348. doi:10.1093/jpepsy/jsy084
86. Fenton GL, Smit AK, Freeman L, et al. Development and evaluation of a telephone communication protocol for the delivery of personalized melanoma genomic risk to the general population. *J Genet Couns*. 2018;27(2):370-380. doi:10.1007/s10897-017-0183-7
87. Ferry OR, Moloney EC, Spratt OT, Whiting GFM, Bennett CJ. A virtual ward model of care for patients with COVID-19: Retrospective single-center clinical study. *J Med Internet Res*. 2021;23(2):e25518. doi:10.2196/25518
88. Floren LC, Mandal J, Dall’Era M, et al. A mobile learning module to support interprofessional knowledge construction in the health professions. *Am J Pharm Educ*. 2020;84(2):847519. doi:10.5688/ajpe847519
89. Fiellin LE, Kyriakides TC, Hieftje KD, et al. The design and implementation of a randomized controlled trial of a risk reduction and human immunodeficiency virus prevention videogame intervention in minority adolescents: PlayForward: Elm City Stories*. Clin Trials*. 2016;13(4):400-408. doi:10.1177/1740774516637871
90. Fiks AG, DuRivage N, Mayne SL, et al. Adoption of a portal for the primary care management of pediatric asthma: A mixed-methods implementation study. *J Med Internet Res.* 2016;18(6):e172. doi:10.2196/jmir.5610
91. Finch E, Lethlean J, Rose T, et al. Conversations between people with aphasia and speech pathology students via telepractice: A Phase II feasibility study. *Int J Lang Commun Disord*. 2020;55(1):43-58. doi:10.1111/1460-6984.12501
92. Fisher K, Easton K. The meaning and value of digital technology adoption for older adults with sight loss: A mixed methods study. *Technol Disabil.* 2019;30(4):177-184. doi:10.3233/tad-180205
93. Free C, McCarthy O, French RS, et al. Can text messages increase safer sex behaviours in young people? intervention development and pilot randomised controlled trial. *Health Technol Assess*. 2016;20(57):1-82. doi:10.3310/hta20570
94. Frost EK, Carter SM. Reporting of screening and diagnostic AI rarely acknowledges ethical, legal, and social implications: a mass media frame analysis. *BMC Med Inform Decis Mak*. 2020;20(1):325. doi:10.1186/s12911-020-01353-1
95. Fuemmeler BF, Holzwarth E, Sheng Y, et al. Mila Blooms: A mobile phone application and behavioral intervention for promoting physical activity and a healthy diet among adolescent survivors of childhood cancer. *Games Health.* 2020;9(4):279-289. doi:10.1089/g4h.2019.0060
96. Furness K, Huggins C, Croagh D, Haines T. Exploring the attitudes of health professionals providing care to patients undergoing treatment for upper gastrointestinal cancers to different models of nutrition care delivery: A qualitative investigation. *Nutrients.* 2021;13(3). doi:10.3390/nu13031020
97. Gantner-Bär M, Djanatliev A, Prokosch HU, Sedlmayr M. Conceptual modeling for Prospective Health Technology Assessment. *Stud Health Technol Inform.* 2012;180:33-37.
98. Garrido A, Ramírez López LJ, Álvarez NB. A simulation-based AHP approach to analyze the scalability of EHR systems using blockchain technology in healthcare institutions. *Inform Med Unlocked.* 2021;24(100576):100576. doi:10.1016/j.imu.2021.100576
99. Gaspar AGM, Lapão LV. A digital health service for elderly people with balance disorders and risk of falling: A design science approach. *Int J Environ Res Public Health*. 2022;19(3):1855. doi:10.3390/ijerph19031855
100. Gautham M, Iyengar MS, Johnson CW. Mobile phone-based clinical guidance for rural health providers in India*. Health Informatics J.* 2015;21(4):253-266. doi:10.1177/1460458214523153
101. George TP, DeCristofaro C, Murphy PF, Sims A. Student perceptions and acceptance of mobile technology in an undergraduate nursing program. *Healthcare (Basel)*. 2017;5(3):35. doi:10.3390/healthcare5030035
102. Ginige JA, Maeder AJ, Long V. Evaluating success of mobile health projects in the developing world. *Stud Health Technol Inform*. 2014;206:7-19.
103. Girgis A, Durcinoska I, Levesque JV, et al. EHealth system for collecting and utilizing patient reported outcome measures for personalized treatment and care (PROMPT-care) among cancer patients: Mixed methods approach to evaluate feasibility and acceptability. *J Med Internet Res*. 2017;19(10):e330. doi:10.2196/jmir.8360
104. Giunti G, Kool J, Rivera Romero O, Dorronzoro Zubiete E. Exploring the specific needs of persons with multiple sclerosis for mHealth solutions for physical activity: Mixed-methods study. *JMIR MHealth UHealth*. 2018;6(2):e37. doi:10.2196/mhealth.8996
105. Gómez EJ, Caballero PJ, Malpica N, del Pozo F. Optimisation and evaluation of an asynchronous transfer mode teleradiology co-operative system: the experience of the EMERALD and the BONAPARTE projects. *Comput Methods Programs Biomed.* 2001;64(3):201-214. doi:10.1016/s0169-2607(00)00140-1
106. Gomis-Pastor M, Mirabet S, Roig E, et al. Interdisciplinary mobile health model to improve clinical care after heart transplantation: Implementation strategy study. *JMIR Cardio.* 2020;4(1):e19065. doi:10.2196/19065
107. Graham AK, Greene CJ, Powell T, et al. Lessons learned from service design of a trial of a digital mental health service: Informing implementation in primary care clinics. *Transl Behav Med*. 2020;10(3):598-605. doi:10.1093/tbm/ibz140
108. Green CG. Technology in school nutrition: Challenges in online training. *Top Clin Nutr.* 2008;23(2):187-194. doi:10.1097/01.tin.0000318913.61982.ce
109. Greenhalgh M, Landis JM, Brown J, et al. Assessment of usability and task load demand using a robot-assisted transfer device compared with a Hoyer Advance for dependent wheelchair transfers. *Am J Phys Med Rehabil*. 2019;98(8):729-734. doi:10.1097/PHM.0000000000001176
110. Greenhalgh T. How to improve success of technology projects in health and social care. *Public Health Res Pract.* 2018;28(3). doi:10.17061/phrp2831815
111. Greenhalgh T, Abimbola S. The NASSS framework - A synthesis of multiple theories of technology implementation. *Stud Health Technol Inform.* 2019;263:193-204. doi:10.3233/SHTI190123
112. Greenhalgh T, Shaw S, Wherton J, et al. Real-world implementation of video outpatient consultations at macro, meso, and micro levels: Mixed-method study*. J Med Internet Res*. 2018;20(4):e150. doi:10.2196/jmir.9897
113. Greenwood DA, Gee PM, Fatkin KJ, Peeples M. A systematic review of reviews evaluating technology-enabled diabetes self-management education and support. *J Diabetes Sci Technol.* 2017;11(5):1015-1027. doi:10.1177/1932296817713506
114. Gullslett MK, Nilsen ER, Dugstad J. Next of kin’s experiences with and attitudes towards digital monitoring technology for ageing people with dementia in residential care facilities. A qualitative study based on the voices of next of kin and care providers. *Scand J Caring Sci.* 2022;36(4):1094-1103. doi:10.1111/scs.13009
115. Gwyther H, van Velsen L, Shaw RL, et al. The use of technology in the context of frailty screening and management interventions: a study of stakeholders’ perspectives. *BMC Med Inform Decis Mak*. 2019;19(1):110. doi:10.1186/s12911-019-0828-6
116. Gyldmark M, Lampe K, Ruof J, Pöhlmann J, Hebborn A, Kristensen FB. Is the EUnetHTA hta Core Model® fit for purpose? Evaluation from an industry perspective. *Int J Technol Assess Health Care*. 2018;34(5):458-463. doi:10.1017/S0266462318000594
117. Hadjistavropoulos HD, McCall HC, Thiessen DL, et al. Initial outcomes of transdiagnostic internet-delivered cognitive behavioral therapy tailored to public safety personnel: Longitudinal observational study. *J Med Internet Res.* 2021;23(5):e27610. doi:10.2196/27610
118. Halkett GKB, Jiwa M, Tanner P, Fournier C, Katris P. Trialling computer touch-screen technology to assess psychological distress in patients with gynaecological cancer. *AMJ*. 2010;3(12):781-785. doi:10.4066/AMJ.2010.446
119. Hall A, Brown Wilson C, Stanmore E, Todd C. Moving beyond “safety” versus “autonomy”: a qualitative exploration of the ethics of using monitoring technologies in long-term dementia care. *BMC Geriatr*. 2019;19(1):145. doi:10.1186/s12877-019-1155-6
120. Hamel LM, Thompson HS, Albrecht TL, Harper FW. Designing and testing apps to support patients with cancer: Looking to behavioral science to lead the way. *JMIR Cancer.* 2019;5(1):e12317. doi:10.2196/12317
121. Han J, Oh WO, Park IT, Lee A. Development and effects of a mobile application for safety incident prevention among hospitalized Korean children: A pilot study of feasibility and acceptability. *J Pediatr Nurs*. 2020;51:e69-e76. doi:10.1016/j.pedn.2019.09.022
122. Harris C, Garrubba M, Allen K, et al. Development, implementation and evaluation of an evidence-based program for introduction of new health technologies and clinical practices in a local healthcare setting. *BMC Health Serv Res*. 2015;15:575. doi:10.1186/s12913-015-1178-4
123. Hawley-Hague H, Tacconi C, Mellone S, et al. Smartphone apps to support falls rehabilitation exercise: App development and usability and acceptability study. *JMIR MHealth UHealth*. 2020;8(9):e15460. doi:10.2196/15460
124. Hay JW, Lee PJ, Jin H, et al. Cost-effectiveness of a technology-facilitated depression care management adoption model in safety-net primary care patients with type 2 diabetes. *Value Health*. 2018;21(5):561-568. doi:10.1016/j.jval.2017.11.005
125. Heideman WH, Rongen FC, Bolleurs C, Govers E, Kroeze W, Steenhuis IHM. Facilitators and barriers to a dietitian-implemented blended care weight-loss intervention (SMARTsize): a qualitative study*. J Hum Nutr Diet.* 2019;32(3):338-348. doi:10.1111/jhn.12641
126. Helzlsouer KJ, Appling SE, Scarvalone S, Manocheh S, MacDonald R. Gallicchio L, et al. Development and evaluation of a technology-enhanced interdisciplinary navigation program for low-income breast cancer patients. *Journal of AONN+*. 2016;7(4).
127. Hightow-Weidman LB, Muessig K, Rosenberg E, et al. University of North Carolina/Emory Center for Innovative Technology (iTech) for addressing the HIV epidemic among adolescents and young adults in the United States: Protocol and rationale for center development. *JMIR Res Protoc.* 2018;7(8):e10365. doi:10.2196/10365
128. Hightow-Weidman LB, Pike E, Fowler B, et al. Healthmpowerment.org: Feasibility and acceptability of delivering an internet intervention to young Black men who have sex with men. *AIDS Care.* 2012;24(7):910-920. doi:10.1080/09540121.2011.647677
129. Ho K, Bloch R, Gondocz T, et al. Technology-enabled knowledge translation: frameworks to promote research and practice. *J Contin Educ Health Prof.* 2004;24(2):90-99. doi:10.1002/chp.1340240206
130. Hobson GR, Caffery LJ, Neuhaus M, Langbecker DH. Mobile health for First Nations populations: Systematic review. *JMIR MHealth UHealth*. 2019;7(10):e14877. doi:10.2196/14877
131. Hogaboam L, Daim T. Technology adoption potential of medical devices: The case of wearable sensor products for pervasive care in neurosurgery and orthopedics. *Health Policy Technol.* 2018;7(4):409-419. doi:10.1016/j.hlpt.2018.10.011
132. Hogaboam LS. *Assessment of technology adoption potential of medical devices: Case of wearable sensor products for pervasive care in neurosurgery and orthopedics.*[Order No. AAI10750199]. Portland State University; 2018.
133. Hollis C, Falconer CJ, Martin JL, et al. Annual Research Review: Digital health interventions for children and young people with mental health problems - a systematic and meta-review. *J Child Psychol Psychiatry*. 2017;58(4):474-503. doi:10.1111/jcpp.12663
134. Hsieh KL, Fanning JT, Rogers WA, Wood TA, Sosnoff JJ. A fall risk mHealth app for older adults: Development and usability study. *JMIR Aging*. 2018;1(2):e11569. doi:10.2196/11569
135. Huang J. Innovative health care delivery system--a questionnaire survey to evaluate the influence of behavioral factors on individuals' acceptance of telecare. *Comput Biol Med*. 2013;43(4):281-286. doi:10.1016/j.compbiomed.2012.12.011.
136. Hungerbuehler I, Leite RFM, Bilt MTV de, Gattaz WF. A randomized clinical trial of home-based telepsychiatric outpatient care via videoconferencing: design, methodology, and implementation. *Rev Psiquiatr Clin*. 2015;42(3):76-78. doi:10.1590/0101-60830000000052
137. Hunt CW, Henderson K, Chapman R. Using technology to provide diabetes education for rural communities. *Online J Rural Nurs Health Care*. 2018;18(2):134-151. doi:10.14574/ojrnhc.v18i2.525
138. Huser V, Rasmussen LV, Oberg R, Starren JB. Implementation of workflow engine technology to deliver basic clinical decision support functionality. *BMC Med Res Methodol*. 2011;11(1):43. doi:10.1186/1471-2288-11-43
139. Hutting N, Detaille SI, Heerkens YF, Engels JA, Staal JB, Nijhuis-Van der Sanden MWG. Experiences of participants in a self-management program for employees suffering from complaints of the arm, neck or shoulder (CANS). *Physiotherapy.* 2015;101:e619. doi:10.1016/j.physio.2015.03.3453
140. Jalil S, Myers T, Atkinson I, Soden M. Complementing a clinical trial with human-computer interaction: Patients’ user experience with telehealth. *JMIR Hum Factors.* 2019;6(2):e9481. doi:10.2196/humanfactors.9481
141. Jamshidnezhad A, Jazayeri SMHM, Dehaghi BF, Shoushtarian AT. A native mobile-based app for nutritional self-care behavior assessment. *Progress in Nutrition.* 2021; 23(3). doi:10.23751/pn.v23i3.10637
142. Jang S, Kim JJ, Kim SJ, Hong J, Kim S, Kim E. Mobile app-based chatbot to deliver cognitive behavioral therapy and psychoeducation for adults with attention deficit: A development and feasibility/usability study. *Int J Med Inform.* 2021;150(104440):104440. doi:10.1016/j.ijmedinf.2021.104440
143. Jennett P, Watanabe M. Healthcare and telemedicine: Ongoing and evolving challenges. *Dis Manag Health Outcomes.* 2006;14. doi:10.2165/00115677-200614001-00004
144. Jeon E, Park HA. Development of the IMB model and an evidence-based diabetes self-management mobile application. *Healthc Inform Res*. 2018;24(2):125. doi:10.4258/hir.2018.24.2.125
145. Johansson L, Hagman E, Danielsson P. A novel interactive mobile health support system for pediatric obesity treatment: a randomized controlled feasibility trial. *BMC Pediatr*. 2020;20(1):447. doi:10.1186/s12887-020-02338-9
146. Joseph RP, Ainsworth BE, Vega-López S, et al. Rationale and design of smart walk: A randomized controlled pilot trial of a smartphone-delivered physical activity and cardiometabolic risk reduction intervention for african american women. *Contemporary Clinical Trials.* 2019;77:46-60. doi: 10.1016/j.cct.2018.12.011.
147. Kalhori SRN, Hemmat M, Noori T, Heydarian S, Katigari MR. Quality evaluation of English mobile applications for gestational diabetes: App review using mobile application rating scale (MARS). *Curr Diabetes Rev*. 2021;17(2):161-168. doi:10.2174/1573399816666200703181438
148. Kaplan B. Evaluation of people, social, and organizational issues - sociotechnical ethnographic evaluation. *Stud Health Technol Inform*. 2016;222:114-125.
149. Kaufman DR, Starren J, Patel VL, et al. A cognitive framework for understanding barriers to the productive use of a diabetes home telemedicine system. *AMIA Annu Symp Proc*. Published online 2003:356-360.
150. Kearney SM, Williams K, Nikolajski C, et al. Stakeholder impact on the implementation of integrated care: Opportunities to consider for patient-centered outcomes research. *Contemp Clin Trials.* 2021;101(106256):106256. doi:10.1016/j.cct.2020.106256
151. Kim S, Choudhury A. Comparison of older and younger adults’ attitudes toward the adoption and use of activity trackers. *JMIR MHealth UHealth*. 2020;8(10):e18312. doi:10.2196/18312
152. Kizakevich PN, Eckhoff R, Weger S, et al. A personal health information toolkit for health intervention research. *Stud Health Technol Inform*. 2014;199:35-39.
153. Koivumäki T, Pekkarinen S, Lappi M, Väisänen J, Juntunen J, Pikkarainen M. Consumer adoption of future MyData-based preventive eHealth services: An acceptance model and survey study. *J Med Internet Res*. 2017;19(12):e429. doi:10.2196/jmir.7821
154. Koivunen M, Välimäki M, Pitkänen A, Kuosmanen L. A preliminary usability evaluation of Web-based portal application for patients with schizophrenia. *J Psychiatr Ment Health Nurs.* 2007;14(5):462-469. doi:10.1111/j.1365-2850.2007.01111.x
155. Korinek EV, Phatak SS, Martin CA, et al. Adaptive step goals and rewards: a longitudinal growth model of daily steps for a smartphone-based walking intervention. *J Behav Med*. 2018;41(1):74-86. doi:10.1007/s10865-017-9878-3
156. Kowalski R, Capan M, Lodato P, et al. Optimizing usability and signal capture: a proactive risk assessment for the implementation of a wireless vital sign monitoring system. *J Med Eng Technol.* 2017;41(8):623-629. doi:10.1080/03091902.2017.1382589
157. Kruszyńska-Fischbach A, Sysko-Romańczuk S, Rafalik M, Walczak R, Kludacz-Alessandri M. Organizational E-readiness for the digital transformation of primary healthcare providers during the COVID-19 pandemic in Poland. *J Clin Med.* 2021;11(1):133. doi:10.3390/jcm11010133
158. Kumar S, Nilsen WJ, Abernethy A, et al. Mobile health technology evaluation: the mHealth evidence workshop. *Am J Prev Med*. 2013;45(2):228-236. doi:10.1016/j.amepre.2013.03.017
159. Kuziemsky CE. A model of tradeoffs for understanding health information technology implementation. *Stud Health Technol Inform*. 2015;215:116-128.
160. LaMonica HM, Iorfino F, Lee GY, et al. Informing the future of integrated digital and clinical mental health care: Synthesis of the outcomes from Project Synergy. *JMIR Ment Health.* 2022;9(3):e33060. doi:10.2196/33060
161. Langhan ML, Riera A, Kurtz JC, Schaeffer P, Asnes AG. Implementation of newly adopted technology in acute care settings: a qualitative analysis of clinical staff. *J Med Eng Technol*. 2015;39(1):44-53. doi:10.3109/03091902.2014.973618
162. Lapão LV, Peyroteo M, Maia M, et al. Implementation of digital monitoring services during the COVID-19 pandemic for patients with chronic diseases: Design Science approach. *J Med Internet Res.* 2021;23(8):e24181. doi:10.2196/24181160.
163. Laureij LT, Breunis LJ, Steegers-Theunissen RPM, Rosman AN. Identifying the needs for a web-based postpartum platform among parents of newborns and health care professionals: Qualitative focus group study. *JMIR Form Res*. 2020;4(5):e16202. doi:10.2196/16202
164. Lim J, Cloete G, Dunsmuir DT, et al. Usability and feasibility of PIERS on the Move: An mHealth app for pre-eclampsia triage. *JMIR MHealth UHealth.* 2015;3(2):e37. doi:10.2196/mhealth.3942
165. MacLure K, Stewart D. From policy towards pharmacy practice: A review of the intended use of ehealth in pharmacy in Scotland. *Health Policy Technol.* 2017;6(3):279-285. doi:10.1016/j.hlpt.2017.07.004
166. Magoc D, Tomaka J, Bridges-Arzaga A. Using the web to increase physical activity in college students. *Am J Health Behav*. 2011;35(2):142-154. doi:10.5993/ajhb.35.2.2
167. Marshall JM, Dunstan DA, Bartik W. Apps with maps-anxiety and depression mobile apps with evidence-based frameworks: Systematic search of major app stores. *JMIR Ment Health.* 2020;7(6):e16525. doi:10.2196/16525
168. Mayberry LS, Lyles CR, Oldenburg B, Osborn CY, Parks M, Peek ME. MHealth interventions for disadvantaged and vulnerable people with type 2 diabetes. *Curr Diab Rep.* 2019;19(12):148. doi:10.1007/s11892-019-1280-9
169. Mehta S, Ahrens J, Abu-Jurji Z, et al. Feasibility of a virtual service delivery model to support physical activity engagement during the COVID-19 pandemic for those with spinal cord injury. *J Spinal Cord Med*. 2021;44:S256-S265. doi:10.1080/10790268.2021.1970885.
170. Meinert E, Milne-Ives M, Surodina S, Lam C. Agile requirements engineering and software planning for a digital health platform to engage the effects of isolation caused by social distancing: Case study. *JMIR Public Health Surveill*. 2020;6(2):e19297. doi:10.2196/19297
171. Mekonnen ZA, Gelaye KA, Were MC, Tilahun B. Acceptability, barriers and facilitators of mobile text message reminder system implementation in improving child vaccination: A qualitative study in northwest Ethiopia. *J Multidiscip Healthc*. 2021;14:605-616. doi:10.2147/JMDH.S298167
172. Melchiorre MG, Papa R, Quattrini S, Lamura G, Barbabella F, on behalf of ICARE4EU Consortium. Integrated care programs for people with multimorbidity in European countries: eHealth adoption in health systems. *Biomed Res Int*. 2020;2020:9025326. doi:10.1155/2020/9025326
173. Mendez I, Jong M, Keays-White D, Turner G. The use of remote presence for health care delivery in a northern Inuit community: a feasibility study. *Int J Circumpolar Health.* 2013;72(1):21112. doi:10.3402/ijch.v72i0.21112
174. Michel-Verkerke MB, Spil TAM. The USE IT-adoption-model to predict and evaluate adoption of information and communication technology in healthcare. *Methods Inf Med*. 2013;52(6):475-483. doi:10.3414/ME12-01-0107
175. Miranda J, Cabral J, Wagner S, et al. An open platform for seamless sensor support in healthcare for the internet of things*. Sensors (Basel).* 2016;16(12):2089. doi:10.3390/s16122089
176. Moeckli J, Cram P, Cunningham C, Reisinger HS. Staff acceptance of a telemedicine intensive care unit program: a qualitative study. *J Crit Care.* 2013;28(6):890-901. doi:10.1016/j.jcrc.2013.05.008
177. 178. Moehr JR, Schaafsma J, Anglin C, Pantazi SV, Grimm NA, Anglin S. Success factors for telehealth--a case study. *Int J Med Inf*. 2006;75(10-11):755-763.
178. Montanaro C, Lacey L, Robson L, Estill A, Vukovic S. Preconception care: A technology-based model for delivery in the primary care setting supported by public health. *Matern Child Health J.* 2019;23(12):1581-1586. doi:10.1007/s10995-019-02806-4
179. Mueller M, Knop M, Niehaves B, Adarkwah CC. Investigating the acceptance of video consultation by patients in rural primary care: Empirical comparison of preusers and actual users. *JMIR Med Inform*. 2020;8(10):e20813. doi:10.2196/20813
180. Neffa-Creech D, Clarke P, Evans SH, Glovinsky J. “Food is something everyone should participate in”: A positive deviance approach to understanding the use of a food and nutrition app in low-income, Latino homes. *SAGE Open Med*. 2020;8:2050312120934842. doi:10.1177/2050312120934842
181. Negrini S, Donzelli S, Negrini A, Negrini A, Romano M, Zaina F. Feasibility and acceptability of telemedicine to substitute outpatient rehabilitation services in the COVID-19 emergency in Italy: An observational everyday clinical-life study. *Arch Phys Med Rehabil*. 2020;101(11):2027-2032. doi:10.1016/j.apmr.2020.08.001
182. Ng SL, Phelan S, Leonard M, Galster J. A qualitative case study of smartphone-connected hearing aids: Influences on patients, clinicians, and patient-clinician interactions. *J Am Acad Audiol.* 2017;28(6):506-521. doi:10.3766/jaaa.15153
183. Nielsen C, Agerskov H, Bistrup C, Clemensen J. Evaluation of a telehealth solution developed to improve follow-up after kidney transplantation. *J Clin Nurs.* 2020;29(7-8):1053-1063. doi:10.1111/jocn.15178
184. Nitsch M, Dimopoulos CN, Flaschberger E, et al. A guided online and mobile self-help program for individuals with eating disorders: An iterative engagement and usability study. *J Med Internet Res.* 2016;18(1):e7. doi:10.2196/jmir.4972
185. Opoku-Agyeman E. Development and usability testing of a mobile health game application for older adults on warfarin. Dissertation Abstracts International: Section B: The Sciences and Engineering 2021;82(8-B):No Pagination Specified
186. Ormel I, Onu CC, Magalhaes M, Tang T, Hughes JB, Law S. Using a mobile app-based video recommender system of patient narratives to prepare women for breast cancer surgery: Development and usability study informed by qualitative data. *JMIR Form Res*. 2021;5(6):e22970. doi:10.2196/22970
187. Parmar B, Beukes E, Rajasingam S. The impact of COVID-19 on provision of UK audiology services & on attitudes towards delivery of telehealth services. *Int J Audiol*. 2022;61(3):228-238. doi:10.1080/14992027.2021.1921292
188. Peleg M, Michalowski W, Wilk S, et al. Ideating mobile health behavioral support for compliance to therapy for patients with chronic disease: A case study of atrial fibrillation management. *J Med Syst*. 2018;42(11):234. doi:10.1007/s10916-018-1077-4
189. Petracca F, Tempre R, Cucciniello M, et al. An electronic patient-reported outcome mobile app for data collection in type A hemophilia: Design and usability study. *JMIR Form Res.* 2021;5(12):e25071. doi:10.2196/25071
190. Petrozzi MJ, Spencer G, Mackey MG. A process evaluation of the Mind Your Back trial examining psychologically informed physical treatments for chronic low back pain. *Chiropr Man Therap*. 2021;29(1):32. doi:10.1186/s12998-021-00389-y
191. Pinnock H, Adlem L, Gaskin S, Harris J, Snellgrove C, Sheikh A. Accessibility, clinical effectiveness, and practice costs of providing a telephone option for routine asthma reviews: phase IV controlled implementation study. *Br J Gen Pract.* 2007;57(542):714-722.
192. Free C, Phillips G, Watson L, et al. The effectiveness of mobile-health technologies to improve health care service delivery processes: A systematic review and meta-analysis. *PLoS Med*. 2013;10(1):e1001363. doi:10.1371/journal.pmed.1001363
193. Salari R, R Niakan Kalhori S, GhaziSaeedi M, Jeddi M, Nazari M, Fatehi F. Mobile-based and cloud-based system for self-management of people with type 2 diabetes: Development and usability evaluation. *J Med Internet Res*. 2021;23(6):e18167. doi:10.2196/18167
194. Samoutis G, Soteriades E, Kounalakis D, Zachariadou T, Philalithis A, Lionis C. Implementation of an electronic medical record system in previously computer-naïve primary care centres: a pilot study from Cyprus. *J Innov Health Inform.* 2008;15(4):207-216. doi:10.14236/jhi.v15i4.660
195. Sassenberg K, Roesel I, Sudeck G, Bernecker K, Durst J, Krauss I. The relation of attitude toward technology and mastery experience after an app-guided physical exercise intervention: Randomized crossover trial. *JMIR Form Res.* 2022;6(2):e28913. doi:10.2196/28913
196. Sheerman JFM, van Empelen P, van Loveren C, van Meijel B. A mobile app (WhiteTeeth) to promote good oral health behavior among dutch adolescents with fixed orthodontic appliances: Intervention mapping approach. *JMIR mHealth and uHealth.* 2018;6(8). doi:10.2196/mhealth.9626.
197. Schubart JR, Einbinder JS. Evaluation of a data warehouse in an academic health sciences center. *Int J Med Inform*. 2000;60(3):319-333. doi:10.1016/s1386-5056(00)00126-x
198. Scott IA, Sullivan C, Staib A. Going digital: a checklist in preparing for hospital-wide electronic medical record implementation and digital transformation. *Aust Health Rev*. 2019;43(3):302-313. doi:10.1071/AH17153
199. Seabrook HJ. *Informal learning using tablet computers and apps: A multi-method study of older adults self-managing diabetes.*[Order No. AAI10296354]. University of Calgary (Canada); 2017.
200. Selman L, McDermott K, Donesky D, Citron T, Howie-Esquivel J. Appropriateness and acceptability of a Tele-Yoga intervention for people with heart failure and chronic obstructive pulmonary disease: qualitative findings from a controlled pilot study. *BMC Complement Altern Med*. 2015;15(1):21. doi:10.1186/s12906-015-0540-8
201. Short CE, Finlay A, Sanders I, Maher C. Development and pilot evaluation of a clinic-based mHealth app referral service to support adult cancer survivors increase their participation in physical activity using publicly available mobile apps. *BMC Health Serv Res.* 2018;18(1):27. doi:10.1186/s12913-017-2818-7
202. Sittig DF, Kahol K, Singh H. Sociotechnical evaluation of the safety and effectiveness of point-of-care mobile computing devices: a case study conducted in India. *Stud Health Technol Inform.* 2013;192:515-519.
203. Sockolow P, Yang Y. Preparing for the Improving Medicare Post-Acute Care Transformation Act: Incorporating A sociotechnical perspective to enhance a health information technology Evaluation Framework applied to post-acute care*. Comput Inform Nurs.* 2021;39(11):813-820. doi:10.1097/CIN.0000000000000782
204. Sridharan V, Shoda Y, Heffner J, Bricker J. A pilot randomized controlled trial of a Web-based growth mindset intervention to enhance the effectiveness of a smartphone app for smoking cessation. *JMIR MHealth UHealth*. 2019;7(7):e14602. doi:10.2196/14602
205. Still CH, Margevicius S, Harwell C, et al. A community and technology-based approach for hypertension self-management (COACHMAN) to improve blood pressure control in African Americans: Results from a pilot study. *Patient Prefer Adherence.* 2020;14:2301-2313. doi:10.2147/PPA.S283086
206. Stricklin MLV, Struk CM. Point of care technology: a sociotechnical approach to home health implementation. *Methods Inf Med.* 2003;42(4):463-470.
207. Su JJ, Yu DSF. Effects of a nurse-led eHealth cardiac rehabilitation programme on health outcomes of patients with coronary heart disease: A randomised controlled trial. *Int J Nurs Stud.* 2021;122(104040):104040. doi:10.1016/j.ijnurstu.2021.104040
208. Sun CJ, Anderson KM, Kuhn T, Mayer L, Klein CH. A sexual health promotion app for transgender women (Trans Women Connected): Development and usability study. *JMIR MHealth UHealth.* 2020;8(5):e15888. doi:10.2196/15888
209. Svedberg P, Arvidsson S, Larsson I, Carlsson IM, Nygren JM. Barriers and enablers affecting successful implementation of the electronic health service Sisom: Multicenter study of child participation in pediatric care. *J Med Internet Res*. 2019;21(11):e14271. doi:10.2196/14271
210. Swanepoel DW. EHealth technologies enable more accessible hearing care. *Semin Hear.* 2020;41(2):133-140. doi:10.1055/s-0040-1708510
211. Taylor S, Allsop MJ, Bekker HL, Bennett MI, Bewick BM. Identifying professionals’ needs in integrating electronic pain monitoring in community palliative care services: An interview study*. Palliat Med.* 2017;31(7):661-670. doi:10.1177/0269216316677470
212. Teo CH, Ng CJ, Lo SK, Lim CD, White A. A mobile Web app to improve health screening uptake in men (ScreenMen): Utility and usability evaluation study. *JMIR MHealth UHealth. 2019*;7(4):e10216. doi:10.2196/10216
213. Tozour JN, Bandremer S, Patberg E, et al. Application of telemedicine video visits in a maternal-fetal medicine practice at the epicenter of the COVID-19 pandemic. *Am J Obstet Gynecol MFM.* 2021;3(6):100469. doi:10.1016/j.ajogmf.2021.100469
214. Tsai CH, Koch S. Towards a framework for national eHealth evaluation and monitoring: A combined top-down and bottom-up approach using Sweden as example. *Stud Health Technol Inform.* 2019;264:954-958. doi:10.3233/SHTI190365
215. Vaithamanithi R, Raj M, Vannan M. Adoption of health information technology by private medical practitioners in India. *IJPSR*. 2016;36(1):44-49.
216. Valentine SE, Fuchs C, Godfrey L, Elwy AR. Redesign of a brief PTSD treatment in safety net integrated primary care: Supporting implementation in the context of the COVID-19 pandemic. *Gen Hosp Psychiatry*. 2022;74:94-101. doi:10.1016/j.genhosppsych.2021.12.004
217. Van Velthoven MH, Cordon C. Sustainable adoption of digital health innovations: Perspectives from a stakeholder workshop. *J Med Internet Res*. 2019;21(3):e11922. doi:10.2196/11922
218. Vugts MA, Zedlitz AM, Joosen MC, Vrijhoef HJ. Serious gaming during multidisciplinary rehabilitation for patients with chronic pain or fatigue symptoms: Mixed methods design of a realist process evaluation. *J Med Internet Res.* 2020;22(3):e14766. doi:10.2196/14766
219. Wheelahan CJ. *Innovation ecosystems for health: A learning approach to public health implementation.*[Order No. AAI28223239]. Harvard University; 2021.
220. Zaidi STR, Thursky KA. Using formative evaluation to improve uptake of a web-based tool to support antimicrobial stewardship. *J Clin Pharm Ther.* 2013;38(6):490-497. doi:10.1111/jcpt.12093

# 12. Describes a collection of DHIs (not a singular DHI) (n=104)

1. Ahmadi H, Nilashi M, Ibrahim O. Organizational decision to adopt hospital information system: an empirical investigation in the case of Malaysian public hospitals. *Int J Med Inform*. 2015;84(3):166-188. doi:10.1016/j.ijmedinf.2014.12.004
2. Alaboudi A, Atkins A, Sharp B, Balkhair A, Alzahrani M, Sunbul T. Barriers and challenges in adopting Saudi telemedicine network: The perceptions of decision makers of healthcare facilities in Saudi Arabia. *J Infect Public Health.* 2016;9(6):725-733. doi:10.1016/j.jiph.2016.09.001
3. Alami, H., Fortin, J. P., Gagnon, M. P., Lamothe, L., Ghandour, E. K., Ag Ahmed, M. A. and Roy, D. Strategic framework to support the evaluation of complex and innovative digital health projects. *Sante Publique*. 2020;32(2):221-228.
4. Allen CG, Ritchie JB, Morrison H, et al. A thematic analysis of health information technology use among cancer genetic counselors. *J Genet Couns*. 2021;30(1):170-179. doi:10.1002/jgc4.1306
5. Alzahrani, S., Daim, T., & Choo, K. K. R. Assessment of the blockchain technology adoption for the management of the electronic health record systems. *IEEE Transactions on Engineering Management*. 2022.
6. Azar KMJ, Bennett GG, Nolting LA, Rosas LG, Burke LE, Ma J. A framework for examining the function of digital health technologies for weight management. *Transl Behav Med.* 2018;8(2):280-294. doi:10.1093/tbm/ibx050
7. Baltaxe E, Czypionka T, Kraus M, et al. Digital health transformation of integrated care in Europe: Overarching analysis of 17 integrated care programs. *J Med Internet Res*. 2019;21(9):e14956. doi:10.2196/14956
8. Bhattarai P, Newton-John TRO, Phillips JL. Quality and usability of arthritic pain self-management apps for older adults: A systematic review. *Pain Med.* 2018;19(3):471-484. doi:10.1093/pm/pnx090
9. Briz-Ponce L, García-Peñalvo FJ. An empirical assessment of a technology acceptance model for apps in medical education. *J Med Syst*. 2015;39(11):176. doi:10.1007/s10916-015-0352-x12.
10. Broomhead SC, Mars M, Scott RE, Jones T. Applicability of the five case model to African eHealth investment decisions. *BMC Health Serv Res*. 2020;20(1):666. doi:10.1186/s12913-020-05526-6
11. Brown W 3rd, Yen PY, Rojas M, Schnall R. Assessment of the Health IT Usability Evaluation Model (Health-ITUEM) for evaluating mobile health (mHealth) technology. *J Biomed Inform*. 2013;46(6):1080-1087. doi:10.1016/j.jbi.2013.08.001
12. Bruce, K. Use of mobile technology for monitoring and evaluation in international health and development programs. Doctoral dissertation, The University of North Carolina at Chapel Hill. 2013.
13. Bucci, S., Berry, N., Morris, R., Berry, K., Haddock, G., Lewis, S., & Edge, D. “They are not hard-to-reach clients. We have just got hard-to-reach services.” Staff views of digital health tools in specialist mental health services. *Front. Psychiatry*. 2019;10, 344.
14. Buccoliero L, Calciolari S, Marsilio M. A methodological and operative framework for the evaluation of an e-health project. *Int J Health Plann Manage*. 2008;23(1):3-20. doi:10.1002/hpm.881
15. Char DS, Abràmoff MD, Feudtner C. Identifying ethical considerations for machine learning healthcare applications. *Am J Bioeth*. 2020;20(11):7-17. doi:10.1080/15265161.2020.1819469
16. Cheng VWS, Piper SE, Ottavio A, Davenport TA, Hickie IB. Recommendations for designing health information technologies for mental health drawn from self-determination theory and co-design with culturally diverse populations: Template analysis. *J Med Internet Res.* 2021;23(2):e23502. doi:10.2196/23502
17. Cherrez-Ojeda I, Vanegas E, Felix M, et al. Frequency of use, perceptions and barriers of information and communication technologies among Latin American physicians: An Ecuadorian cross-sectional study. *J Multidiscip Healthc.* 2020;13:259-269. doi:10.2147/JMDH.S246253
18. Chi C, Lee JL, Schoon R. Assessing health information technology in a national health care system--an example from Taiwan. *Adv Health Care Manag*. 2012;12:75-109. doi:10.1108/s1474-8231(2012)0000012008
19. Christie HL, Bartels SL, Boots LMM, Tange HJ, Verhey FRJ, de Vugt ME. A systematic review on the implementation of eHealth interventions for informal caregivers of people with dementia. *Internet Interv*. 2018;13:51-59. doi:10.1016/j.invent.2018.07.002
20. Chudleigh J, Holder P, Moody L, et al. Process evaluation of co-designed interventions to improve communication of positive newborn bloodspot screening results. *BMJ Open*. 2021;11(8):e050773. doi:10.1136/bmjopen-2021-050773
21. Chung J, Demiris G, Thompson HJ. Ethical considerations regarding the use of smart home technologies for older adults: An integrative review. *Annu Rev Nurs Res.* 2016;34(1):155-181. doi:10.1891/0739-6686.34.155
22. Claudel SE, Ceasar JN, Andrews MR, et al. Time to listen: a mixed-method study examining community-based views of mobile technology for interventions to promote physical activity. *BMJ Health Care Inform*. 2020;27(3):e100140. doi:10.1136/bmjhci-2020-100140
23. Collins KM, Armenta RF, Cuevas-Mota J, Liu L, Strathdee SA, Garfein RS. Factors associated with patterns of mobile technology use among persons who inject drugs. *Subst Abus.* 2016;37(4):606-612. doi:10.1080/08897077.2016.1176980
24. Cook EJ, Randhawa G, Sharp C, et al. Exploring the factors that influence the decision to adopt and engage with an integrated assistive telehealth and telecare service in Cambridgeshire, UK: a nested qualitative study of patient “users” and “non-users.” *BMC Health Serv Res*. 2016;16(1):137. doi:10.1186/s12913-016-1379-5
25. Coravos A, Doerr M, Goldsack J, et al. Modernizing and designing evaluation frameworks for connected sensor technologies in medicine. *NPJ Digit Med*. 2020;3(1):37. doi:10.1038/s41746-020-0237-3
26. Cory, N., & Stevens, P. *Building a global framework for digital health services in the era of COVID-19.* Information Technology and Innovation Foundation. 2020.
27. Cresswell, K. Evaluation of implementation of health IT. 206-219.
28. Cresswell K, Williams R, Sheikh A. Developing and applying a formative evaluation framework for health information technology implementations: Qualitative investigation. *J Med Internet Res*. 2020;22(6):e15068. doi:10.2196/15068
29. Cunningham, R. L. Depression screening using smartphone technologies: A data driven approach. 2017.
30. de Korte E, Wiezer N, Bakhuys Roozeboom M, Vink P, Kraaij W. Behavior change techniques in mHealth apps for the mental and physical health of employees: Systematic assessment. *JMIR MHealth UHealth*. 2018;6(10):e167. doi:10.2196/mhealth.6363
31. Doran DM, Haynes RB, Kushniruk A, et al. Supporting evidence-based practice for nurses through information technologies. *Worldviews Evid Based Nurs.* 2010;7(1):4-15. doi:10.1111/j.1741-6787.2009.00179.x
32. Dünnebeil S, Sunyaev A, Blohm I, Leimeister JM, Krcmar H. Determinants of physicians’ technology acceptance for e-health in ambulatory care. *Int J Med Inform.* 2012;81(11):746-760. doi:10.1016/j.ijmedinf.2012.02.002
33. Dyb K, Berntsen GR, Kvam L. Adopt, adapt, or abandon technology-supported person-centred care initiatives: healthcare providers’ beliefs matter. *BMC Health Serv Res.* 2021;21(1):240. doi:10.1186/s12913-021-06262-1
34. Dykes PC, Lowenthal G, Faris A, et al. An implementation science approach to promote optimal implementation, adoption, use, and spread of continuous Clinical Monitoring System Technology. *J Patient Saf.* 2021;17(1):56-62. doi:10.1097/PTS.0000000000000790
35. Eden R, Burton-Jones A, Grant J, Collins R, Staib A, Sullivan C. Digitising an Australian university hospital: qualitative analysis of staff-reported impacts. *Aust Health Rev*. 2020;44(5):677-689. doi:10.1071/AH18218
36. Esmail R, Clement FM, Holroyd-Leduc J, Niven DJ, Hanson HM. Characteristics of knowledge translation theories, models and frameworks for health technology reassessment: expert perspectives through a qualitative exploration. *BMC Health Serv Res.* 2021;21(1):401. doi:10.1186/s12913-021-06382-8
37. Esmail R, Hanson HM, Holroyd-Leduc J, Niven DJ, Clement FM. Identification of knowledge translation theories, models or frameworks suitable for health technology reassessment: a survey of international experts. *BMJ Open.* 2021;11(6):e042251. doi:10.1136/bmjopen-2020-042251
38. Fernando J, Dawson L. The Natural Hospital Environment: a Socio-Technical-Material perspective. *Int J Med Inform.* 2014;83(2):140-158. doi:10.1016/j.ijmedinf.2013.10.008
39. Forsyth JR, Chase H, Roberts NW, Armitage LC, Farmer AJ. Application of the National Institute for health and Care Excellence Evidence Standards Framework for digital health technologies in assessing mobile-delivered technologies for the self-management of type 2 diabetes mellitus: Scoping review. *JMIR Diabetes.* 2021;6(1):e23687. doi:10.2196/23687
40. Fuentes-Caceres, V. *Vendor strategies, EHR capabilities, and patient experience: An analysis in the hospital setting.* Doctoral dissertation, Pennsylvania State University*.* 2016.
41. Gentles SJ, Lokker C, McKibbon KA. Health information technology to facilitate communication involving health care providers, caregivers, and pediatric patients: a scoping review. *J Med Internet Res.* 2010;12(2):e22. doi:10.2196/jmir.1390
42. Gjestsen MT, Wiig S, Testad I. What are the key contextual factors when preparing for successful implementation of assistive living technology in primary elderly care? A case study from Norway. *BMJ Open*. 2017;7(9):e015455. doi:10.1136/bmjopen-2016-015455
43. Glasgow RE, Bull SS, Piette JD, Steiner JF. Interactive behavior change technology. A partial solution to the competing demands of primary care. *Am J Prev Med.* 2004;27(2 Suppl):80-87. doi:10.1016/j.amepre.2004.04.026
44. Godinho MA, Ansari S, Guo GN, Liaw ST. Toolkits for implementing and evaluating digital health: A systematic review of rigor and reporting. *J Am Med Inform Assoc.* 2021;28(6):1298-1307. doi:10.1093/jamia/ocab010
45. Greenhalgh T, Wherton J, Papoutsi C, et al. Analysing the role of complexity in explaining the fortunes of technology programmes: empirical application of the NASSS framework. *BMC Med.* 2018;16(1). doi:10.1186/s12916-018-1050-6
46. Hall A, Wilson CB, Stanmore E, Todd C. Implementing monitoring technologies in care homes for people with dementia: A qualitative exploration using Normalization Process Theory. *Int J Nurs Stud*. 2017;72:60-70. doi:10.1016/j.ijnurstu.2017.04.008
47. Hendrix N, Veenstra DL, Cheng M, Anderson NC, Verguet S. Assessing the economic value of clinical artificial intelligence: Challenges and opportunities. *Value Health*. 2022;25(3):331-339. doi:10.1016/j.jval.2021.08.015
48. Herrmann M, Boehme P, Hansen A, et al. Digital competencies and attitudes toward digital adherence solutions among elderly patients treated with novel anticoagulants: Qualitative study (preprint). *JMIR Preprints*. Published online 2018. doi:10.2196/preprints.13077
49. Hofmann B. Biases and imperatives in handling medical technology. *Health Policy Technol.* 2019;8(4):377-385. doi:10.1016/j.hlpt.2019.10.005
50. Houngbo PT, De Cock Buning T, Bunders J, et al. Ineffective healthcare technology management in Benin’s public health sector: The perceptions of key actors and their ability to address the main problems. *Int J Health Policy Manag*. 2017;6(10):587-600. doi:10.15171/ijhpm.2017.17
51. Inglis SC, Clark RA, Dierckx R, Prieto-Merino D, Cleland JGF. Structured telephone support or non-invasive telemonitoring for patients with heart failure. *Heart*. 2017;103(4):255-257. doi:10.1136/heartjnl-2015-309191
52. Jacob C, Sanchez-Vazquez A, Ivory C. Understanding clinicians’ adoption of mobile health tools: A qualitative review of the most used frameworks. *JMIR MHealth UHealth.* 2020;8(7):e18072. doi:10.2196/18072
53. Junker M, Böhm M, Franz M, Fritsch T, Krcmar H. Value of normative belief in intention to use workplace health promotion apps. *BMC Med Inform Decis Mak*. 2022;22(1):30. doi:10.1186/s12911-022-01760-6
54. Kalayou MH, Endehabtu BF, Tilahun B. The applicability of the modified technology acceptance model (TAM) on the sustainable adoption of eHealth systems in resource-limited settings. *J Multidiscip Healthc*. 2020;13:1827-1837. doi:10.2147/JMDH.S284973
55. Kavandi H, Jaana M. Factors that affect health information technology adoption by seniors: A systematic review. *Health Soc Care Community*. 2020;28(6):1827-1842. doi:10.1111/hsc.13011
56. Kissi J, Dai B, Dogbe CS, Banahene J, Ernest O. Predictive factors of physicians’ satisfaction with telemedicine services acceptance. *Health Informatics J.* 2020;26(3):1866-1880. doi:10.1177/1460458219892162
57. Koivumäki T, Pekkarinen S, Lappi M, Väisänen J, Juntunen J, Pikkarainen M. Consumer adoption of future MyData-based preventive eHealth services: An acceptance model and survey study. *J Med Internet Res*. 2017;19(12):e429. doi:10.2196/jmir.7821
58. Koru G, Alhuwail D, Topaz M, Norcio AF, Mills ME. Investigating the challenges and opportunities in home care to facilitate effective information technology adoption. *J Am Med Dir Assoc*. 2016;17(1):53-58. doi:10.1016/j.jamda.2015.10.008
59. Kuo KM, Liu CF, Ma CC. An investigation of the effect of nurses’ technology readiness on the acceptance of mobile electronic medical record systems. *BMC Med Inform Decis Mak.* 2013;13(1):88. doi:10.1186/1472-6947-13-88
60. Langrial, S., Lehto, T., Oinas-Kukkonen, H., Harjumaa, M., & Karppinen, P. *Native mobile applications for personal well-being: a persuasive systems design evaluation.* 2012.
61. Lau, F., & Kuziemsky, C. *Handbook of eHealth evaluation: an evidence-based approach.* 2016.
62. Lilford RJ, Foster J, Pringle M. Evaluating eHealth: how to make evaluation more methodologically robust. *PLoS Med.* 2009;6(11):e1000186. doi:10.1371/journal.pmed.1000186
63. Lima-Toivanen M, Pereira RM. The contribution of eHealth in closing gaps in primary health care in selected countries of Latin America and the Caribbean. *Rev Panam Salud Publica.* 2018;42:e188. doi:10.26633/RPSP.2018.188
64. Liverpool S, Mota CP, Sales CMD, et al. Engaging children and young people in digital mental health interventions: Systematic review of modes of delivery, facilitators, and barriers. *J Med Internet Res*. 2020;22(6):e16317. doi:10.2196/16317
65. Lyles CR, Adler-Milstein J, Thao C, Lisker S, Nouri S, Sarkar U. Alignment of key stakeholders’ priorities for patient-facing tools in digital health: Mixed methods study. *J Med Internet Res*. 2021;23(8):e24890. doi:10.2196/24890
66. Maunder K, Walton K, Williams P, Ferguson M, Beck E. A framework for eHealth readiness of dietitians. *Int J Med Inform.* 2018;115:43-52. doi:10.1016/j.ijmedinf.2018.04.002
67. May CR, Williams TL, Mair FS, Mort MM, Shaw NT, Gask L. Factors influencing the evaluation of telehealth interventions: preliminary results from a qualitative study of evaluation projects in the UK. *J Telemed Telecare*. 2002;8(2):65-67. doi:10.1258/135763302320302073
68. Moore Z, Angel D, Bjerregaard J, et al. eHealth in Wound Care: from conception to implementation. *J Wound Care*. 2015;24(Sup5):S1-S44. doi:10.12968/jowc.2015.24.Sup5.S1
69. Musselman KE, Shah M, Zariffa J. Rehabilitation technologies and interventions for individuals with spinal cord injury: translational potential of current trends*. J Neuroeng Rehabil.* 2018;15(1). doi:10.1186/s12984-018-0386-7
70. Octavius GS, Antonio F. Antecedents of intention to adopt mobile health (mHealth) application and its impact on intention to recommend: An evidence from Indonesian customers. *Int J Telemed Appl*. 2021;2021:6698627. doi:10.1155/2021/6698627
71. Opoku D, Busse R, Quentin W. Achieving sustainability and scale-up of mobile health noncommunicable disease interventions in sub-Saharan Africa: Views of policy makers in Ghana. *JMIR MHealth UHealth*. 2019;7(5):e11497. doi:10.2196/11497
72. Panda N, Sinyard R, Margo J, et al. Perceptions of mobile health technology in elective surgery: A qualitative study of North American surgeons. *Ann Surg.* 2023;277(3):423-428. doi:10.1097/SLA.0000000000005208
73. Pfadenhauer LM, Mozygemba K, Gerhardus A, et al. Context and implementation: A concept analysis towards conceptual maturity. *Z Evid Fortbild Qual Gesundhwes.* 2015;109(2):103-114. doi:10.1016/j.zefq.2015.01.004
74. Pratt KM, Branch LZ, Houston JB. The practice-Based Implementation (PBI) Network: Technology (Tech) into care pilot. *Transl Behav Med*. 2021;11(1):46-55. doi:10.1093/tbm/ibz174
75. Premkumar, G. A meta-analysis of research on information technology implementation in small business. *J. Organ. Comput. Electron*, 2003;13(2), 91-121.
76. Prgomet M, Georgiou A, Callen J, Westbrook J. Fit between individuals, tasks, technology, and environment (FITTE) framework: A proposed extension of FITT to evaluate and optimise health information technology use. *Stud Health Technol Inform.* 2019;264:744-748. doi:10.3233/SHTI190322
77. Ramsey A, Lord S, Torrey J, Marsch L, Lardiere M. Paving the way to successful implementation: Identifying key barriers to use of technology-based therapeutic tools for behavioral health care. *J Behav Health Serv Res*. 2016;43(1):54-70. doi:10.1007/s11414-014-9436-5
78. Rantanen, T., & Toikko, T. "Employees’ attitudes towards welfare technology in substance abuse treatment in Finland": Corrigendum. 2019.
79. Reardon T. Research findings and strategies for assessing telemedicine costs. *Telemed J E Health*. 2005;11(3):348-369. doi:10.1089/tmj.2005.11.348
80. Reynolds, M. *A digital divide from faculty's perspective: The millennial BSN student meets the baby boomer nursing faculty.* Doctoral dissertation, Capella University. 2017.
81. Roberts CA, Sage AJ, Geryk LL, Sleath BL, Carpenter DM. Adolescent feedback on predisposing, reinforcing and enabling features in asthma self-management apps. *Health Educ J*. 2019;78(7):770-783. doi:10.1177/0017896919836693
82. Saranto K, Kivekäs E, Rosenlund M, et al. How to assess success of HIT project management: An example of the use of the Common Assessment Framework (CAF). *Stud Health Technol Inform.* 2019;264:783-787. doi:10.3233/SHTI190330
83. Schueller SM, Torous J. Scaling evidence-based treatments through digital mental health. *Am Psychol*. 2020;75(8):1093-1104. doi:10.1037/amp0000654
84. Sharma M, Batra K, Flatt J. Testing the multi-theory model (MTM) to predict the use of new technology for social connectedness in the COVID-19 pandemic. *Healthcare (Basel).* 2021;9(7):838. doi:10.3390/healthcare9070838
85. Sittig DF, Singh H. A new sociotechnical model for studying health information technology in complex adaptive healthcare systems. *Qual Saf Health Care*. 2010;19 Suppl 3(Suppl 3):i68-74. doi:10.1136/qshc.2010.042085
86. Szinay D, Perski O, Jones A, Chadborn T, Brown J, Naughton F. Perceptions of factors influencing engagement with health and well-being apps in the United Kingdom: Qualitative interview study. *JMIR MHealth UHealth.* 2021;9(12):e29098. doi:10.2196/29098
87. Tassone C, Keshavjee K, Paglialonga A, Moreira N, Pinto J, Quintana Y. Evaluation of mobile apps for treatment of patients at risk of developing gestational diabetes. *Health Informatics J.* 2020;26(3):1983-1994. doi:10.1177/1460458219896639
88. Tufano, J. T. *Information and communication technologies in patient-centered healthcare redesign: Qualitative studies of provider experience*. University of Washington. 2019.
89. Tuot DS, Liddy C, Vimalananda VG, et al. Evaluating diverse electronic consultation programs with a common framework*. BMC Health Serv Res*. 2018;18(1):814. doi:10.1186/s12913-018-3626-4
90. Ukaga, C. C. *The relationship between information technology and organizational effectiveness as perceived by health care providers*. Doctoral dissertation, Walden University. 2015.
91. Vallespin B, Cornet J, Kotzeva A. Ensuring evidence-based safe and effective mHealth applications. *Stud Health Technol Inform*. 2016;222:248-261.
92. Van Citters AD, Dieni O, Scalia P, et al. Barriers and facilitators to implementing telehealth services during the COVID-19 pandemic: A qualitative analysis of interviews with cystic fibrosis care team members. *J Cyst Fibros*. 2021;20 Suppl 3:23-28. doi:10.1016/j.jcf.2021.09.004
93. van Gemert-Pijnen JEWC, Nijland N, van Limburg M, et al. A holistic framework to improve the uptake and impact of eHealth technologies. *J Med Internet Res.* 2011;13(4):e111. doi:10.2196/jmir.1672
94. Versluis A, van Luenen S, Meijer E, et al. SERIES: eHealth in primary care. Part 4: Addressing the challenges of implementation. *Eur J Gen Pract*. 2020;26(1):140-145. doi:10.1080/13814788.2020.1826431
95. Watt JA, Fahim C, Straus SE, Goodarzi Z. Barriers and facilitators to virtual care in a geriatric medicine clinic: a semi-structured interview study of patient, caregiver and healthcare provider perspectives. *Age Ageing.* 2022;51(1). doi:10.1093/ageing/afab218
96. Waycott J, Scheepers R, Davis H, Howard S, Sonenberg L. The individual in multiple interacting activity systems: IT-supported diabetes management. *Inf Technol People*. 2014;27(4):463-481. doi:10.1108/itp-11-2013-0195
97. Weidner K, Lowman J, Fleischer A, et al. Twitter, telepractice, and the COVID-19 pandemic: A social media content analysis. *Am J Speech Lang Pathol.* 2021;30(6):2561-2571. doi:10.1044/2021_AJSLP-21-00034
98. Weißenfeld MM, Goetz K, Steinhäuser J. Facilitators and barriers for the implementation of telemedicine from a local government point of view - a cross-sectional survey in Germany. *BMC Health Serv Res*. 2021;21(1):919. doi:10.1186/s12913-021-06929-9
99. Whealin JM, Jenchura EC, Wong AC, Zulman DM. How veterans with post-Traumatic Stress Disorder and comorbid health conditions utilize eHealth to manage their health care needs: A mixed-methods analysis. *J Med Internet Res*. 2016;18(10):e280. doi:10.2196/jmir.5594
100. Wienert J, Zeeb H. Implementing health apps for digital Public Health - an implementation science approach adopting the Consolidated Framework for Implementation Research. *Front Public Health.* 2021;9:610237. doi:10.3389/fpubh.2021.610237
101. Wilson EV, Lankton NK. Modeling patients’ acceptance of provider-delivered e-health. *J Am Med Inform Assoc.* 2004;11(4):241-248. doi:10.1197/jamia.M1475
102. Wozney L, Newton AS, Gehring ND, et al. Implementation of eMental Health care: viewpoints from key informants from organizations and agencies with eHealth mandates. *BMC Med Inform Decis Mak*. 2017;17(1). doi:10.1186/s12911-017-0474-9
103. Wright, S. R. Mobile technology adoption: Assessing faculty acceptance using the technology acceptance model. *ProQuest LLC.* 2018.
104. Xie B, Su Z, Zhang W, Cai R.Chinese cardiovascular disease mobile apps’ information types, information quality, and interactive functions for self-management: Systematic review. *JMIR MHealth UHealth.* 2017;5(12):e195. doi:10.2196/mhealth.8549

# 13. Research study in development phase (not in an evaluation or implementation stage) (n=98)

1. Agher D, Sedki K, Despres S, Albinet JP, Jaulent MC, Tsopra R. Encouraging behavior changes and preventing cardiovascular diseases using the Prevent Connect mobile health app: Conception and evaluation of app quality. *J Med Internet Res*. 2022;24(1):e25384. doi:10.2196/25384
2. Alanazi HA. *Exploring policies and strategies for the diffusion of remote patient monitoring (RPM) for the care of senior population*. Order No. 27996936 ed. Portland State University; 2020.
3. Almond H, Cummings E, Turner P. Australia’s personally controlled electronic health record and primary healthcare: generating a framework for implementation and evaluation. *Stud Health Technol Inform*. 2013;188:1-6.
4. Band R, Bradbury K, Morton K, et al. Intervention planning for a digital intervention for self-management of hypertension: a theory-, evidence- and person-based approach. *Implement Sci.* 2017;12(1). doi:10.1186/s13012-017-0553-4
5. Beglaryan M, Petrosyan V, Bunker E. Development of a tripolar model of technology acceptance: Hospital-based physicians’ perspective on EHR. *Int J Med Inform*. 2017;102:50-61. doi:10.1016/j.ijmedinf.2017.02.013
6. Bellandi T, Albolino S. Human Factors and Ergonomics for a Safe Transition to Digital Health. *Stud Health Technol Inform.*2019;265:12-21. doi:https://doi.org/10.3233/SHTI190130
7. Camacho E, Torous J. Introducing an implementation framework for augmenting care with digital technology for early psychosis patients: theory and motivation. *Journal of Mental Health.*2022;31(6):816-824. doi:https://doi.org/10.1080/09638237.2021.1922634
8. Chang WJ, Lo SY, Kuo CL, Wang YL, Hsiao HC. Development of an intervention tool for precision oral self-care: Personalized and evidence-based practice for patients with periodontal disease. *PLoS One.* 2019;14(11):e0225453. doi:10.1371/journal.pone.0225453
9. Chawla D, Thukral A, Kumar P, Deorari A. Harnessing mobile technology to deliver evidence-based maternal-infant care. *Semin Fetal Neonatal Med.* 2021;26(1):101206. doi:10.1016/j.siny.2021.101206
10. Chiu TM, Ku BP. Moderating effects of voluntariness on the actual use of electronic health records for allied health professionals. JMIR Med Inform. 2015;3(1):e7. doi:10.2196/medinform.2548
11. Choo D, Dettman S, Dowell R, Cowan R. Wearable technology to support early child language experiences: What’s important to parents and clinicians? *Stud Health Technol Inform.* 2019;266:51-56. doi:10.3233/SHTI190772
12. Coeurderoy R, Guilmot N, Vas A. Explaining factors affecting technological change adoption: A survival analysis of an information system implementation. *Manag Decis*. 2014;52(6):1082-1100. doi:10.1108/md-10-2013-0540
13. Cooray N, Sun SL, Ho C, et al. Toward a behavior theory-informed and user-centered mobile app for parents to prevent infant falls: Development and usability study. *JMIR Pediatr Parent.* 2021;4(4):e29731. doi:10.2196/29731.
14. Courville KA. *How mobile technology impacts interprofessional team-based care in an acute care setting: A realist perspective.*Order No. 10618343 ed. Texas Woman's University; 2017
15. Cramp DG, Carson ER. A model-based framework for assessing the value of ICT-driven healthcare delivery. *Health Informatics J*. 2001;7(2):90-95. doi:10.1177/146045820100700206
16. Culyer AJ, Bombard Y. An equity framework for health technology assessments. *Med Decis Making*. 2012;32(3):428-441. doi:10.1177/0272989X11426484
17. Daniels J, Fels S, Kushniruk A, Lim J, Ansermino JM. A framework for evaluating usability of clinical monitoring technology. *J Clin Monit Comput*. 2007;21(5):323-330. doi:10.1007/s10877-007-9091-y
18. Dansky KH, Thompson D, Sanner T. A framework for evaluating eHealth research. *Eval Program Plann.* 2006;29(4):397-404. doi:10.1016/j.evalprogplan.2006.08.009
19. Demiris G, Patrick T, Khatri N. Assessing home care agencies’ readiness for telehealth. *AMIA Annu Symp Proc.* Published online 2003:825.
20. Dhagarra D, Goswami M, Kumar G. Impact of trust and privacy concerns on technology acceptance in healthcare: An Indian perspective. *Int J Med Inform.* 2020;141(104164):104164. doi:10.1016/j.ijmedinf.2020.104164
21. Diker A, Cunningham-Sabo L, Bachman K, Stacey JE, Walters LM, Wells L. Effective training design: use of theory and formative assessment: Use of theory and formative assessment. *Health Promot Pract*. 2012;13(4):496-505. doi:10.1177/1524839910386909
22. Ehrler F, Gschwind L, Meyer P, Blondon K. Swiss-meds: An app fostering medication adherence of Swiss patient. *Stud Health Technol Inform.* 2019;259:71-76.
23. Eivazzadeh S, Berglund JS, Larsson TC, Fiedler M, Anderberg P. Most influential qualities in creating satisfaction among the users of health information systems: Study in seven European Union countries. *JMIR Med Inform*. 2018;6(4):e11252. doi:10.2196/11252
24. Escalada-Hernández P, Soto Ruiz N, San Martín-Rodríguez L. Design and evaluation of a prototype of augmented reality applied to medical devices. *Int J Med Inform.* 2019;128:87-92. doi:10.1016/j.ijmedinf.2019.05.004
25. Evans WD, Abroms LC, Poropatich R, Nielsen PE, Wallace JL. Mobile health evaluation methods: the Text4baby case study. *J Health Commun*. 2012;17 Suppl 1(sup1):22-29. doi:10.1080/10810730.2011.649157.
26. Farnia T, Jaulent MC, Marchand G, Yasini M. A collaborative evaluation framework for biometric connected devices in healthcare. *Stud Health Technol Inform.* 2017;245:69-73.
27. Feldman AG, Moore S, Bull S, et al. A smartphone app to increase immunizations in the pediatric solid organ transplant population: Development and initial usability study. *JMIR Form Res*. 2022;6(1):e32273. doi:10.2196/32273
28. Félix IB, Guerreiro MP, Cavaco A, et al. Development of a complex intervention to improve adherence to antidiabetic medication in older people using an anthropomorphic virtual assistant software. *Front Pharmacol*. 2019;10:680. doi:10.3389/fphar.2019.00680
29. Fernemark H, Skagerström J, Seing I, Ericsson C, Nilsen P. Digital consultations in Swedish primary health care: a qualitative study of physicians’ job control, demand and support. *BMC Fam Pract*. 2020;21(1):241. doi:10.1186/s12875-020-01321-8
30. Ford EW, Menachemi N, Phillips MT. Predicting the adoption of electronic health records by physicians: when will health care be paperless? *J Am Med Inform Assoc*. 2006;13(1):106-112. doi:10.1197/jamia.M1913
31. Gallos P, Liaskos J, Georgiadis C, Mechili EA, Mantas J. Measuring the intention of using Augmented Reality technology in the health domain. *Stud Health Technol Inform.* 2019;264:1664-1665. doi:10.3233/SHTI190586
32. Ghozali MT, Satibi S, Ikawati Z, Lazuardi L. Asthma self-management app for Indonesian asthmatics: A patient-centered design. *Comput Methods Programs Biomed.* 2021;211(106392):106392. doi:10.1016/j.cmpb.2021.106392
33. Golant SM. A theoretical model to explain the smart technology adoption behaviors of elder consumers (Elderadopt). J *Aging Stud.* 2017;42:56-73. doi:10.1016/j.jaging.2017.07.003
34. Greenhalgh T, Wherton J, Papoutsi C, et al. Beyond adoption: A new framework for theorizing and evaluating nonadoption, abandonment, and challenges to the scale-up, spread, and sustainability of health and care technologies*. J Med Internet Res.* 2017;19(11):e367. doi:10.2196/jmir.8775.
35. Gregório J, Pizarro Â, Cavaco A, et al. Online pharmaceutical care provision: Full-implementation of an eHealth service using design science research. *Stud Health Technol Inform.* 2015;210:261-265.
36. Harle CA. *Essays on the design and evaluation of information technology-enabled interventions for chronic disease risk assessment and communication.*Order No. 3376478 ed. Carnegie Mellon University; 2009.
37. Herman H, Grobbelaar SS, Pistorius C. The design and development of technology platforms in a developing country healthcare context from an ecosystem perspective. *BMC Med Inform Decis Mak*. 2020;20(1):55. doi:10.1186/s12911-020-1028-0
38. Hicks LL, Boles KE. A comprehensive model for evaluating telemedicine. *Stud Health Technol Inform*. 2004;106:3-13.
39. Huang CY, Yang MC. Empirical investigation of factors influencing consumer intention to use an artificial intelligence-powered mobile application for weight loss and health management. *Telemed J E Health*. 2020;26(10):1240-1251. doi:10.1089/tmj.2019.0182
40. Huser V, Narus SP, Rocha RA. Evaluation of a flowchart-based EHR query system: a case study of RetroGuide. *J Biomed Inform.* 2010;43(1):41-50. doi:10.1016/j.jbi.2009.06.001.
41. Hylock RH, Zeng X. A blockchain framework for patient-centered health records and exchange (HealthChain): Evaluation and proof-of-concept study. *J Med Internet Res.* 2019;21(8):e13592. doi:10.2196/13592
42. Jaana M, Ward MM, Paré G, Sicotte C. Antecedents of clinical information technology sophistication in hospitals. *Health Care Manage Rev*. 2006;31(4):289-299. doi:10.1097/00004010-200610000-00004
43. Jaffar A, Mohd-Sidik S, Foo CN, Admodisastro N, Abdul Salam SN, Ismail ND. Improving pelvic floor muscle training adherence among pregnant women: Validation study. *JMIR Hum Factors*. 2022;9(1):e30989. doi:10.2196/30989
44. Jonnagaddala J, Guo GN, Batongbacal S, Marcelo A, Liaw ST. Adoption of enterprise architecture for healthcare in AeHIN member countries. *BMJ Health Care Inform.* 2020;27(1):e100136. doi:10.1136/bmjhci-2020-100136
45. Kazemi DM, Borsari B, Levine MJ, Lamberson KA, Dooley B. REMIT: Development of a mHealth theory-based intervention to decrease heavy episodic drinking among college students. *Addict Res Theory.* 2018;26(5):377-385. doi:10.1080/16066359.2017.1420783
46. Kennedy M, Kumar R, Ryan NM, Bennett J, La Hera Fuentes G, Gould GS. Codeveloping a multibehavioural mobile phone app to enhance social and emotional well-being and reduce health risks among Aboriginal and Torres Strait Islander women during preconception and pregnancy: a three-phased mixed-methods study. *BMJ Open.* 2021;11(11):e052545. doi:10.1136/bmjopen-2021-052545
47. Kenter W, De Luca V, Illario M, Vollenbroek-Hutten M. “A European developed eHealth technology does not lead to a European implementation strategy towards (business) exploitation: a tale of two countries.” *Int J Integr Care.* 2016;16(5):26. doi:10.5334/ijic.2576
48. Kisekka V, Giboney JS. The effectiveness of health care information technologies: Evaluation of trust, security beliefs, and privacy as determinants of health care outcomes. *J Med Internet Res.* 2018;20(4):e107. doi:10.2196/jmir.9014
49. Kuziemsky CE, Keshavjee K. A framework of ’p’-benefits in health information technology implementation. *Stud Health Technol Inform*. 2015;208:232-236.
50. Leon N, Namadingo H, Bobrow K, et al. Intervention development of a brief messaging intervention for a randomised controlled trial to improve diabetes treatment adherence in sub-Saharan Africa. *BMC Public Health*. 2021;21(1):147. doi:10.1186/s12889-020-10089-6
51. Leon N, Schneider H, Daviaud E. Applying a framework for assessing the health system challenges to scaling up mHealth in South Africa. *BMC Med Inform Decis Mak.* 2012;12(1):123. doi:10.1186/1472-6947-12-123
52. Liebmann EP, Preacher KJ, Richter KP, Cupertino AP, Catley D. Identifying pathways to quitting smoking via telemedicine-delivered care. *Health Psychol.* 2019;38(7):638-647. doi:10.1037/hea0000740
53. Luo Y, Wang G, Li Y, Ye Q. Examining protection motivation and network externality perspective regarding the continued intention to use M-health apps. *Int J Environ Res Public Health.* 2021;18(11):5684. doi:10.3390/ijerph18115684
54. Mardani A, Saraji MK, Mishra AR, Rani P. A novel extended approach under hesitant fuzzy sets to design a framework for assessing the key challenges of digital health interventions adoption during the COVID-19 outbreak. *Appl Soft Comput.* 2020;96(106613):106613. doi:10.1016/j.asoc.2020.106613
55. Mawson S, Nasr N, Parker J, Davies R, Zheng H, Mountain G. A personalized self-management rehabilitation system with an intelligent shoe for stroke survivors: A realist evaluation. *JMIR Rehabil Assist Technol.* 2016;3(1):e1. doi:10.2196/rehab.5079
56. Mburu S, Oboko R. A model for predicting utilization of mHealth interventions in low-resource settings: case of maternal and newborn care in Kenya. *BMC Med Inform Decis Mak*. 2018;18(1). doi:10.1186/s12911-018-0649-z
57. Mehdizadeh H, Asadi F, Emami H, Mehrvar A, Nazemi E. An mHealth self-management system for support children with acute lymphocytic leukemia and their caregivers: Qualitative co-design study. *JMIR Form Res*. 2022;6(4):e36721. doi:10.2196/36721
58. Melin J, Bonn SE, Pendrill L, Trolle Lagerros Y. A questionnaire for assessing user satisfaction with mobile health apps: Development using Rasch measurement theory. *JMIR MHealth UHealth.* 2020;8(5):e15909. doi:10.2196/15909
59. Merrill JA, Deegan M, Wilson RV, Kaushal R, Fredericks K. A system dynamics evaluation model: implementation of health information exchange for public health reporting. *J Am Med Inform Assoc*. 2013;20(e1):e131-8. doi:10.1136/amiajnl-2012-001289
60. Michie S, Hardeman W, Fanshawe T, Prevost AT, Taylor L, Kinmonth AL. Investigating theoretical explanations for behaviour change: the case study of ProActive. *Psychol Health.* 2008;23(1):25-39. doi:10.1080/08870440701670588
61. Mummah SA, Robinson TN, King AC, Gardner CD, Sutton S. IDEAS (integrate, design, assess, and share): A framework and toolkit of strategies for the development of more effective digital interventions to change health behavior. *J Med Internet Res.* 2016;18(12):e317. doi:10.2196/jmir.5927
62. Naccache B, Mesquida L, Raynaud JP, Revet A. Smartphone application for adolescents with anorexia nervosa: an initial acceptability and user experience evaluation. *BMC Psychiatry.* 2021;21(1):467. doi:10.1186/s12888-021-03478-7
63. Nepal S, Li J, Jang-Jaccard J, Alem L. A framework for telehealth program evaluation. *Telemed J E Health*. 2014;20(4):393-404. doi:10.1089/tmj.2013.0093
64. Ngassa Piotie P, Wood P, Webb EM, Hugo JFM, Rheeder P. Designing an integrated, nurse-driven and home-based digital intervention to improve insulin management in under-resourced settings. *Ther Adv Endocrinol Metab*. 2021;12:20420188211054690. doi:10.1177/20420188211054688.
65. Nijland, N. Grounding eHealth: towards a holistic framework for sustainable eHealth technologies. 2011.
66. Odukoya O, Chui MA. Retail pharmacy staff perceptions of design strengths and weaknesses of electronic prescribing. *J Am Med Inform Assoc*. 2012;19(6):1059-1065. doi:10.1136/amiajnl-2011-000779
67. Pires G, Lopes A, Correia P, et al. Usability of a telehealth solution based on TV interaction for the elderly: the VITASENIOR-MT case study. *Univers Access Inf Soc.* Published online 2022:1-12. doi:10.1007/s10209-021-00859-3
68. Polus S, Pfadenhauer L, Brereton L, et al. A consultation guide for assessing the applicability of health technologies: A case study. *Int J Technol Assess Health Care.* 2017;33(5):577-585. doi:10.1017/s0266462317000745
69. Poncette AS, Mosch LK, Stablo L, et al. A remote patient-monitoring system for intensive care medicine: Mixed methods human-centered design and usability evaluation. *JMIR Hum Factors.* 2022;9(1):e30655. doi:10.2196/30655
70. Powell A, Torous J. A patient-centered framework for measuring the economic value of the clinical benefits of digital health apps: Theoretical modeling. *JMIR Ment Health.* 2020;7(10):e18812. doi:10.2196/18812.
71. Richardson J, DePaul V, Officer A, et al. Development and evaluation of self-management and task-oriented approach to rehabilitation training (START) in the home: Case report. *Phys Ther*. 2015;95(6):934-943. doi:10.2522/ptj.20130617
72. Skrabal Ross X, Gunn KM, Patterson P, Olver I. Development of a smartphone program to support adherence to oral chemotherapy in people with cancer. *Patient Prefer Adherence.* 2019;13:2207-2215. doi:10.2147/PPA.S225175
73. Sadegh SS, Khakshour Saadat P, Sepehri MM, Assadi V. A framework for m-health service development and success evaluation. *Int J Med Inform*. 2018;112:123-130. doi:10.1016/j.ijmedinf.2018.01.003
74. Schnall R, Bakken S. Testing the technology acceptance Model: HIV case managers’ intention to use a continuity of care record with context-specific links. *Inform Health Soc Care*. 2011;36(3):161-172. doi:10.3109/17538157.2011.584998
75. Sezgin E, Özkan-Yıldırım S. A cross-sectional investigation of acceptance of health information technology: A nationwide survey of community pharmacists in Turkey. *Res Social Adm Pharm.* 2016;12(6):949-965. doi:10.1016/j.sapharm.2015.12.006
76. Shamu S, Rusakaniko S, Hongoro C. Prioritizing health system and disease burden factors: an evaluation of the net benefit of transferring health technology interventions to different districts in Zimbabwe. *Clinicoecon Outcomes Res*. 2016;8:695-705. doi:10.2147/CEOR.S95037
77. Sharifian R, Askarian F, Nematolahi M, Farhadi P. Factors influencing nurses’ acceptance of hospital information systems in Iran: application of the Unified Theory of Acceptance and Use of Technology. *Health Inf Manag*. 2014;43(3):23-28. doi:10.1177/183335831404300303
78. Shen N, Strauss J, Silver M, Carter-Langford A, Wiljer D. The eHealth trust Model: A patient privacy research framework. *Stud Health Technol Inform*. 2019;257:382-387.
79. Sicotte C, Paré G, Moreault MP, Paccioni A. A risk assessment of two interorganizational clinical information systems. J Am Med Inform Assoc. 2006;13(5):557-566. doi:10.1197/jamia.M2012
80. Sockolow PS, Crawford PR, Lehmann HP. Health services research evaluation principles. Broadening a general framework for evaluating health information technology. *Methods Inf Med*. 2012;51(2):122-130. doi:10.3414/ME10-01-0066
81. Sonney J, Cho EE, Zheng Q, Kientz JA. Refinement of a parent-child shared asthma management mobile health app: Human-centered design study. *JMIR Pediatr Parent*. 2022;5(1):e34117. doi:10.2196/34117
82. Stoldt JP, Price M, Weber J. Towards a clinical analytics adoption maturity framework for primary care. *Stud Health Technol Inform.* 2019;257:399-403.
83. Taherdoost H. Development of an adoption model to assess user acceptance of e-service technology: E-service technology acceptance model. *Behav Inf Technol*. 2018;37(2):173-197. doi:10.1080/0144929x.2018.1427793
84. Tarzia L, May C, Hegarty K. Assessing the feasibility of a web-based domestic violence intervention using chronic disease frameworks: reducing the burden of “treatment” and promoting capacity for action in women abused by a partner*. BMC Womens Health*. 2016;16(1):73. doi:10.1186/s12905-016-0352-0
85. Tarzia L, Murray E, Humphreys C, et al. I-DECIDE: An online intervention drawing on the Psychosocial Readiness Model for women experiencing domestic violence. *Womens Health Issues.* 2016;26(2):208-216. doi:10.1016/j.whi.2015.07.011
86. Tavares J, Oliveira T. Electronic Health Record Portal Adoption: a cross country analysis. *BMC Med Inform Decis Mak*. 2017;17(1). doi:10.1186/s12911-017-0482-9
87. Kowatsch T, Otto L, Harperink S, Cotti A, Schlieter H. A design and evaluation framework for digital health interventions. It - Inf Technol. 2019;61(5-6):253-263. doi:10.1515/itit-2019-0019
88. Tonkin E, Jeffs L, Wycherley TP, et al. A smartphone app to reduce sugar-sweetened beverage consumption among young adults in Australian remote Indigenous communities: Design, formative evaluation and user-testing. *JMIR MHealth UHealth*. 2017;5(12):e192. doi:10.2196/mhealth.8651
89. Tung FC, Chang SC, Chou CM. An extension of trust and TAM model with IDT in the adoption of the electronic logistics information system in HIS in the medical industry. *Int J Med Inform*. 2008;77(5):324-335. doi:10.1016/j.ijmedinf.2007.06.006
90. Vamos CA, Green SM, Griner S, et al. Identifying implementation science characteristics for a prenatal oral health eHealth application. *Health Promot Pract.* 2020;21(2):246-258. doi:10.1177/1524839918793628
91. Walters LEM, Scott RE, Mars M. A teledermatology scale-up framework and roadmap for sustainable scaling: Evidence-based development. *J Med Internet Res.* 2018;20(6):e224. doi:10.2196/jmir.9940
92. Wildenbos GA, Jaspers MWM, Schijven MP, Dusseljee-Peute LW. Mobile health for older adult patients: Using an aging barriers framework to classify usability problems. *Int J Med Inform.* 2019;124:68-77. doi:10.1016/j.ijmedinf.2019.01.006
93. Williams PA, Lovelock B, Cabarrus T, Harvey M. Improving digital hospital transformation: Development of an outcomes-based Infrastructure Maturity Assessment framework. *JMIR Med Inform.* 2019;7(1):e12465. doi:10.2196/12465
94. Xin M, Viswanath K, Li AYC, et al. The effectiveness of electronic health interventions for promoting HIV-preventive behaviors among men who have sex with men: Meta-analysis based on an integrative framework of design and implementation features. *J Med Internet Res*. 2020;22(5):e15977. doi:10.2196/15977
95. Yusof MM. A Socio-technical and Lean approach towards a framework for health information systems-induced error. *Stud Health Technol Inform*. 2019;257:508-512.
96. Zervogianni V, Fletcher-Watson S, Herrera G, et al. A framework of evidence-based practice for digital support, co-developed with and for the autism community. *Autism*. 2020;24(6):1411-1422. doi:10.1177/1362361319898331
97. Zheng K. *Design, implementation, user acceptance, and evaluation of a clinical decision support system for evidence-based medicine practice.* 2007.

# Not a digital health intervention (DHI) (n=64)

1. Al Rabayah AA, Al Froukh RF, Sawalha RD. A capacity-building programme in health technology assessment for hospital pharmacists in a low- to middle-income country. *J Pharm Health Serv Res*. 2018;9(3):275-280. doi:10.1111/jphs.12241
2. Alaiad A, Alsharo M, Alnsour Y. The determinants of M-health adoption in developing countries: An empirical investigation. *Appl Clin Inform.* 2019;10(5):820-840. doi:10.1055/s-0039-1697906
3. Aleluia IRS, Medina MG, Almeida PF de, Vilasbôas ALQ. Care coordination in primary health care: an evaluative study in a municipality in the Northeast of Brazil. *Cien Saude Colet.* 2017;22(6):1845-1856. doi:10.1590/1413-81232017226.02042017
4. Ammenwerth E. Technology acceptance models in health informatics: TAM and UTAUT. *Stud Health Technol Inform*. 2019;263:64-71. doi:10.3233/SHTI190111
5. Anton MT, Jones DJ. Adoption of technology‐enhanced treatments: Conceptual and practical considerations. *Clin Psychol (New Yor*k). 2017;24(3):223-240. doi:10.1111/cpsp.12197
6. Benis A, Tamburis O, Chronaki C, Moen A. One Digital Health: A unified framework for future health ecosystems. *J Med Internet Res*. 2021;23(2):e22189. doi:10.2196/22189
7. Bernstein E, Topp D, Shaw E, et al. A preliminary report of knowledge translation: lessons from taking screening and brief intervention techniques from the research setting into regional systems of care. *Acad Emerg Med*. 2009;16(11):1225-1233. doi:10.1111/j.1553-2712.2009.00516.x
8. Bikson, KL. *Understanding innovation in end-of-life care: A comparative case study of hospital-based palliative care programs*. Order No. 3299557 ed. University of California, Los Angeles; 2007
9. Bowen JM, Patterson LL, O’Reilly D, et al. Conditionally funded field evaluations and practical trial design within a health technology assessment framework. *J Am Coll Radiol*. 2009;6(5):324-331. doi:10.1016/j.jacr.2009.01.023
10. Camacho E, Hoffman L, Lagan S, et al. Technology Evaluation and Assessment Criteria for health apps (TEACH-apps): Pilot study. *J Med Internet Res*. 2020;22(8):e18346. doi:10.2196/18346
11. Chen B, Sivo S, Seilhamer R, Sugar A, Mao J. User acceptance of mobile technology: A campus-wide implementation of blackboard's mobile™ learn application. *Journal of Educational Computing Research*. 2013;49(3):327-343. doi:10.2190/ec.49.3.c
12. Čudanov M, Jaško O. Adoption of information and communication technologies and dominant management orientation in organisations. *Behav Inf Technol.* 2012;31(5):509-523. doi:10.1080/0144929x.2010.499520
13. De Paula Rogerio, A. The construction of usefulness: How users and context create meaning with a social networking system. 2004.
14. Dev S, Fawcett J, Ahmad S, Wu WC, Schwenke D. Implementation of early follow-up care after heart failure hospitalization. *Am J Manag Care*. 2021;27(2):e42-e47. doi:10.37765/ajmc.2021.88588
15. Dhas BN, Samuel PS, Manigandan C. Use of computer access technology as an alternative to writing for a pre-school child with athetoid cerebral palsy—A case report. *Occupational Therapy In Health Care.* 2014;28(3):318-332. doi:10.3109/07380577.2013.874063
16. Dimopoulos-Bick T, Clowes KE, Conciatore K, Haertsch M, Verma R, Levesque JF. Barriers and facilitators to implementing playlists as a novel personalised music intervention in public healthcare settings in New South Wales, Australia. *Aust J Prim Health.* 2019;25(1):31-36. doi:10.1071/PY18084
17. Dong BJ, Williams MR, Bingham JT, Tokumoto J, Allen JD. Outcome of challenging HIV case consultations provided via teleconference by the Clinician Consultation Center to the Federal Bureau of Prisons. *J Am Pharm Assoc (2003).* 2017;57(4):516-519. doi:10.1016/j.japh.2017.05.002
18. Ehlers L, Vestergaard M, Kidholm K, et al. Doing mini-health technology assessments in hospitals: a new concept of decision support in health care? *Int J Technol Assess Health Care.* 2006;22(3):295-301. doi:10.1017/s02664623060511785.
19. Erolin C, Reid L, McDougall S. Using virtual reality to complement and enhance anatomy education. *J Vis Commun Med*. 2019;42(3):93-101. doi:10.1080/17453054.2019.1597626
20. Faija CL, Connell J, Welsh C, et al. What influences practitioners’ readiness to deliver psychological interventions by telephone? A qualitative study of behaviour change using the Theoretical Domains Framework. *BMC Psychiatry.* 2020;20(1):371. doi:10.1186/s12888-020-02761-3
21. Federici S, Scherer MJ, Borsci S. An ideal model of an assistive technology assessment and delivery process. *Technol Disabil*. 2014;26(1):27-38. doi:10.3233/tad-140402
22. Feng W, Tu R, Lu T, Zhou Z. Understanding forced adoption of self-service technology: the impacts of users’ psychological reactance. *Behav Inf Technol*. 2019;38(8):820-832. doi:10.1080/0144929x.2018.1557745
23. Fredriksson JJ, Mazzocato P, Muhammed R, Savage C. Business model framework applications in health care: A systematic review. *Health Serv Manage Res*. 2017;30(4):219-226. doi:10.1177/0951484817726918
24. Furness PN. Introducing new health interventions: diagnostics is the Cinderella of health technology assessment. *BMJ*. 2006;332(7535):235-236. doi:10.1136/bmj.332.7535.235-b
25. Gagnon MP, Sánchez E, Pons JMV. Integration of health technology assessment recommendations into organizational and clinical practice: A case study in Catalonia. *Int J Technol Assess Health Care.* 2006;22(2):169-176. doi:10.1017/S0266462306050987
26. Gartshore E, Briggs L, Blake H. Development and evaluation of an educational training package to promote health and wellbeing. *Br J Nurs*. 2017;26(21):1182-1186. doi:10.12968/bjon.2017.26.21.1182
27. Greenberg, ME. *The process of care delivery in telephone nursing practice: a grounded theory approach.* Order No. 3178848 ed. The University of Arizona; 2005.
28. Grimm SE, Pouwels X, Ramaekers BLT, et al. Implementation barriers to value of information analysis in health technology decision making: Results from a process evaluation. *Value Health*. 2021;24(8):1126-1136. doi:10.1016/j.jval.2021.03.013
29. Hebron, I. *Technology acceptance factors affecting adoption of wireless data technology*. Order No. 3338093 ed. Lynn University; 2008.
30. Huic M, Tandara Hacek R, Svajger I. Health technology assessment in central, eastern, and south European countries: Croatia. *Int J Technol Assess Health Care*. 2017;33(3):376-383. doi:10.1017/s026646231700054x
31. Jack SM, Munro-Kramer ML, Williams JR, et al. Recognising and responding to intimate partner violence using telehealth: Practical guidance for nurses and midwives. *J Clin Nurs.* 2021;30(3-4):588-602. doi:10.1111/jocn.15554
32. Jake-Schoffman DE, Silfee VJ, Waring ME, et al. Methods for evaluating the content, usability, and efficacy of commercial mobile health apps. *JMIR MHealth UHealth.* 2017;5(12):e190. doi:10.2196/mhealth.8758
33. Kovarik AJ. *Implementing sustainable safer sex education on a college campus.* Order No. 3683175 ed. State University of New York at Binghamton; 2012.
34. Leone C, Lim JSL, Stern A, Charles J, Black S, Baecker R. Communication technology adoption among older adult veterans: the interplay of social and cognitive factors. *Aging Ment Health.* 2018;22(12):1666-1677. doi:10.1080/13607863.2017.1381946
35. Mauco KL, Scott RE, Mars M. Critical analysis of e-health readiness assessment frameworks: suitability for application in developing countries. *J Telemed Telecare*. 2018;24(2):110-117. doi:10.1177/1357633X16686548
36. McIntosh S, Pérez-Ramos J, Demment MM, et al. Development and implementation of culturally tailored offline mobile health surveys. *JMIR Public Health Surveill.* 2016;2(1):e28. doi:10.2196/publichealth.5408
37. Morrow E, Robert G, Maben J, Griffiths P. Implementing large-scale quality improvement: lessons from The Productive Ward: Releasing Time to Care: Lessons from The Productive Ward: Releasing Time to CareTM. *Int J Health Care Qual Assur.* 2012;25(4):237-253. doi:10.1108/09526861211221464
38. Muroff J, Robinson W. Tools of engagement: Practical considerations for utilizing technology-based tools in CBT practice. *Cogn Behav Pract.* 2022;29(1):81-96. doi:10.1016/j.cbpra.2020.01.004
39. Nastasi BK, Varjas K, Schensul SL, Silva KT, Schensul JJ, Ratnayake P. The Participatory Intervention Model: A framework for conceptualizing and promoting intervention acceptability. *Sch Psychol Q*. 2000;15(2):207-232. doi:10.1037/h0088785
40. Nelson C, Madiba S. Barriers to the implementation of the ward-based outreach team program in Mpumalanga Province: Results from process evaluation. *J Prim Care Community Health.* 2020;11:2150132720975552. doi:10.1177/2150132720975552
41. O’Connor S. Using social media to engage nurses in health policy development. *J Nurs Manag*. 2017;25(8):632-639. doi:10.1111/jonm.12501
42. Ølholm AM, Kidholm K, Birk-Olsen M, Christensen JB. Hospital managers’ need for information on health technology investments. *Int J Technol Assess Health Care.* 2015;31(6):414-425. doi:10.1017/S0266462315000665
43. Park C, Kim DG, Cho S, Han HJ. Adoption of multimedia technology for learning and gender difference. *Comput Human Behav.* 2019;92:288-296. doi:10.1016/j.chb.2018.11.029
44. Purao S, Storey VC. Evaluating the adoption potential of design science efforts: The case of APSARA. *Decis Support Syst*. 2008;44(2):369-381. doi:10.1016/j.dss.2007.04.007
45. Qureshi O, Endale T, Ryan G, et al. Barriers and drivers to service delivery in global mental health projects. *Int J Ment Health Syst*. 2021;15(1):14. doi:10.1186/s13033-020-00427-x
46. Shah F, Sells JR, Werthman J, Abraham C, Ali AM, Callaway-Lane C. A multi-site evaluation of A national employee wellness initiative at the Department of Veterans Affairs. *Glob Adv Health Med*. 2022;11:21649561211053804. doi:10.1177/21649561211053805
47. Shahidi N, Tossan V, Cacho-Elizondo S. Assessment of a mobile educational coaching app: Exploring adoption patterns and barriers in France. I*nternational Journal of Technology and Human Interaction (IJTHI).* 2018;14(1):22-43. doi:https://doi.org/10.4018/IJTHI.2018010102
48. Simons M, Van De Ven M, Coupé V, et al. Early technology assessment of using whole genome sequencing in personalized oncology. *Expert Rev Pharmacoecon Outcomes Res*. 2021;21(3):343-351. doi:10.1080/14737167.2021.1917386
49. Stacey D, Bakker D, Ballantyne B, et al. Managing symptoms during cancer treatments: evaluating the implementation of evidence-informed remote support protocols*. Implement Sci.* 2012;7(1):110. doi:10.1186/1748-5908-7-110
50. Tam G, Chan EYY, Liu S. A Web-based course on Public Health Principles in Disaster and Medical Humanitarian Response: Survey among students and faculty. *JMIR Med Educ.* 2018;4(1):e2. doi:10.2196/mededu.8495
51. Thompson EL, Fulda KG, Grace J, Galvin AM, Spence EE. The implementation of an interpersonal violence screening program in primary care settings: Lessons learned. *Health Promot Pract.* 2022;23(4):640-649. doi:10.1177/1524839921989273
52. Lee TT. Evaluation of health information technology - key elements in the framework. *J Nurs Res.* 2016;24(4):283-285. doi:10.1097/jnr.0000000000000192
53. Tung FC, Chang SC. Nursing students’ behavioral intention to use online courses: a questionnaire survey. *Int J Nurs Stud.* 2008;45(9):1299-1309. doi:10.1016/j.ijnurstu.2007.09.011
54. Ulucanlar S, Faulkner A, Peirce S, Elwyn G. Technology identity: the role of sociotechnical representations in the adoption of medical devices. *Soc Sci Med.* 2013;98:95-105. doi:10.1016/j.socscimed.2013.09.008
55. van Haeften-van Dijk AM, van Weert JCM, Dröes RM. Implementing living room theatre activities for people with dementia on nursing home wards: a process evaluation study. *Aging Ment Health.* 2015;19(6):536-547. doi:10.1080/13607863.2014.955459
56. Vargo-Warran J. *A quantitative study of nursing faculty's personal and professional use of technology.* Order No. 10155693 ed. University of Phoenix; 2016.
57. Vishwanath A, Chen H. Technology clusters: Using multidimensional scaling to evaluate and structure technology clusters. *J Am Soc Inf Sci Technol*. 2006;57(11):1451-1460. doi:10.1002/asi.20435
58. Vrkljan B. Facilitating technology use in older adulthood: The Person-Environment-Occupation Model revisited. *Br J Occup Ther*. 2010;73(9):396-404. doi:10.4276/030802210x12839367526011
59. Waite-Jones JM, Majeed-Ariss R, Smith J, Stones SR, Van Rooyen V, Swallow V. Young people’s, parents’, and professionals’ views on required components of mobile apps to support self-management of juvenile arthritis: Qualitative study. *JMIR MHealth UHealth.* 2018;6(1):e25. doi:10.2196/mhealth.9179
60. Wilkinson SA, Hughes E, Moir J, Jobber C, Ackerie A. Process of knowledge translation within routine clinical care: Implementing best practice in weight management: Implementing a weight management model of care. *Nutr Diet.* 2018;75(4):363-371. doi:10.1111/1747-0080.12469
61. Woo MY, Frank JR, Lee AC. Point-of-care ultrasonography adoption in Canada: using diffusion theory and the Evaluation Tool for Ultrasound skills Development and Education (ETUDE). *CJEM.* 2014;16(05):345-351. doi:10.2310/8000.2013.131243
62. Wu LH, Wu LC, Chang SC. Exploring consumers’ intention to accept smartwatch. *Comput Human Behav.* 2016;64:383-392. doi:10.1016/j.chb.2016.07.005
63. Yano EM. The role of organizational research in implementing evidence-based practice: QUERI Series. *Implement Sci.* 2008;3(1):29. doi:10.1186/1748-5908-3-29
64. Zucca C, Long E, Hilton J, McCann M. Appraising the implementation of complexity approaches within the public health sector in Scotland. An assessment framework for pre-implementation policy evaluation. *Front Public Health*. 2021;9:653588. doi:10.3389/fpubh.2021.653588

# 5. Systematic/Scoping review with no relevant included articles (n=57)

1. Abbott PA, Foster J, Marin H de F, Dykes PC. Complexity and the science of implementation in health IT--knowledge gaps and future visions*. Int J Med Inform*. 2014;83(7):e12-22. doi:10.1016/j.ijmedinf.2013.10.009

2. Ahmed T, Lucas H, Khan AS, Islam R, Bhuiya A, Iqbal M. eHealth and mHealth initiatives in Bangladesh: a scoping study. *BMC Health Serv Res*. 2014;14(1):260. doi:10.1186/1472-6963-14-260

3. Al-Durra M, Torio MB, Cafazzo JA. The use of behavior change theory in internet-based asthma self-management interventions: a systematic review. *J Med Internet Res.* 2015;17(4):e89. doi:10.2196/jmir.4110

4. Al-Qirim NAY. Critical success factors for strategic telemedicine planning in New Zealand. *Telemed J E Health*. 2005;11(5):600-607. doi:10.1089/tmj.2005.11.600

5. Alshahrani A, Stewart D, MacLure K. A systematic review of the adoption and acceptance of eHealth in Saudi Arabia: Views of multiple stakeholders. *Int J Med Inform.* 2019;128:7-17. doi:10.1016/j.ijmedinf.2019.05.007

6. Apolinário-Hagen J, Kemper J, Stürmer C. Public acceptability of E-mental health treatment services for psychological problems: A scoping review. *JMIR Ment Health.* 2017;4(2):e10. doi:10.2196/mental.6186

7. Benyakorn S, Riley SJ, Calub CA, Schweitzer JB. Current state and model for development of technology-based care for attention deficit hyperactivity disorder. *Telemed J E Health.* 2016;22(9):761-768. doi:10.1089/tmj.2015.0169

8. Bhattarai P, Phillips JL. The role of digital health technologies in management of pain in older people: An integrative review. *Arch Gerontol Geriatr.* 2017;68:14-24. doi:10.1016/j.archger.2016.08.008

9. Bidonde, J., et al. (2017). FreeStyle Libre flash glucose self‐monitoring system: a single‐technology assessment. Oslo, Norway, Knowledge Centre for the Health Services at The Norwegian Institute of Public Health.

10. Bonten TN, Rauwerdink A, Wyatt JC, et al. Online guide for electronic health evaluation approaches: Systematic scoping review and concept mapping study. *J Med Internet Res*. 2020;22(8):e17774. doi:10.2196/17774

11. Borum C. Barriers for hospital-based nurse practitioners utilizing clinical decision support systems: A systematic review. *Comput Inform Nurs.* 2018;36(4):177-182. doi:10.1097/CIN.0000000000000413

12. Boydell KM, Hodgins M, Pignatiello A, Teshima J, Edwards H, Willis D. Using technology to deliver mental health services to children and youth: a scoping review. *J Can Acad Child Adolesc Psychiatry.* 2014;23(2):87-99.

13. Brørs G, Pettersen TR, Hansen TB, et al. Modes of e-Health delivery in secondary prevention programmes for patients with coronary artery disease: a systematic review. *BMC Health Serv Res.* 2019;19(1):364. doi:10.1186/s12913-019-4106-1

14. Bucci S, Schwannauer M, Berry N. The digital revolution and its impact on mental health care. *Psychol Psychother*. 2019;92(2):277-297. doi:10.1111/papt.12222

15. Cajita MI, Gleason KT, Han HR. A systematic review of mHealth-based Heart Failure interventions. *J Cardiovasc Nurs*. 2016;31(3):E10-22. doi:10.1097/JCN.0000000000000305

16. Carter J, Sandall J, Shennan AH, Tribe RM. Mobile phone apps for clinical decision support in pregnancy: a scoping review. *BMC Med Inform Decis Mak*. 2019;19(1):219. doi:10.1186/s12911-019-0954-1

17. Cheewakriangkrai C, Kietpeerakool C, Charoenkwan K, et al. Health education interventions to promote early presentation and referral for women with symptoms of endometrial cancer. *Cochrane Database Syst Rev.* 2020;3:CD013253. doi:10.1002/14651858.CD013253.pub2

18. Chib A, van Velthoven MH, Car J. mHealth adoption in low-resource environments: a review of the use of mobile healthcare in developing countries. *J Health Commun.* 2015;20(1):4-34. doi:10.1080/10810730.2013.864735

19. Cruz-Martínez RR, Wentzel J, Asbjørnsen RA, et al. Supporting self-management of cardiovascular diseases through remote monitoring technologies: Metaethnography review of frameworks, models, and theories used in research and development. *J Med Internet Res*. 2020;22(5):e16157. doi:10.2196/16157

20. Dandachi D, Lee C, Morgan RO, Tavakoli-Tabasi S, Giordano TP, Rodriguez-Barradas MC. Integration of telehealth services in the healthcare system: with emphasis on the experience of patients living with HIV. *J Investig Med.* 2019;67(5):815-820. doi:10.1136/jim-2018-000872

21. Drabble SJ, O’Cathain A, Scott AJ, et al. Mechanisms of action of a web-based intervention with health professional support to increase adherence to nebulizer treatments in adults with cystic fibrosis: Qualitative interview study. *J Med Internet Res.* 2020;22(10):e16782. doi:10.2196/16782

22. Enam A, Torres-Bonilla J, Eriksson H. Evidence-based evaluation of eHealth interventions: Systematic literature review. *J Med Internet Res*. 2018;20(11):e10971. doi:10.2196/10971

23. Fanta G, Pretorius L, & Erasmus LD. An evaluation of eHealth systems implementation frameworks for sustainability in resource constrained environments: A literature review. 2015.

24. Froehlich EH. Technology. Acceptance predictability and the complexities of nursing. *J Nurs Inform.* 2019;4(2):21-23.

25. Grigorovich A, Kulandaivelu Y, Newman K, et al. Factors affecting the implementation, use, and adoption of real-time location system technology for persons living with cognitive disabilities in long-term care homes: Systematic review. *J Med Internet Res*. 2021;23(1):e22831. doi:10.2196/22831

26. Harris J, Felix L, Miners A, et al. Adaptive e-learning to improve dietary behaviour: a systematic review and cost-effectiveness analysis. *Health Technol Assess*. 2011;15(37):1-160. doi:10.3310/hta15370

27. Harst L, Lantzsch H, Scheibe M. Theories predicting end-user acceptance of telemedicine Use: Systematic review. *J Med Internet Res.* 2019;21(5):e13117. doi:10.2196/13117

28. Helleman J, Kruitwagen ET, van den Berg LH, Visser-Meily JMA, Beelen A. The current use of telehealth in ALS care and the barriers to and facilitators of implementation: a systematic review. *Amyotroph Lateral Scler Frontotemporal Degener*. 2020;21(3-4):167-182. doi:10.1080/21678421.2019.1706581

29. Hersh W, Totten A, Eden K, et al. Health Information Exchange. *Evid Rep Technol Assess (Full Rep).* 2015;(220):1-465. doi:10.23970/AHRQEPCERTA220

30. Horvath KJ, Lammert S, LeGrand S, Muessig KE, Bauermeister JA. Using technology to assess and intervene with illicit drug-using persons at risk for HIV. *Curr Opin HIV AIDS*. 2017;12(5):458-466. doi:10.1097/COH.0000000000000398

31. Hoxhaj I, Govaerts L, Simoens S, et al. A systematic review of the value assessment frameworks used within health technology assessment of omics technologies and their actual adoption from HTA agencies. *Int J Environ Res Public Health*. 2020;17(21):8001. doi:10.3390/ijerph17218001

32. Huang Y, Benford S, Blake H. Digital interventions to reduce sedentary behaviors of office workers: Scoping review. *J Med Internet Res*. 2019;21(2):e11079. doi:10.2196/11079

33. Joshi NK, Bhardwaj P, Suthar P, Jain YK, Joshi V, Manda B. Assessment of monitoring and online payment system (Asha Soft) in Rajasthan using benefit evaluation (BE) framework. *J Family Med Prim Care*. 2020;9(5):2405-2410. doi:10.4103/jfmpc.jfmpc_48_20

34. Jun S, Plint AC, Campbell SM, Curtis S, Sabir K, Newton AS. Point-of-care cognitive support technology in emergency departments: A scoping review of technology acceptance by clinicians. *Acad Emerg Med.* 2018;25(5):494-507. doi:10.1111/acem.13325

35. Klaic M, Galea MP. Using the technology acceptance model to identify factors that predict likelihood to adopt Tele-neurorehabilitation. Front Neurol. 2020;11:580832. doi:10.3389/fneur.2020.580832

36. Leung K, Lu-McLean D, Kuziemsky C, et al. Using patient and family engagement strategies to improve outcomes of health information technology initiatives: Scoping review. *J Med Internet Res.* 2019;21(10):e14683. doi:10.2196/14683

37. Lindquist AM, Johansson PE, Petersson GI, Saveman BI, Nilsson GC. The use of the Personal Digital Assistant (PDA) among personnel and students in health care: a review. *J Med Internet Res*. 2008;10(4):e31. doi:10.2196/jmir.1038

38. Matthew-Maich N, Harris L, Ploeg J, et al. Designing, implementing, and evaluating mobile health technologies for managing chronic conditions in older adults: A scoping review. *JMIR MHealth UHealth.* 2016;4(2):e29. doi:10.2196/mhealth.5127

39. McIntosh JRD. Do E-health interventions improve physical activity in young people: A systematic review. *J Community Med Health Educ*. 2017;07(05). doi:10.4172/2161-0711-c1-030

40. Messiah SE, Sacher PM, Yudkin J, et al. Application and effectiveness of eHealth strategies for metabolic and bariatric surgery patients: A systematic review. *Digit Health.* 2020;6:2055207619898987. doi:10.1177/2055207619898987

41. Moehead A, DeSouza K, Walsh K, Pit SW. A Web-based dementia education program and its application to an Australian Web-based Dementia Care Competency and Training Network: Integrative systematic review. *J Med Internet Res.* 2020;22(1):e16808. doi:10.2196/16808

42. Moore G, Wilding H, Gray K, Castle D. Participatory methods to engage health service users in the development of electronic health resources: Systematic review. *J Particip Med*. 2019;11(1):e11474. doi:10.2196/11474

43. Moshi MR, Tooher R, Merlin T. Suitability of current evaluation frameworks for use in the health technology assessment of mobile medical applications: A systematic review. *Int J Technol Assess Health Care*. 2018;34(5):464-475. doi:10.1017/S026646231800051X

44. Müller AM, Alley S, Schoeppe S, Vandelanotte C. The effectiveness of e-& mHealth interventions to promote physical activity and healthy diets in developing countries: A systematic review. *Int J Behav Nutr Phys Act*. 2016;13(1):109. doi:10.1186/s12966-016-0434-2

45. Niknejad N, Ismail W, Bahari M, Nazari B. Understanding telerehabilitation technology to evaluate stakeholders’ adoption of telerehabilitation services: A systematic literature review and directions for further research*. Arch Phys Med Rehabil.* 2021;102(7):1390-1403. doi:10.1016/j.apmr.2020.12.014

46. Ooi CY, Ng CJ, Sales AE, Lim HM. Implementation strategies for web-based apps for screening: Scoping review. *J Med Internet Res*. 2020;22(7):e15591. doi:10.2196/15591

47. Peek STM, Wouters EJM, van Hoof J, Luijkx KG, Boeije HR, Vrijhoef HJM. Factors influencing acceptance of technology for aging in place: a systematic review. *Int J Med Inform.* 2014;83(4):235-248. doi:10.1016/j.ijmedinf.2014.01.004

48. Peng BW, Schoech D. Grounding online prevention interventions in theory: Guidelines from a review of selected theories and research. *J Technol Hum Serv*. 2008;26(2-4):376-396. doi:10.1080/15228830802097315

49. Ross J, Stevenson F, Lau R, Murray E. Factors that influence the implementation of e-health: a systematic review of systematic reviews (an update). *Implement Sci.* 2016;11(1):146. doi:10.1186/s13012-016-0510-7

50. Sanchez D, Reiner JF, Sadlon R, Price OA, Long MW. Systematic review of school telehealth evaluations. *J Sch Nurs.* 2019;35(1):61-76. doi:10.1177/1059840518817870

51. Shen N, Yufe S, Saadatfard O, Sockalingam S, Wiljer D. Rebooting kirkpatrick: Integrating information system theory into the evaluation of web-based continuing professional development interventions for interprofessional education. *J Contin Educ Health Prof.* 2017;37(2):137-146. doi:10.1097/CEH.0000000000000154

52. Tilahun B, Gashu KD, Mekonnen ZA, Endehabtu BF, Angaw DA. Mapping the role of digital health technologies in the case detection, management, and treatment outcomes of neglected tropical diseases: a scoping review. *Trop Med Health.* 2021;49(1):17. doi:10.1186/s41182-021-00307-1

53. van den Bosch SC, van de Voort NEM, Xi T, Kool RB, Bergé SJ, Faber MJ. Oral & Maxillofacial surgery is ready for patient-centred eHealth interventions - the outcomes of a scoping review. *Int J Oral Maxillofac Surg*. 2019;48(6):830-840. doi:10.1016/j.ijom.2018.10.001

54. Varabyova Y, Blankart CR, Greer AL, Schreyögg J. The determinants of medical technology adoption in different decisional systems: A systematic literature review. *Health Policy.* 2017;121(3):230-242. doi:10.1016/j.healthpol.2017.01.005

55. Varsi C, Solberg Nes L, Kristjansdottir OB, et al. Implementation strategies to enhance the implementation of eHealth programs for patients with chronic illnesses: Realist systematic review. *J Med Internet Res*. 2019;21(9):e14255. Published 2019 Sep 27. doi:10.2196/14255

56. Wozney L, Huguet A, Bennett K, et al. How do eHealth programs for adolescents with depression work? A realist review of persuasive system design components in internet-based psychological therapies. *J Med Internet Res*. 2017;19(8):e266. doi:10.2196/jmir.7573

57. Wozney L, McGrath PJ, Gehring ND, et al. EMental healthcare technologies for anxiety and depression in childhood and adolescence: Systematic review of studies reporting implementation outcomes. *JMIR Ment Health*. 2018;5(2):e48. doi:10.2196/mental.9655

# 6. Study protocol (insufficient data) (n=50)

1. Implementing affordable technology to improve mobility and physical activity in an inpatient rehabilitation setting; a feasibility study
2. An investigation of the efficacy of an eHealth nutrition intervention on lifestyle-related colorectal cancer risk in patients undergoing colonoscopy
3. Agarwal S, Glenton C, Tamrat T, et al. Decision-support tools via mobile devices to improve quality of care in primary healthcare settings. *Cochrane Database Syst Rev.* 2021;7(7):CD012944. doi:10.1002/14651858.CD012944.pub2
4. Ahmadvand A, Drennan J, Burgess J, et al. Novel augmented reality solution for improving health literacy around antihypertensives in people living with type 2 diabetes mellitus: protocol of a technology evaluation study. *BMJ Open*. 2018;8(4):e019422. doi:10.1136/bmjopen-2017-019422
5. Allan S, Mcleod H, Bradstreet S, et al. Understanding implementation of a digital self-monitoring intervention for relapse prevention in psychosis: Protocol for a mixed method process evaluation (preprint). *JMIR Preprints*. Published online 2019. doi:10.2196/preprints.15634
6. Anton MT, Ridings LE, Hanson R, et al. Hybrid type 1 randomized controlled trial of a tablet-based application to improve quality of care in child mental health treatment. *Contemp Clin Trials.* 2020;94(106010):106010. doi:10.1016/j.cct.2020.106010
7. Bele S, Cassidy C, Curran J, Johnson DW, Saunders C, Bailey JAM. Barriers and enablers to implementing a virtual tertiary-regional Telemedicine Rounding and Consultation (TRAC) model of inpatient pediatric care using the Theoretical Domains Framework (TDF) approach: a study protocol. *BMC Health Serv Res*. 2019;19(1):29. doi:10.1186/s12913-018-3859-2
8. Bowden JL, Egerton T, Hinman RS, et al. Protocol for the process and feasibility evaluations of a new model of primary care service delivery for managing pain and function in patients with knee osteoarthritis (PARTNER) using a mixed methods approach*. BMJ Open*. 2020;10(2):e034526. doi:10.1136/bmjopen-2019-034526
9. Bruno E, Biondi A, Böttcher S, et al. Remote Assessment of Disease and Relapse in epilepsy: Protocol for a multicenter prospective cohort study. *JMIR Res Protoc*. 2020;9(12):e21840. doi:10.2196/21840
10. Cheng KKF, Siah RCJ, Ream E, Kanesvaran R, Armes J. Protocol for a scoping review of digital health for older adults with cancer and their families. BMJ Open. 2020;10(8):e038876. doi:10.1136/bmjopen-2020-038876
11. Chu KH, Escobar-Viera CG, Matheny SJ, Davis EM, Primack BA. Tobacco cessation mobile app intervention (Just Kwit! study): protocol for a pilot randomized controlled pragmatic trial. *Trials*. 2019;20(1):147. doi:10.1186/s13063-019-3246-2
12. Ciani O, Cucciniello M, Petracca F, et al. Lung Cancer App (LuCApp) study protocol: a randomised controlled trial to evaluate a mobile supportive care app for patients with metastatic lung cancer. *BMJ Open*. 2019;9(2):e025483. doi:10.1136/bmjopen-2018-025483
13. Collins R. Nurses’ perceived usefulness of secure texting applications for the purpose of patient care. *Online Journal of Nursing Informatics.* 2019;23(1).
14. de Zwaan M, Herpertz S, Zipfel S, et al. INTERBED: internet-based guided self-help for overweight and obese patients with full or subsyndromal binge eating disorder. A multicenter randomized controlled trial. *Trials*. 2012;13(1):220. doi:10.1186/1745-6215-13-220
15. Dontje ML, Kruitwagen-van Reenen E, Visser-Meily JMA, Beelen A, Study Group ALS Home-monitoring and Coaching. Implementation and evaluation of an e-health innovation for personalized care for patients with amyotrophic lateral sclerosis (ALS): protocol for a participatory action research study. *Implement Sci Commun*. 2021;2(1):25. doi:10.1186/s43058-021-00130-z
16. Eivazzadeh S, Anderberg P, Larsson TC, Fricker SA, Berglund J. Evaluating health information systems using ontologies. *JMIR Med Inform*. 2016;4(2):e20. doi:10.2196/medinform.5185
17. Evans EH, Araújo-Soares V, Adamson A, et al. The NULevel trial of a scalable, technology-assisted weight loss maintenance intervention for obese adults after clinically significant weight loss: study protocol for a randomised controlled trial. *Trials.* 2015;16(1):421. doi:10.1186/s13063-015-0931-7
18. Ezendam NP, Oenema A, van de Looij-Jansen PM, Brug J. Design and evaluation protocol of "FATaintPHAT", a computer-tailored intervention to prevent excessive weight gain in adolescents. *BMC Public Health*. 2007;7:324. doi:10.1186/1471-2458-7-324
19. Gulliver A, Banfield M, Reynolds J, Miller S, Galati C, Morse AR. A peer-led electronic mental health recovery app in an adult mental health service: Study protocol for a pilot trial. *JMIR Res Protoc*. 2017;6(12):e248. doi:10.2196/resprot.8795
20. Kalkhoran S, Appelle NA, Napoles AM, et al. Beyond the ask and advise: Implementation of a computer tablet intervention to enhance provider adherence to the 5As for smoking cessation. *J Subst Abuse Treat*. 2016;60:91-100. doi:10.1016/j.jsat.2015.05.009
21. Kernebeck S, Holle D, Pogscheba P, et al. A tablet app- and sensor-based assistive Technology intervention for informal caregivers to manage the challenging behavior of people with dementia (the insideDEM study): Protocol for a feasibility study. *JMIR Res Protoc*. 2019;8(2):e11630. doi:10.2196/11630
22. Krishnasamy C, Ong SY, Yock Y, Lim I, Rees R, Car J. Factors influencing the implementation, adoption, use, sustainability and scalability of mLearning for medical and nursing education: a systematic review protocol. *Syst Rev.* 2016;5(1):178. doi:10.1186/s13643-016-0354-x
23. Leung MM, Maeo KF, Verdaguer S, Wyka K. Testing a Web-based interactive comic tool to decrease obesity risk among minority preadolescents: Protocol for a pilot randomized control trial. *JMIR Res Protoc*. 2018;7(11):e10682. doi:10.2196/10682
24. Lewis M, Palmer VJ, Kotevski A, et al. Rapid design and delivery of an experience-based co-designed mobile app to support the mental health needs of health care workers affected by the COVID-19 pandemic: Impact evaluation protocol. *JMIR Res Protoc*. 2021;10(3):e26168. doi:10.2196/26168
25. Luo L, King AA, Carroll Y, et al. Electronic health record-embedded individualized pain plans for emergency department treatment of Vaso-occlusive episodes in adults with sickle cell disease: Protocol for a preimplementation and postimplementation study. *JMIR Res Protoc*. 2021;10(4):e24818. doi:10.2196/24818
26. Margolis KL, Crain AL, Bergdall AR, et al. Design of a pragmatic cluster-randomized trial comparing telehealth care and best practice clinic-based care for uncontrolled high blood pressure. *Contemp Clin Trials*. 2020;92(105939):105939. doi:10.1016/j.cct.2020.105939
27. Miao M, Power E, Rietdijk R, Brunner M, Debono D, Togher L. A web-based service delivery model for communication training after brain injury: Protocol for a mixed methods, prospective, hybrid type 2 implementation-effectiveness study. *JMIR Res Protoc.* 2021;10(12):e31995. doi:10.2196/31995
28. Kernel Networks Inc. Program using a mobile application versus telephone advice on patients at risk of coronary heart disease : A pilot RCT. *Case Medical Research.* Published online 2019. doi:10.31525/ct1-nct04054258
29. Kernel Networks Inc. Testing of a patient-centered E-health implementation model in addiction treatment. *Case Medical Research*. Published online 2019. doi:10.31525/ct1-nct03954184
30. Kernel Networks Inc. Optimizing acute post-operative dental pain management using new health information technology. *Case Medical Research*. Published online 2019. doi:10.31525/ct1-nct03881891
31. Kernel Networks Inc. Interactive obesity treatment approach (iOTA) for obesity prevention in adults with early serious mental illness: iOTA-SMI. *Case Medical Research.* Published online 2019. doi:10.31525/ct1-nct03980743
32. VA Office of Research and Development. Hybrid Effectiveness-Implementation Study to Improve Clopidogrel Adherence. 2012.
33. Wong E. Program Using a Mobile Application Versus Telephone Advice on Patients at Risk of Coronary Heart Disease : a Pilot RCT. The Hong Kong Polytechnic University. 2019.
34. University NI. Treatment of Depression Using a Mobile Application in the Dominican Republic. 2021.
35. Pal T, Hull PC, Koyama T, et al. Enhancing Cancer care of rural dwellers through telehealth and engagement (ENCORE): protocol to evaluate effectiveness of a multi-level telehealth-based intervention to improve rural cancer care delivery. *BMC Cancer.* 2021;21(1):1262. doi:10.1186/s12885-021-08949-4
36. Parry D, Carter P, Koziol-McLain J, Feather J. A model for usability evaluation for the development and implementation of consumer eHealth interventions. *Stud Health Technol Inform.* 2015;216:968.
37. Philis-Tsimikas A, Fortmann AL, Godino JG, et al. Dulce Digital-Me: Protocol for a randomized controlled trial of an adaptive mHealth intervention for underserved Hispanics with diabetes. *Trials*. 2022;23(1):80. doi:10.1186/s13063-021-05899-x
38. Popp CJ, St-Jules DE, Hu L, et al. The rationale and design of the personal diet study, a randomized clinical trial evaluating a personalized approach to weight loss in individuals with pre-diabetes and early-stage type 2 diabetes. *Contemp Clin Trials.* 2019;79:80-88. doi:10.1016/j.cct.2019.03.001
39. Quanbeck AR, Gustafson DH, Marsch LA, et al. Integrating addiction treatment into primary care using mobile health technology: protocol for an implementation research study. *Implement Sci.* 2014;9(1). doi:10.1186/1748-5908-9-65
40. Rasmussen CDN, Svendsen MJ, Wood K, et al. App-delivered self-management intervention trial selfBACK for people with low back pain: Protocol for implementation and process evaluation. *JMIR Res Protoc*. 2020;9(10):e20308. doi:10.2196/20308
41. Raut A, Mustafayev R, Srinivasan R, et al. Hybrid type 1 effectiveness/implementation trial of the international Guide for Monitoring Child Development: protocol for a cluster-randomised controlled trial. *BMJ Paediatr Open*. 2021;5(1):e001254. doi:10.1136/bmjpo-2021-001254
42. Riley WT. Theoretical models to inform technology-based health behavior interventions. In: *Behavioral Health Care and Technology*. Oxford University Press; 2014:13-24.
43. Scherrenberg M, Storms V, van der Velde AE, et al. A home hospitalisation strategy for patients with an acute episode of heart failure using a digital health-supported platform: A multicentre feasibility study - A rationale and study design. *Cardiology.* 2021;146(6):793-800. doi:10.1159/000519085
44. Snyder ME, Chewning B, Kreling D, et al. An evaluation of the spread and scale of PatientToc™ from primary care to community pharmacy practice for the collection of patient-reported outcomes: A study protocol. *Res Social Adm Pharm*. 2021;17(2):466-474. doi:10.1016/j.sapharm.2020.03.019
45. Soobiah C, Cooper M, Kishimoto V, et al. Identifying optimal frameworks to implement or evaluate digital health interventions: a scoping review protocol. *BMJ Open.* 2020;10(8):e037643. doi:10.1136/bmjopen-2020-037643
46. Steele GC, Tang T, Armas A, et al. Building a Digital Bridge to support patient-centered care transitions from hospital to home for older adults with complex care needs: Protocol for a co-design, implementation, and evaluation study. *JMIR Res Protoc.* 2020;9(11):e20220. doi:10.2196/20220
47. Tilahun B, Smillie K, Bardosh KL, et al. Identifying barriers and facilitators of 13 mHealth projects in North America and Africa: Protocol for a 5-year implementation science study. *JMIR Res Protoc*. 2018;7(7):e162. doi:10.2196/resprot.9633
48. Walsh E, Sahm LJ, Kearney PM, et al. The PHARMS (Patient Held Active Record of Medication Status) feasibility study: a research proposal. *BMC Res Notes.* 2018;11(1):6. doi:10.1186/s13104-017-3118-3
49. Ware P, Ross HJ, Cafazzo JA, Laporte A, Seto E. Implementation and evaluation of a smartphone-based telemonitoring program for patients with heart failure: Mixed-methods study protocol. *JMIR Res Protoc.* 2018;7(5):e121. doi:10.2196/resprot.9911
50. White VM, Molfenter T, Gustafson DH, et al. NIATx-TI versus typical product training on e-health technology implementation: a clustered randomized controlled trial study protocol. *Implement Sci*. 2020;15(1):94. doi:10.1186/s13012-020-01053-4

# Clinical trial (insufficient data) (n=25)

1. Andersson G. A Guided Internet-delivered Individually-tailored ACT-influenced CBT Intervention to Improve Psychosocial Outcomes in Breast Cancer (INNOVBC). 2018.

2. Assistance Publique Hôpitaux de Paris. Prevention in a Virtual Community Game (SCOTT). 2020.

3. Barber E. Prehabilitation During the Neoadjuvant Window of Opportunity in Older Women With Ovarian Cancer (FIT4SURGERY). 2021.

4. Bartels SL. Digital Behavioural Treatment for Chronic Pain (DAHLIA). 2021.

5. Breitenstein SM, Schoeny M, Risser H, Johnson T. A study protocol testing the implementation, efficacy, and cost effectiveness of the ezParent program in pediatric primary care. Contemporary clinical trials. 2016;50:229-37. PMID: CN-01211509. doi: <https://doi.org/10.1016/j.cct.2016.08.017>.

6. Clark J. TransPrEP: social Network-Based PrEP Adherence for Transgender Women in Peru (TransPrEP). 2017.

7. Davis J. Mobile Mindfulness for Alcohol Use and PTSD Among Veterans. 2023.

8. Fernández Lao C. Pain Treatment in a Breast Cancer Population. PaiNEd Study. (PaiNEd). 2022.

9. Fox Chase Cancer Center. Mychoice: Testing an Interactive mHealth Tool. 2018.

10. Iversen T. Pragmatic Randomized Control Trial of Telehealth vs Standard Care in Follow-up of Patients With Chronic Conditions. 2019.

11. Kaiser Permanente. CV Wizard: does a Clinical Decision Support Tool Improve CVD Risk Factor Control in Safety Net Clinics? (CV_WIZARD). 2018.

12. Lorna P. WEB-Based Physiotherapy for People With Axial Spondyloarthritis. 2015.

13. Mazza D, Chakraborty S, Camões-Costa V, Kenardy J, Brijnath B, Mortimer D, et al. Implementing work-related Mental health guidelines in general PRacticE (IMPRovE): a trial evaluating the impact of a complex intervention involving a digital Community of Practice and academic detailing on guideline adherence. 2020. PMID: CN-02184575.

14. McClelland S. Motivation Matters Study. 2016.

15. Miller S. Internet-Based Motivational Interviewing for Colonoscopy. 2017.

16. Murray M. WelTelOAKTREE: text Messaging to Support Patients With HIV/AIDS in British Columbia (WelTelOAKTREE). 2013.

17. Sievanen H. eHealth Intervention on Physical Activity for Type 2 Diabetics - Frustrated by COVID-19. 2019.

18. Swendeman D. mHealth to Enhance & Sustain Drug Use Reduction of the QUIT BI in Primary Care (QUIT-Mobile). 2021.

19. Temple University. Partnering With WIC to Prevent Excessive Weight Gain in Pregnancy. 2018.

20. University of Alberta. Telemonitoring of Hypertensive Patients With Chronic Kidney Disease. 2022.

21. VA Office of Research and Development. TelemEdiciNe-bAsed Cognitive TherapY for Migraines. 2020.

22. VA Office of Research and Development. LINK-HF2 - Remote Monitoring Analytics in Heart Failure. 2021.

23. VA Office of Research and Development. Developing an Online Therapeutic Intervention for Chronic Pain in Veterans. 2022.

24. Women's College Hospital. Evaluate the Value of Telehomecare for Diabetes. 2016.

25. Yale University. Project SMART Automated Pillbox Study. https://ClinicalTrials.gov/show/NCT04418076; 2017.

# 3. Unable to locate full text (n=12)

1. Bowen E, Walker K. A Framework for Intervention Development. In: *The Psychology of Violence in Adolescent Romantic Relationships.* Palgrave Macmillan UK; 2015:123-142.

2. Brennan PF, Moore SM, Bjornsdottir G, Jones J, Visovsky C, Rogers M. HeartCare: an Internet-based information and support system for patient home recovery after coronary artery bypass graft (CABG) surgery. *J Adv Nurs.* 2001;35(5):699-708. doi:10.1046/j.1365-2648.2001.01902.x

3. Hersh W, Totten A, Eden K, et al. Health Information Exchange. *Evid Rep Technol Assess (Full Rep).* 2015;(220):1-465. doi:10.23970/AHRQEPCERTA220

4. Claes J, Cornelissen E, McDermott C, et al. (#) PATHway-I: Feasibility and preliminary efficacy of a technology-enabled home-based cardiac rehabilitation system. *Acta Cardiologica*. 2019;74, 58-58.

5. Hersh W, Totten A, Eden K, et al. Health Information Exchange. *Evid Rep Technol Assess (Full Rep).* 2015;(220):1-465. doi:10.23970/AHRQEPCERTA220

6. Mathur A, Kvedar JC, Watson AJ. Connected health: a new framework for evaluation of communication technology use in care improvement strategies for type 2 diabetes. *Curr Diabetes Rev.* 2007;3(4):229-234. doi:10.2174/1573399077823300038

7. Rantanen T, Toikko T. Employees' attitudes towards welfare technology in substance abuse treatment in Finland [published correction appears in Nordisk Alkohol Nark. 2019 Dec;36(6):569]. *Nordisk Alkohol Nark*. 2017;34(2):131-144. doi:10.1177/1455072517691060

8. Rasmussen LV, Connolly JJ, Del Fiol G, et al. Infobuttons for genomic medicine: Requirements and barriers. *Appl Clin Inform*. 2021;12(2):383-390. doi:10.1055/s-0041-1729164

9. Scal P, Garwick A, & Horvath KJ. Making rheumtogrow: the rationale and framework for an internet based health care transition intervention. In Transition from pediatric to adult medical care. *Nova Science Publishers Inc*. 2012:107-119

10. Stanimirovic, A. Digital heath interventions in mental health. *Research Anthology on Mental Health Stigma, Education, and Treatment.* 2021:328-340. doi:10.4018/978-1-7998-8544-3.ch019.

11. Tsu VD, Free MJ. Using technology to reduce maternal mortality in low-resource settings: challenges and opportunities. *J Am Med Womens Assoc*. 2002;57(3):149-153.

12. Walker R. *Factors that influence consumers' adoption intention of mobile health services in the United States: A quantitative study.* Order No. 10744366 ed. Northcentral University; 2017.

# Conference abstract (insufficient data) (n=9)

1. Agar M, Chenoweth L, Mitchell G, Goodall S, Beattie E, Luscombe G, Pond D, Phillips J, Luckett T, Davidson P. Implementing facilitated case conferencing for aged care residents with advanced dementia-development of a palliative care planning coordinator role. *Palliat. Med.* 2016;(9). doi: 10.117/0269216316646056

2. Cockle-Hearne, J., Barnett, D., White, I., Hicks, J., Gage, H., & Faithfull, S. (2016, October). Online self-management for distress after prostate cancer treatment: assessing acceptability and viability of The Getting Down to Coping (R) Programme. In *PSYCHO-ONCOLOGY*. 2016;25:110-110.

3. Eisenstein EL, Lobach DF, Montgomery P, Kawamoto K, Anstrom KJ. Evaluating implementation fidelity in health information technology interventions. *AMIA Annu Symp Proc*. Published online 2007:211-215.

4. Hogan-Murphy D, Tonna AP, Stewart D, Strath A, Cunningham, S. Key stakeholders' perceptions, experiences and vision towards the implementation of electronic systems for medicines in the hospital setting: a theory-based qualitative study. *Int J Pharm.* 2016;24(11).

5. Kadu MM, Steele Gray C, Berta W. Assessing factors that influence the implementation of technologies enabling integrated care delivery for older adults with complex needs: A systematic review. *Int J Integr Care.* 2018;18(s2):374. doi:10.5334/ijic.s2374

6. Liaw ST, Ansari S, Jonnagaddala J, et al. Use of mHealth for promoting healthy ageing and supporting delivery of age-friendly care services: a systematic review. Int J Integr Care. 2019;19(4):147. doi:10.5334/ijic.s3147

7. Lin J, Ahsan MD, Badiner N, et al. Web-based tools for family cancer history and cancer risk modeling: what are the barriers to successful implementation? *Gynecol Oncol.* 2021;162:S333. doi:10.1016/s0090-8258(21)01282-8

8. Obayashi K, Kodate N, Kondo H, Okamoto Y, Ishii Y, Masuyama S. 69 How useful is an integrated assistive technology system in a nursing home? Understanding its effectiveness on users and their perceptions. *Age Ageing.* 2019;48(Supplement_3):iii1-iii16. doi:10.1093/ageing/afz102.15

9. Organised by: RRD (Netherlands), Chair persons: Stephanie Jansen-Kosterink - Netherlands, Marian Hurmuz - Netherlands. 26.C. Workshop: How to evaluate digital health: three examples based on the model of Continuous eHealth Evaluation. *Eur J Public Health.* 2020;30(Supplement_5). doi:10.1093/eurpub/ckaa165.1233

# Duplicate (n=6)

1. Aref-Adib G, McCloud T, Ross J, et al. Factors affecting implementation of digital health interventions for people with psychosis or bipolar disorder, and their family and friends: a systematic review. *Lancet Psychiatry*. 2019;6(3):257-266. doi:10.1016/S2215-0366(18)30302-X

3. Dansky KH, Thompson D, Sanner T. A framework for evaluating eHealth research. *Eval Program Plann*. 2006;29(4):397-404. doi:10.1016/j.evalprogplan.2006.08.009

4. Kurki M, Anttila M, Koivunen M, Marttunen M, Välimäki M. Nurses’ experiences of the use of an Internet-based support system for adolescents with depressive disorders. *Inform Health Soc Care.* 2017;43(3):1-14. doi:10.1080/17538157.2016.1269110

5. Lewis M, Palmer VJ, Kotevski A, et al. Rapid design and delivery of an experience-based co-designed mobile app to support the mental health needs of health care workers affected by the COVID-19 pandemic: Impact evaluation protocol. *JMIR Res Protoc*. 2021;10(3):e26168. doi:10.2196/26168

6. Peels DA, van Stralen MM, Bolman C, et al. Development of web-based computer-tailored advice to promote physical activity among people older than 50 years. *J Med Internet Res*. 2012;14(2):e39. doi:10.2196/jmir.1742

7. Prgomet M, Georgiou A, Callen J, Westbrook J. Fit between individuals, tasks, technology, and environment (FITTE) framework: A proposed extension of FITT to evaluate and optimise health information technology use*. Stud Health Technol Inform.* 2019;264:744-748. doi:10.3233/SHTI190322

# Not in English or in French (n=6)

1. Baum-Cohen I, Barzilay S, Shenhod-Malhi E, Weiss PL. Applying a SWOT analysis in assessing technology adoption in cccupational therapy. 2015.

2. Braun E, Hawle M, & Wild C. Social implications of the increasing spread of technology in medicine (Structured abstract).

3. Chang, CP. The technology acceptance model and its application in a telehealth program for the elderly with chronic illnesses. *Hu Li Za Zhi.* 2015;62(3), 11.

5. Li L, Liu Q, Wang H, Tang XJ, Lei X, Wang J, Song WF, Wang YA, et al. feasibility study on short messaging service (SMS) as a strategy to improve adherence to TB services. *Chinese Journal of Evidence-Based Medicine*. 2011;11(6), 631-635.

6. Monteagudo Peña JL, Salvador CH, García-López F. Methodology for the implementation of e-Health services for chronic patient monitoring and control. *Rev Esp Salud Publica*. 2004;78(5):571-581. doi:10.1590/s1135-57272004000500002

# Commentary (insufficient data) (n=5)

1. Al-Qirim NAY. Teledermatology: the case of adoption and diffusion of telemedicine health Waikato in New Zealand. *Telemed J E Health*. 2003;9(2):167-177. doi:10.1089/153056203766437507

2. Balas EA, Chapman WW. Road map for diffusion of innovation in health care. *Health Aff (Millwood*). 2018;37(2):198-204. doi:10.1377/hlthaff.2017.1155

3. Bouttell J, Briggs A, Hawkins N. The thorny issue of value alignment: how development-focused health technology assessment can help find win-win situations for patients and healthcare systems and commercial investors. *Int J Technol Assess Health Care.* 2021;37(1):e57. doi:10.1017/S026646232100026X

4. Egnell, P. Northern Sweden's framework for innovation in ehealth. Br. J. Health Care Manag., 2015;22(10), 16-18.2005.

5. Lo C, Yu J, Görges M, Matava C. Anesthesia in the modern world of apps and technology: Implications and impact on wellness. *Paediatr Anaesth*. 2021;31(1):31-38. doi:10.1111/pan.14051

# Letter to editor (insufficient data) (n=2)

1. Chalkidou A. A different animal but the same beast? Using development-focused health technology assessment to define the value proposition of medical technologies. *Int J Technol Assess Health Care*. 2021;37(1):e58. doi:10.1017/S0266462321000209

2. Mukherjee K. Relevance of the newly defined Health Technology Assessment: COVID-19 and beyond. *Int J Technol Assess Health Care.* 2021;37(1):e44. doi:10.1017/S0266462321000192
