# Supplementary material for: Mapping Theories, Models, and Frameworks to Evaluate Digital Health Interventions: Scoping Review
Source: J Med Internet Res. 2024 Feb 5;26:e51098. doi: 10.2196/51098 (PMC10877497; doi:10.2196/51098)
Supplement: Multimedia Appendix 4 [file jmir_v26i1e51098_app4.docx]

**Multimedia Appendix 4.** List of included studies.

[1-156]

1. Abimbola S, Patel B, Peiris D, Patel A, Harris M, Usherwood T, et al. The NASSS framework for ex post theorisation of technology-supported change in healthcare: worked example of the TORPEDO programme. BMC Medicine. 2019 Dec 30;17(1):233. PMID: 31888718. doi: 10.1186/s12916-019-1463-x.

2. Aboelmaged M, Hashem G. RFID application in patient and medical asset operations management: A technology, organizational and environmental (TOE) perspective into key enablers and impediments. International Journal of Medical Informatics. 2018;118:58-64. PMID: 131430379. . doi: 10.1016/j.ijmedinf.2018.07.009.

3. Adeoye-Olatunde OA, Curran GM, Jaynes HA, Hillman LA, Sangasubana N, Chewning BA, et al. Preparing for the spread of patient-reported outcome (PRO) data collection from primary care to community pharmacy: a mixed-methods study. Implement Sci Commun. 2022 Mar 14;3(1):29. PMID: 35287764. doi: 10.1186/s43058-022-00277-3.

4. Akdur G, Aydin MN, Akdur G. Adoption of Mobile Health Apps in Dietetic Practice: Case Study of Diyetkolik. JMIR MHealth and UHealth. 2020 10 02;8(10):e16911. PMID: 33006566. doi: https://dx.doi.org/10.2196/16911.

5. Alvarado N, McVey L, Elshehaly M, Greenhalgh J, Dowding D, Ruddle R, et al. Analysis of a Web-Based Dashboard to Support the Use of National Audit Data in Quality Improvement: Realist Evaluation. Journal of medical Internet research. 2021;23(11):e28854. doi: https://dx.doi.org/10.2196/28854.

6. Bandini A, Kalsi-Ryan S, Craven BC, Zariffa J, Hitzig SL. Perspectives and recommendations of individuals with tetraplegia regarding wearable cameras for monitoring hand function at home: Insights from a community-based study. J Spinal Cord Med. 2021;44(sup1):S173-S84. PMID: 33960874. doi: 10.1080/10790268.2021.1920787.

7. Bardosh KL, Murray M, Khaemba AM, Smillie K, Lester R. Operationalizing mHealth to improve patient care: a qualitative implementation science evaluation of the WelTel texting intervention in Canada and Kenya. Global Health. 2017 Dec 6;13(1):87. PMID: 29208026. doi: 10.1186/s12992-017-0311-z.

8. Barker LT, Bond WF, Vincent AL, Cooley KL, McGarvey JS, Vozenilek JA, et al. A novel in situ simulation framework for introduction of a new technology: the 3-Act-3-Debrief model. Adv Simul (Lond). 2020;5:25. PMID: 32999737. doi: 10.1186/s41077-020-00145-x.

9. Bastos de Carvalho A, Lee Ware S, Belcher T, Mehmeti F, Higgins EB, Sprang R, et al. Evaluation of multi-level barriers and facilitators in a large diabetic retinopathy screening program in federally qualified health centers: a qualitative study. Implement Sci Commun. 2021 May 22;2(1):54. PMID: 34022946. doi: 10.1186/s43058-021-00157-2.

10. Batsis JA, McClure AC, Weintraub AB, Sette D, Rotenberg S, Stevens CJ, et al. Barriers and facilitators in implementing a pilot, pragmatic, telemedicine-delivered healthy lifestyle program for obesity management in a rural, academic obesity clinic. Implement Sci Commun. 2020;1:83. PMID: 33015640. doi: 10.1186/s43058-020-00075-9.

11. Beukes EW, Manchaiah V, Baguley DM, Allen PM, Andersson G. Process evaluation of Internet-based cognitive behavioural therapy for adults with tinnitus in the context of a randomised control trial. Int J Audiol. 2018 Feb;57(2):98-109. PMID: 28990807. doi: 10.1080/14992027.2017.1384858.

12. Black WE, Esposito-Smythers C, Liu FF, Leichtweis R, Peterson AP, Fagan C. Leveraging Health Information Technology to Meet The Joint Commission's Standard for Measurement-Based Care: A Case Study. Jt Comm J Qual Patient Saf. 2020 Jun;46(6):353-8. PMID: 32371060. doi: 10.1016/j.jcjq.2020.03.006.

13. Boet S, Etherington C, Lam S, Le M, Proulx L, Britton M, et al. Implementation of the Operating Room Black Box Research Program at the Ottawa Hospital Through Patient, Clinical, and Organizational Engagement: Case Study. J Med Internet Res. 2021 Mar 16;23(3):e15443. PMID: 33724199. doi: 10.2196/15443.

14. Brunet N, Moore DT, Lendvai Wischik D, Mattocks KM, Rosen MI. Increasing buprenorphine access for veterans with opioid use disorder in rural clinics using telemedicine. Subst Abus. 2022;43(1):39-46. PMID: 32078492. doi: 10.1080/08897077.2020.1728466.

15. Busse TS, Kernebeck S, Dreier LA, Meyer D, Zenz D, Haas P, et al. Planning for Implementation Success of an Electronic Cross-Facility Health Record for Pediatric Palliative Care Using the Consolidated Framework for Implementation Research (CFIR). Int J Environ Res Public Health. 2022 Jan 1;19(1). PMID: 35010713. doi: 10.3390/ijerph19010453.

16. Cady RG. Measuring the Impact of Technology on Nurse Workflow: A Mixed Methods Approach. 2012 (Ph.D.):283 -.

17. Carper MM, McHugh RK, Barlow DH. The dissemination of computer-based psychological treatment: a preliminary analysis of patient and clinician perceptions. Adm Policy Ment Health. 2013 Mar;40(2):87-95. PMID: 22001968. doi: 10.1007/s10488-011-0377-5.

18. Catchpole K, Privette A, Roberts L, Alfred M, Carter B, Woltz E, et al. A Smartphone Application for Teamwork and Communication in Trauma: Pilot Evaluation "in the Wild". Hum Factors. 2021 Jun 14:187208211021717. PMID: 34126795. doi: https://dx.doi.org/10.1177/00187208211021717.

19. Chaves RO, de Oliveira PAV, Rocha LC, David JPF, Ferreira SC, Santos A, et al. An Innovative Streaming Video System With a Point-of-View Head Camera Transmission of Surgeries to Smartphones and Tablets: An Educational Utility. Surg Innov. 2017 Oct;24(5):462-70. PMID: 28639871. doi: 10.1177/1553350617715162.

20. Chib A. The Aceh Besar midwives with mobile phones project: Design and evaluation perspectives using the information and communication technologies for healthcare development model. Journal of Computer-Mediated Communication. 2010;15(3):500-25. doi: 10.1111/j.1083-6101.2010.01515.x.

21. Chiu TML, Eysenbach G. Stages of use: consideration, initiation, utilization, and outcomes of an internet-mediated intervention. BMC Medical Informatics & Decision Making. 2010;10(1):73-. PMID: 104997449. . doi: 10.1186/1472-6947-10-73.

22. Cho H, Flynn G, Saylor M, Gradilla M, Schnall R. Use of the FITT framework to understand patients' experiences using a real-time medication monitoring pill bottle linked to a mobile-based HIV self-management app: A qualitative study. Int J Med Inform. 2019 Nov;131(103949):103949. PMID: 31561192. doi: 10.1016/j.ijmedinf.2019.08.009.

23. Christie HL, Boots LMM, Peetoom K, Tange HJ, Verhey FRJ, de Vugt ME. Developing a Plan for the Sustainable Implementation of an Electronic Health Intervention (Partner in Balance) to Support Caregivers of People With Dementia: Case Study. JMIR Aging. 2020 Jun 25;3(1):e18624. PMID: 32584261. doi: 10.2196/18624.

24. Clarke AL, Roscoe J, Appleton R, Dale J, Nanton V. "My gut feeling is we could do more..." a qualitative study exploring staff and patient perspectives before and after the implementation of an online prostate cancer-specific holistic needs assessment. BMC Health Services Research. 2019;19(1):N.PAG-N.PAG. PMID: 134666323. Language: English. Entry Date: In Process. Revision Date: 20210111. Publication Type: journal article. Journal Subset: Biomedical. doi: 10.1186/s12913-019-3941-4.

25. Cohn WF, Canan CE, Knight S, Waldman AL, Dillingham R, Ingersoll K, et al. An Implementation Strategy to Expand Mobile Health Use in HIV Care Settings: Rapid Evaluation Study Using the Consolidated Framework for Implementation Research. JMIR Mhealth Uhealth. 2021 Apr 28;9(4):e19163. PMID: 33908893. doi: 10.2196/19163.

26. Cox NS, Scrivener K, Holland AE, Jolliffe L, Wighton A, Nelson S, et al. A Brief Intervention to Support Implementation of Telerehabilitation by Community Rehabilitation Services During COVID-19: A Feasibility Study. Archives of Physical Medicine and Rehabilitation. 2021. PMID: 2010787239. doi: http://dx.doi.org/10.1016/j.apmr.2020.12.007.

27. Crane ME, Phillips KE, Maxwell CA, Norris LA, Rifkin LS, Blank JM, et al. A Qualitative Examination of a School-Based Implementation of Computer-Assisted Cognitive-Behavioral Therapy for Child Anxiety. School Ment Health. 2021 Jun;13(2):347-61. PMID: 34178162. doi: 10.1007/s12310-021-09424-y.

28. Croff RL, Iv PW, Walker ML, Francois E, Quinn C, Riley TC, et al. Things Are Changing so Fast: Integrative Technology for Preserving Cognitive Health and Community History. Gerontologist. 2019;59(1):147-57. PMID: 134066185. doi: 10.1093/geront/gny069.

29. Curtis AC, Satre DD, Ly K, Wamsley M, Satterfield J. Implementation of alcohol and drug screening, brief intervention, and referral to treatment: Nurse practitioner learner perspectives on a mobile app. Journal of the American Association of Nurse Practitioners. 2019;31(4):219-25. PMID: 139245017. doi: 10.1097/jxx.0000000000000136.

30. Damschroder LJ, Reardon CM, Sperber N, Robinson CH, Fickel JJ, Oddone EZ. Implementation evaluation of the Telephone Lifestyle Coaching (TLC) program: organizational factors associated with successful implementation. Transl Behav Med. 2017 Jun;7(2):233-41. PMID: 27688249. doi: 10.1007/s13142-016-0424-6.

31. Darcy S, Maxwell H, Green J. Disability citizenship and independence through mobile technology? A study exploring adoption and use of a mobile technology platform. Disability & Society. 2016;31(4):497-519. PMID: 116100232. Language: English. Entry Date: 20160617. Revision Date: 20190213. Publication Type: Article. doi: 10.1080/09687599.2016.1179172.

32. Davies SM, Jardine J, Gutridge K, Bernard Z, Park S, Dawson T, et al. Preventive Digital Mental Health for Children in Primary Schools: Acceptability and Feasibility Study. JMIR Form Res. 2021 Dec 13;5(12):e30668. PMID: 34898446. doi: 10.2196/30668.

33. Day M, Demiris G, Oliver DP, Courtney K, Hensel B. Exploring underutilization of videophones in hospice settings. Telemedicine Journal and e-Health. 2007;13(1):25-31. PMID: 46427769. doi: http://dx.doi.org/10.1089/tmj.2006.0023.

34. den Bakker CM, Huirne JA, Schaafsma FG, de Geus C, Bonjer HJ, Anema JR. Electronic Health Program to Empower Patients in Returning to Normal Activities After Colorectal Surgical Procedures: Mixed-Methods Process Evaluation Alongside a Randomized Controlled Trial. Journal of medical Internet research. 2019 Jan 29;21(1):e10674. PMID: 30694205. doi: 10.2196/10674.

35. Dharmayat KI, Tran T, Hardy V, Chirambo BG, Thompson MJ, Ide N, et al. Sustainability of 'mHealth' interventions in sub- Saharan Africa: a stakeholder analysis of an electronic community case management project in Malawi. Malawi Med J. 2019 Sep;31(3):177-83. PMID: 31839886. doi: 10.4314/mmj.v31i3.3.

36. Dijkstra A, Heida A, van Rheenen PF. Exploring the Challenges of Implementing a Web-Based Telemonitoring Strategy for Teenagers With Inflammatory Bowel Disease: Empirical Case Study. J Med Internet Res. 2019 Mar 29;21(3):e11761. PMID: 30924785. doi: 10.2196/11761.

37. Drabble SJ, O'Cathain A, Scott AJ, Arden MA, Keating S, Hutchings M, et al. Mechanisms of Action of a Web-Based Intervention With Health Professional Support to Increase Adherence to Nebulizer Treatments in Adults With Cystic Fibrosis: Qualitative Interview Study. Journal of Medical Internet Research. 2020;22(10):N.PAG-N.PAG. PMID: 146783896. Language: English. Entry Date: 20201219. Revision Date: 20210131. Publication Type: journal article. doi: 10.2196/16782.

38. Dugstad J, Eide T, Nilsen ER, Eide H. Towards successful digital transformation through co-creation: a longitudinal study of a four-year implementation of digital monitoring technology in residential care for persons with dementia. BMC Health Serv Res. 2019 Jun 10;19(1):366. PMID: 31182093. doi: 10.1186/s12913-019-4191-1.

39. Ebenso B, Okusanya B, Okunade K, Akeju D, Ajepe A, Akaba GO, et al. What Are the Contextual Enablers and Impacts of Using Digital Technology to Extend Maternal and Child Health Services to Rural Areas? Findings of a Qualitative Study From Nigeria. Frontiers in global women's health. 2021;2:670494. doi: https://dx.doi.org/10.3389/fgwh.2021.670494.

40. Ehteshami A. Barcode technology acceptance and utilization in health information management department at academic hospitals according to technology acceptance model. Acta informatica medica. 2017;25(1):4-8. PMID: 28484289.

41. El Joueidi S, Bardosh K, Musoke R, Tilahun B, Abo Moslim M, Gourlay K, et al. Evaluation of the implementation process of the mobile health platform 'WelTel' in six sites in East Africa and Canada using the modified consolidated framework for implementation research (mCFIR). BMC Med Inform Decis Mak. 2021 Oct 26;21(1):293. PMID: 34702229. doi: 10.1186/s12911-021-01644-1.

42. Emani S, Peters E, Desai S, Karson AS, Lipsitz SR, LaRocca R, et al. Who adopts a patient portal?: An application of the diffusion of innovation model. J Innov Health Inform. 2018 Oct 25;25(3):149-57. PMID: 30398458. doi: 10.14236/jhi.v25i3.991.

43. Farr M, Banks J, Edwards HB, Northstone K, Bernard E, Salisbury C, et al. Implementing online consultations in primary care: a mixed-method evaluation extending normalisation process theory through service co-production. BMJ Open. 2018 Mar 19;8(3):e019966. PMID: 29555817. doi: 10.1136/bmjopen-2017-019966.

44. Farr M, Pithara C, Sullivan S, Edwards H, Hall W, Gadd C, et al. Pilot implementation of co-designed software for co-production in mental health care planning: a qualitative evaluation of staff perspectives. J Ment Health. 2019 Oct;28(5):495-504. PMID: 31240971. doi: 10.1080/09638237.2019.1608925.

45. Fehrenbacher C, Schoeny ME, Reed M, Shattell M, Breitenstein SM. Referral to Digital Parent Training in Primary Care: Facilitators and Barriers. Clin Pract Pediatr Psychol. 2020 Sep;8(3):268-77. PMID: 35821979. doi: 10.1037/cpp0000367.

46. Gagnon MP, Orruno E, Asua J, Abdeljelil AB, Emparanza J. Using a modified technology acceptance model to evaluate healthcare professionals' adoption of a new telemonitoring system. Telemedicine journal and e-health. 2012 Jan-Feb;18(1):54-9. PMID: 22082108. doi: 10.1089/tmj.2011.0066.

47. Garg SK, Lyles CR, Ackerman S, Handley MA, Schillinger D, Gourley G, et al. Qualitative analysis of programmatic initiatives to text patients with mobile devices in resource-limited health systems. BMC Med Inform Decis Mak. 2016 Feb 6;16:16. PMID: 26851941. doi: 10.1186/s12911-016-0258-7.

48. Geerds MAJ, Nijmeijer WS, Hegeman JH, Vollenbroek-Hutten MMR. Mobile App for Monitoring 3-Month Postoperative Functional Outcome After Hip Fracture: Usability Study. JMIR Human Factors. 2020 Sep 14;7(3):e16989. PMID: 32924949. doi: https://dx.doi.org/10.2196/16989.

49. Gong E, Sun L, Long Q, Xu H, Gu W, Bettger JP, et al. The Implementation of a Primary Care-Based Integrated Mobile Health Intervention for Stroke Management in Rural China: Mixed-Methods Process Evaluation. Front Public Health. 2021;9:774907. PMID: 34869187. doi: 10.3389/fpubh.2021.774907.

50. Hadjistavropoulos HD, Nugent MM, Dirkse D, Pugh N. Implementation of internet-delivered cognitive behavior therapy within community mental health clinics: a process evaluation using the consolidated framework for implementation research. BMC Psychiatry. 2017 Sep 12;17(1):331. PMID: 28899365. doi: 10.1186/s12888-017-1496-7.

51. Hale-Gallardo JL, Kreider CM, Jia H, Castaneda G, Freytes IM, Cowper Ripley DC, et al. Telerehabilitation for Rural Veterans: A Qualitative Assessment of Barriers and Facilitators to Implementation. J Multidiscip Healthc. 2020;13:559-70. PMID: 32669850. doi: 10.2147/JMDH.S247267.

52. Haque MS, Kangas M, Jamsa T. A persuasive mhealth behavioral change intervention for promoting physical activity in the workplace: Feasibility randomized controlled trial. Journal of Medical Internet Research. 2020;4(5). PMID: 2005811030. doi: http://dx.doi.org/10.2196/15083.

53. Harris BS, Melton B, Bland H, Carpentier A, Gonzales J, Catenacci K. Enhancing Psychosocial Constructs Associated with Technology-Based Physical Activity: A Randomized Trial Among African American Women. American Journal of Health Education. 2018;49(2):74-85. PMID: 128375908. Language: English. Entry Date: 20180314. Revision Date: 20190823. Publication Type: Article. doi: 10.1080/19325037.2017.1414642.

54. Harry ML, Truitt AR, Saman DM, Henzler-Buckingham HA, Allen CI, Walton KM, et al. Barriers and facilitators to implementing cancer prevention clinical decision support in primary care: a qualitative study. BMC Health Serv Res. 2019 Jul 31;19(1):534. PMID: 31366355. doi: 10.1186/s12913-019-4326-4.

55. Harsha P, Paul JE, Chong MA, Buckley N, Tidy A, Clarke A, et al. Challenges With Continuous Pulse Oximetry Monitoring and Wireless Clinician Notification Systems After Surgery: Reactive Analysis of a Randomized Controlled Trial. JMIR Med Inform. 2019 Oct 28;7(4):e14603. PMID: 31661079. doi: 10.2196/14603.

56. Haun MW, Stephan I, Wensing M, Hartmann M, Hoffmann M, Friederich HC. Intent to Adopt Video-Based Integrated Mental Health Care and the Characteristics of its Supporters: Mixed Methods Study Among General Practitioners Applying Diffusion of Innovations Theory. JMIR Ment Health. 2020 Oct 15;7(10):e23660. PMID: 33055058. doi: 10.2196/23660.

57. Haverhals LM, Sayre G, Helfrich CD, Battaglia C, Aron D, Stevenson LD, et al. E-consult implementation: lessons learned using consolidated framework for implementation research. Am J Manag Care. 2015 Dec 1;21(12):e640-7. PMID: 26760426.

58. Helitzer D, Heath D, Maltrud K, Sullivan E, Alverson D. Assessing or predicting adoption of telehealth using the diffusion of innovations theory: a practical example from a rural program in New Mexico. Telemed J E Health. 2003 Summer;9(2):179-87. PMID: 12855040. doi: 10.1089/153056203766437516.

59. Hostgaard AMB, Bertelsen P, Nohr C. Constructive eHealth evaluation: lessons from evaluation of EHR development in 4 Danish hospitals. BMC Med Inform Decis Mak. 2017 Apr 20;17(1):45. PMID: 28427407. doi: 10.1186/s12911-017-0444-2.

60. Jaana M, Sherrard H, Paré G. A prospective evaluation of telemonitoring use by seniors with chronic heart failure: Adoption, self-care, and empowerment. Health Informatics Journal. 2019;25(4):1800-14. PMID: 138883073. . doi: 10.1177/1460458218799458.

61. Jeon E, Park HA. Experiences of Patients With a Diabetes Self-Care App Developed Based on the Information-Motivation-Behavioral Skills Model: Before-and-After Study. JMIR Diabetes. 2019;4(2):e11590. PMID: 30998218. doi: https://dx.doi.org/10.2196/11590.

62. Jewer J, Parsons MH, Dunne C, Smith A, Dubrowski A. Evaluation of a Mobile Telesimulation Unit to Train Rural and Remote Practitioners on High-Acuity Low-Occurrence Procedures: Pilot Randomized Controlled Trial. J Med Internet Res. 2019 Aug 6;21(8):e14587. PMID: 31389340. doi: 10.2196/14587.

63. Johnson NL, Lerret S, Klingbeil CG, Polfuss M, Gibson C, Gralton K, et al. Engaging Parents in Education for Discharge (ePED): Evaluating the Reach, Adoption & Implementation of an Innovative Discharge Teaching Method. J Pediatr Nurs. 2020 Sep-Oct;54:42-9. PMID: 32531681. doi: 10.1016/j.pedn.2020.05.022.

64. Jones CH, Glogowska M, Locock L, Lasserson DS. Embedding new technologies in practice - a normalization process theory study of point of care testing. BMC Health Serv Res. 2016 Oct 19;16(1):591. PMID: 27756282. doi: 10.1186/s12913-016-1834-3.

65. Kinshella M-LW, Sheikh S, Bawani S, La M, Sharma S, Vidler M, et al. "Now You Have Become Doctors": Lady Health Workers' Experiences Implementing an mHealth Application in Rural Pakistan. Frontiers in global women's health. 2021;2:645705. doi: https://dx.doi.org/10.3389/fgwh.2021.645705.

66. Knoerl R, Dudley WN, Smith G, Bridges C, Kanzawa-Lee G, Lavoie Smith EM. Pilot Testing a Web-Based System for the Assessment and Management of Chemotherapy-Induced Peripheral Neuropathy. Comput Inform Nurs. 2017 Apr;35(4):201-11. PMID: 28002115. doi: 10.1097/CIN.0000000000000320.

67. Knox M, Murphy EJ, Leslie T, Wick R, Tuot DS. e-Consult implementation success: lessons from 5 county-based delivery systems. Am J Manag Care. 2020 Jan 1;26(1):e21-e7. PMID: 31951363. doi: 10.37765/ajmc.2020.42149.

68. Koot D, Goh PSC, Lim RSM, Tian Y, Yau TY, Tan NC, et al. A Mobile Lifestyle Management Program (GlycoLeap) for People With Type 2 Diabetes: Single-Arm Feasibility Study. JMIR Mhealth Uhealth. 2019 May 24;7(5):e12965. PMID: 31127720. doi: 10.2196/12965.

69. Kozikowski A, Shotwell J, Wool E, Slaboda JC, Abrashkin KA, Rhodes K, et al. Care Team Perspectives and Acceptance of Telehealth in Scaling a Home-Based Primary Care Program: Qualitative Study. JMIR Aging. 2019 Jun 2;2(1):e12415. PMID: 31518266. doi: 10.2196/12415.

70. Kummer BR, Sweetnam C, Vickrey BG, Naasan G, Harvey D, Gallagher K, et al. Teleneurology Expansion in Response to the COVID-19 Outbreak at a Tertiary Health System in New York City. Neurol Clin Pract. 2021 Apr;11(2):e102-e11. PMID: 33842078. doi: 10.1212/CPJ.0000000000001057.

71. Lalitaphanit K, Theeraroungchaisri A. Factors affecting community pharmacy customers' decision to use personal health records via smartphone. Thai Journal of Pharmaceutical Sciences. 2016;40:163-7.

72. Lambert-Kerzner AC, Aasen DM, Overbey DM, Damschroder LJ, Henderson WG, Hammermeister KE, et al. Use of the consolidated framework for implementation research to guide dissemination and implementation of new technologies in surgery. J Thorac Dis. 2019 Mar;11(Suppl 4):S487-S99. PMID: 31032067. doi: 10.21037/jtd.2019.01.29.

73. Lamontagne ME, Best KL, Clarke T, Dumont FS, Noreau L. Implementation Evaluation of an Online Peer-Mentor Training Program for Individuals With Spinal Cord Injury. Top Spinal Cord Inj Rehabil. 2019 Fall;25(4):303-15. PMID: 31844382. doi: 10.1310/sci19-00002.

74. Laurie J, Blandford A. Making time for mindfulness. Int J Med Inform. 2016 Dec;96:38-50. PMID: 26965526. doi: 10.1016/j.ijmedinf.2016.02.010.

75. Lee K, Lim K, Jung SY, Ji H, Hong K, Hwang H, et al. Perspectives of Patients, Health Care Professionals, and Developers Toward Blockchain-Based Health Information Exchange: Qualitative Study. J Med Internet Res. 2020 Nov 13;22(11):e18582. PMID: 33185553. doi: 10.2196/18582.

76. Lennon MR, Bouamrane MM, Devlin AM, O'Connor S, O'Donnell C, Chetty U, et al. Readiness for Delivering Digital Health at Scale: Lessons From a Longitudinal Qualitative Evaluation of a National Digital Health Innovation Program in the United Kingdom. J Med Internet Res. 2017 Feb 16;19(2):e42. PMID: 28209558. doi: 10.2196/jmir.6900.

77. Lesselroth B, Adams K, Mastarone G, Tallett S, Ragland S, Laing A, et al. Applying the Effective Technology Use Model to Implementation of Electronic Consult Management Software. Stud Health Technol Inform. 2019;257:261-5. PMID: 30741206. doi: 10.3233/978-1-61499-951-5-261.

78. Levesque DA, Johnson JL, Prochaska JM. Teen Choices, an Online Stage-Based Program for Healthy, Nonviolent Relationships: Development and Feasibility Trial. Journal of School Violence. 2017;16(4):376-85. PMID: 125435850. . doi: 10.1080/15388220.2016.1147964.

79. Levinson AJ, Ayers S, Butler L, Papaioannou A, Marr S, Sztramko R. Barriers and Facilitators to Implementing Web-Based Dementia Caregiver Education From the Clinician's Perspective: Qualitative Study. JMIR Aging. 2020 Oct 2;3(2):e21264. PMID: 33006563. doi: 10.2196/21264.

80. Liddy C, Bello A, Cook J, Drimer N, Pilon MD, Farrell G, et al. Supporting the spread and scale-up of electronic consultation across Canada: cross-sectional analysis. BMJ Open. 2019 05 30;9(5):e028888. PMID: 31152043. doi: https://dx.doi.org/10.1136/bmjopen-2018-028888.

81. Lin JC, Lee TT, Mills ME. Evaluation of a Barcode Medication Administration Information System. Comput Inform Nurs. 2018 Dec;36(12):596-602. PMID: 30015644. doi: 10.1097/CIN.0000000000000459.

82. Liu MC, Lee CC. An Investigation of Pharmacists’ Acceptance of NHI-PharmaCloud in Taiwan. Journal of Medical Systems. 2018;42(11):1-. PMID: 132813831. . doi: 10.1007/s10916-018-1017-3.

83. Liverani M, Ir P, Perel P, Khan M, Balabanova D, Wiseman V. Assessing the potential of wearable health monitors for health system strengthening in low- and middle-income countries: a prospective study of technology adoption in Cambodia. Health Policy Plan. 2022 Sep 13;37(8):943-51. PMID: 35262172. doi: 10.1093/heapol/czac019.

84. Ljubicic V, Ketikidis PH, Lazuras L. Drivers of intentions to use healthcare information systems among health and care professionals. Health Informatics Journal. 2020;26(1):56-71. PMID: 143231201. . doi: 10.1177/1460458218813629.

85. Lord S, Moore SK, Ramsey A, Dinauer S, Johnson K. Implementation of a Substance Use Recovery Support Mobile Phone App in Community Settings: Qualitative Study of Clinician and Staff Perspectives of Facilitators and Barriers. JMIR Ment Health. 2016 Jun 28;3(2):e24. PMID: 27352884. doi: 10.2196/mental.4927.

86. Malik M, Kazi AF, Hussain A. Adoption of health technologies for effective health information system: Need of the hour for Pakistan. PLoS One. 2021;16(10):e0258081. PMID: 34618842. doi: 10.1371/journal.pone.0258081.

87. McAlearney AS, Hefner JL, Sieck CJ, Huerta TR. The journey through grief: insights from a qualitative study of electronic health record implementation. Health Serv Res. 2015 Apr;50(2):462-88. PMID: 25219627. doi: 10.1111/1475-6773.12227.

88. McCreesh-Toselli S, Torline J, Gouse H, Robbins RN, Mellins CA, Remien RH, et al. Staff Perceptions of Preimplementation Barriers and Facilitators to a Mobile Health Antiretroviral Therapy Adherence Counseling Intervention in South Africa: Qualitative Study. JMIR Mhealth Uhealth. 2021 Apr 6;9(4):e23280. PMID: 33821806. doi: 10.2196/23280.

89. McGovern M, Quinlan M, Doyle G, Moore G, Geiger S. Implementing a National Electronic Referral Program: Qualitative Study. JMIR Med Inform. 2018 Jul 18;6(3):e10488. PMID: 30021709. doi: 10.2196/10488.

90. Meeks DW, Takian A, Sittig DF, Singh H, Barber N. Exploring the sociotechnical intersection of patient safety and electronic health record implementation. J Am Med Inform Assoc. 2014 Feb;21(e1):e28-34. PMID: 24052536. doi: 10.1136/amiajnl-2013-001762.

91. Mei YY, Marquard J, Jacelon C, Defeo AL. Designing and evaluating an electronic patient falls reporting system: Perspectives for the implementation of health information technology in long-term residential care facilities. International Journal of Medical Informatics. 2013;82(11):e294-306. PMID: 104109328. . doi: 10.1016/j.ijmedinf.2011.03.008.

92. Meijer E, Korst JS, Oosting KG, Heemskerk E, Hermsen S, Willemsen MC, et al. "At least someone thinks I'm doing well": a real-world evaluation of the quit-smoking app StopCoach for lower socio-economic status smokers. Addict Sci Clin Pract. 2021 Jul 28;16(1):48. PMID: 34321088. doi: 10.1186/s13722-021-00255-5.

93. Meyer AJ, Armstrong-Hough M, Babirye D, Mark D, Turimumahoro P, Ayakaka I, et al. Implementing mHealth Interventions in a Resource-Constrained Setting: Case Study From Uganda. JMIR Mhealth Uhealth. 2020 Jul 13;8(7):e19552. PMID: 32673262. doi: 10.2196/19552.

94. Meyer AND, Thompson PJ, Khanna A, Desai S, Mathews BK, Yousef E, et al. Evaluating a mobile application for improving clinical laboratory test ordering and diagnosis. J Am Med Inform Assoc. 2018 Jul 1;25(7):841-7. PMID: 29688391. doi: 10.1093/jamia/ocy026.

95. Mishuris Rg PJMLHRFDSPDMTMDM. Using normalization process theory to understand work flow implications of decision support implementation across diverse primary care settings. Journal of general internal medicine. 2017;32(2):S370-. PMID: CN-01362523.

96. Myall M, May CR, Grimmett C, May CM, Calman L, Richardson A, et al. RESTORE: an exploratory trial of a web-based intervention to enhance self-management of cancer-related fatigue: findings from a qualitative process evaluation. BMC Med Inform Decis Mak. 2015 Nov 14;15:94. PMID: 26577690. doi: 10.1186/s12911-015-0214-y.

97. Nachum S, Gogia K, Clark S, Hsu H, Sharma R, Greenwald PW. An Evaluation of Kiosks for Direct-to-Consumer Telemedicine Using the National Quality Forum Assessment Framework. Telemed J E Health. 2021 Feb;27(2):178-83. PMID: 32589518. doi: 10.1089/tmj.2019.0318.

98. Naik AD, Lawrence B, Kiefer L, Ramos K, Utech A, Masozera N, et al. Building a primary care/research partnership: lessons learned from a telehealth intervention for diabetes and depression. Fam Pract. 2015 Apr;32(2):216-23. PMID: 25552674. doi: 10.1093/fampra/cmu084.

99. Nápoles AM, Appelle N, Kalkhoran S, Vijayaraghavan M, Alvarado N, Satterfield J. Perceptions of clinicians and staff about the use of digital technology in primary care: qualitative interviews prior to implementation of a computer-facilitated 5As intervention. BMC Medical Informatics & Decision Making. 2016;16:1-13. PMID: 114694812. . doi: 10.1186/s12911-016-0284-5.

100. Ndlovu K, Mauco KL, Keetile M, Kadimo K, Senyatso RY, Ntebela D, et al. Acceptance of the District Health Information System Version 2 Platform for Malaria Case-Based Surveillance By Health Care Workers in Botswana: Web-Based Survey. JMIR formative research. 2022;6(3):e32722. doi: https://dx.doi.org/10.2196/32722.

101. Newman L, Bidargaddi N, Schrader G. Service providers' experiences of using a telehealth network 12 months after digitisation of a large Australian rural mental health service. Int J Med Inform. 2016 Oct;94:8-20. PMID: 27573307. doi: 10.1016/j.ijmedinf.2016.05.006.

102. Newton A, Bagnell A, Rosychuk R, Duguay J, Wozney L, Huguet A, et al. A Mobile Phone-Based App for Use During Cognitive Behavioral Therapy for Adolescents With Anxiety (MindClimb): User-Centered Design and Usability Study. JMIR MHealth and UHealth. 2020;8(12):e18439. PMID: 33289671. doi: https://dx.doi.org/10.2196/18439.

103. Ngo V, Matsumoto CG, Joseph JG, Bell JF, Bold RJ, Davis A, et al. The Personal Health Network Mobile App for Chemotherapy Care Coordination: Qualitative Evaluation of a Randomized Clinical Trial. JMIR MHealth and UHealth. 2020;8(5):e16527. PMID: 32452814. doi: https://dx.doi.org/10.2196/16527.

104. Nyemba-Mudenda M, Chigona W. mHealth outcomes for pregnant mothers in Malawi: a capability perspective. Information Technology for Development. 2017;24(2):245-78. doi: 10.1080/02681102.2017.1397594.

105. Palermo TM, de la Vega R, Murray C, Law E, Zhou C. A digital health psychological intervention (WebMAP Mobile) for children and adolescents with chronic pain: results of a hybrid effectiveness-implementation stepped-wedge cluster randomized trial. Pain. 2020 Dec;161(12):2763-74. PMID: 32658147. doi: 10.1097/j.pain.0000000000001994.

106. Paulsen MM, Varsi C, Paur I, Tangvik RJ, Andersen LF. Barriers and Facilitators for Implementing a Decision Support System to Prevent and Treat Disease-Related Malnutrition in a Hospital Setting: Qualitative Study. JMIR Form Res. 2019 May 9;3(2):e11890. PMID: 31094333. doi: 10.2196/11890.

107. Peels DA, van Stralen MM, Bolman C, Golsteijn RH, de Vries H, Mudde AN, et al. Development of web-based computer-tailored advice to promote physical activity among people older than 50 years. J Med Internet Res. 2012 Mar 2;14(2):e39. PMID: 22390878. doi: 10.2196/jmir.1742.

108. Pelletier AC, Jethwani K, Bello H, Kvedar J, Grant RW. Implementing a web-based home monitoring system within an academic health care network: barriers and facilitators to innovation diffusion. J Diabetes Sci Technol. 2011 Jan 1;5(1):32-8. PMID: 21303622. doi: 10.1177/193229681100500105.

109. Peracca SB, Jackson GL, Lamkin RP, Mohr DC, Zhao M, Lachica O, et al. Implementing Teledermatology for Rural Veterans: An Evaluation Using the RE-AIM Framework. Telemed J E Health. 2021 Feb;27(2):218-26. PMID: 32343924. doi: 10.1089/tmj.2020.0013.

110. Pérez-Rodríguez R, Moreno-Sánchez PA, Valdés-Aragonés M, Oviedo-Briones M, Divan S, García-Grossocordón N, et al. FriWalk robotic walker: usability, acceptance and UX evaluation after a pilot study in a real environment. Disability & Rehabilitation: Assistive Technology. 2020;15(6):718-27. PMID: 144711108. . doi: 10.1080/17483107.2019.1617795.

111. Piera-Jimenez J, Etzelmueller A, Kolovos S, Folkvord F, Lupianez-Villanueva F. Guided Internet-Based Cognitive Behavioral Therapy for Depression: Implementation Cost-Effectiveness Study. J Med Internet Res. 2021 May 11;23(5):e27410. PMID: 33973857. doi: 10.2196/27410.

112. Psihogios AM, King-Dowling S, O'Hagan B, Darabos K, Maurer L, Young J, et al. Contextual Predictors of Engagement in a Tailored mHealth Intervention for Adolescent and Young Adult Cancer Survivors. Ann Behav Med. 2021 Nov 18;55(12):1220-30. PMID: 33674863. doi: 10.1093/abm/kaab008.

113. Rageliene T, Aschemann-Witzel J, Gronhoj A. Efficacy of a smartphone application-based intervention for encouraging children's healthy eating in Denmark. Health Promot Int. 2022 Feb 17;37(1):10. PMID: 34245283. doi: 10.1093/heapro/daab081.

114. Renard M, Gaboury I, Michaud F, Tousignant M. The acceptability of two remote monitoring modalities for patients waiting for services in a physiotherapy outpatient clinic. Musculoskeletal Care. 2022 Sep;20(3):616-24. PMID: 35142425. doi: 10.1002/msc.1622.

115. Robins LS, Jackson JE, Green BB, Korngiebel D, Force RW, Baldwin LM. Barriers and facilitators to evidence-based blood pressure control in community practice. J Am Board Fam Med. 2013 Sep-Oct;26(5):539-57. PMID: 24004706. doi: 10.3122/jabfm.2013.05.130060.

116. Rogers E, Aidasani SR, Friedes R, Hu L, Langford AT, Moloney DN, et al. Barriers and Facilitators to the Implementation of a Mobile Insulin Titration Intervention for Patients With Uncontrolled Diabetes: A Qualitative Analysis. JMIR Mhealth Uhealth. 2019 Jul 31;7(7):e13906. PMID: 31368439. doi: 10.2196/13906.

117. Sadasivam RS, Hogan TP, Volkman JE, Smith BM, Coley HL, Williams JH, et al. Implementing point of care "e-referrals" in 137 clinics to increase access to a quit smoking internet system: the Quit-Primo and National Dental PBRN HI-QUIT Studies. Transl Behav Med. 2013 Dec;3(4):370-8. PMID: 24294325. doi: 10.1007/s13142-013-0230-3.

118. Sassen B, Kok G, Schepers J, Vanhees L. Supporting health care professionals to improve the processes of shared decision making and self-management in a web-based intervention: randomized controlled trial. Journal of Medical Internet Research. 2014 Oct 21;16(10):e211. PMID: 25337988. doi: 10.2196/jmir.3170.

119. Schooley B, Abed Y, Murad A, Horan TA, Roberts J. Design and field test of an mHealth system for emergency medical services. Health and Technology. 2013 December;3(4):327-40. PMID: 370405366. doi: 10.1007/s12553-013-0064-5.

120. Schultz K, Vickery H, Campbell K, Wheeldon M, Barrett-Beck L, Rushbrook E. Implementation of a virtual ward as a response to the COVID-19 pandemic. Aust Health Rev. 2021 Aug;45(4):433-41. PMID: 33840420. doi: 10.1071/AH20240.

121. Seljelid B, Varsi C, Solberg Nes L, Oystese KA, Borosund E. A Digital Patient-Provider Communication Intervention (InvolveMe): Qualitative Study on the Implementation Preparation Based on Identified Facilitators and Barriers. J Med Internet Res. 2021 Apr 8;23(4):e22399. PMID: 33830063. doi: 10.2196/22399.

122. Shaw RJ, Kaufman MA, Bosworth HB, Weiner BJ, Zullig LL, Lee SY, et al. Organizational factors associated with readiness to implement and translate a primary care based telemedicine behavioral program to improve blood pressure control: the HTN-IMPROVE study. Implementation Science. 2013 Sep 8;8(1):106. PMID: 24010683. doi: 10.1186/1748-5908-8-106.

123. Shaw S, Wherton J, Vijayaraghavan S, Morris J, Bhattacharya S, Hanson P, et al. Advantages and limitations of virtual online consultations in a NHS acute trust: the VOCAL mixed-methods study. Southampton (UK). NIHR Journals Library Health Services and Delivery Research. 2018. PMID: 29939519. doi: https://dx.doi.org/10.3310/hsdr06210.

124. Shea CM, Tabriz AA, Turner K, North S, Reiter KL. Telestroke Adoption Among Community Hospitals in North Carolina: A Cross-Sectional Study. J Stroke Cerebrovasc Dis. 2018 Sep;27(9):2411-7. PMID: 29784607. doi: 10.1016/j.jstrokecerebrovasdis.2018.04.032.

125. Sheshadri A, Makhnoon S, Alousi AM, Bashoura L, Andrade R, Miller CJ, et al. Home-Based Spirometry Telemonitoring After Allogeneic Hematopoietic Cell Transplantation: Mixed Methods Evaluation of Acceptability and Usability. JMIR formative research. 2022;6(2):e29393. doi: https://dx.doi.org/10.2196/29393.

126. Sockolow PS, Bowles KH, Lehmann HP, Abbott PA, Weiner JP. Community-based, interdisciplinary geriatric care team satisfaction with an electronic health record: a multimethod study. Comput Inform Nurs. 2012 Jun;30(6):300-11. PMID: 22411417. doi: 10.1097/NCN.0b013e31823eb561.

127. Spaulding A, Nordan L, Blanchfield L, Asiedu GB, Saltivan J, Pecenka S, et al. Qualitative study of implementation of patient self-reported measures in a consultation-liaison psychiatry practice. J Eval Clin Pract. 2019 Jun;25(3):482-90. PMID: 31037796. doi: 10.1111/jep.13160.

128. Stara V, Vera B, Bolliger D, Rossi L, Felici E, Di Rosa M, et al. Usability and Acceptance of the Embodied Conversational Agent Anne by People With Dementia and Their Caregivers: Exploratory Study in Home Environment Settings. JMIR Mhealth Uhealth. 2021 Jun 25;9(6):e25891. PMID: 34170256. doi: 10.2196/25891.

129. Steele Gray C, Chau E, Tahsin F, Harvey S, Loganathan M, McKinstry B, et al. Assessing the Implementation and Effectiveness of the Electronic Patient-Reported Outcome Tool for Older Adults With Complex Care Needs: Mixed Methods Study. J Med Internet Res. 2021 Dec 2;23(12):e29071. PMID: 34860675. doi: 10.2196/29071.

130. Stevenson L, Ball S, Haverhals LM, Aron DC, Lowery J. Evaluation of a national telemedicine initiative in the Veterans Health Administration: Factors associated with successful implementation. J Telemed Telecare. 2018 Apr;24(3):168-78. PMID: 27909208. doi: 10.1177/1357633X16677676.

131. Theys S, Lust E, Heinen M, Verhaeghe S, Beeckman D, Eeckloo K, et al. Barriers and enablers for the implementation of a hospital communication tool for patient participation: A qualitative study. J Clin Nurs. 2020 Jun;29(11-12):1945-56. PMID: 31494999. doi: 10.1111/jocn.15055.

132. Tobler N. Technology, organizational change, and the nonhuman agent: Exploratory analysis of electronic health record implementation in a small practice ambulatory care. Dissertation Abstracts International Section A: Humanities and Social Sciences. 2008;69(5-A).

133. Umstead CN, Unertl KM, Lorenzi NM, Novak LL. Enabling adoption and use of new health information technology during implementation: Roles and strategies for internal and external support personnel. Journal of the American Medical Informatics Association. 2021 07 14;28(7):1543-7. PMID: 33893511. doi: https://dx.doi.org/10.1093/jamia/ocab044.

134. van den Berg MH, van der Giesen FJ, van Zeben D, van Groenendael JH, Seys PE, Vliet Vlieland TP. Implementation of a physical activity intervention for people with rheumatoid arthritis: a case study. Musculoskeletal Care. 2008;6(2):69-85. PMID: 105664963. . doi: 10.1002/msc.128.

135. van der Kamp M, Reimering Hartgerink P, Driessen J, Thio B, Hermens H, Tabak M. Feasibility, Efficacy, and Efficiency of eHealth-Supported Pediatric Asthma Care: Six-Month Quasi-Experimental Single-Arm Pretest-Posttest Study. JMIR Formative Research. 2021 Jul 26;5(7):e24634. PMID: 34309568. doi: https://dx.doi.org/10.2196/24634.

136. van Gaalen JL, van Bodegom-Vos L, Bakker MJ, Snoeck-Stroband JB, Sont JK. Internet-based self-management support for adults with asthma: a qualitative study among patients, general practitioners and practice nurses on barriers to implementation. BMJ Open. 2016 08 26;6(8):e010809. PMID: 27566627. doi: https://dx.doi.org/10.1136/bmjopen-2015-010809.

137. van Lieshout F, Yang R, Stamenova V, Agarwal P, Cornejo Palma D, Sidhu A, et al. Evaluating the Implementation of a Remote-Monitoring Program for Chronic Obstructive Pulmonary Disease: Qualitative Methods from a Service Design Perspective. J Med Internet Res. 2020 Oct 9;22(10):e18148. PMID: 33034565. doi: 10.2196/18148.

138. Vandenkerkhof EG, Hall S, Wilson R, Gay A, Duhn L. Evaluation of an innovative communication technology in an acute care setting. CIN: Computers, Informatics, Nursing. 2009;27(4):254-62. PMID: 105393572. . doi: 10.1097/NCN.0b013e3181a91bf6.

139. Varsi C, Ekstedt M, Gammon D, Ruland CM. Using the Consolidated Framework for Implementation Research to Identify Barriers and Facilitators for the Implementation of an Internet-Based Patient-Provider Communication Service in Five Settings: A Qualitative Study. J Med Internet Res. 2015 Nov 18;17(11):e262. PMID: 26582138. doi: 10.2196/jmir.5091.

140. Vasalampi A. Adoption and Use of a Mobile System at Home Care. Studies in Health Technology & Informatics. 2017;242:1042-6. PMID: 134729642. . doi: 10.3233/978-1-61499-798-6-1042.

141. Verhoeven F, Steehouder MF, Hendrix RM, van Gemert-Pijnen JE. Factors affecting health care workers' adoption of a website with infection control guidelines. Int J Med Inform. 2009 Oct;78(10):663-78. PMID: 19577956. doi: 10.1016/j.ijmedinf.2009.06.001.

142. Vetter MJ. The Influence of Clinical Decision Support on Diagnostic Accuracy in Nurse Practitioners. Worldviews Evid Based Nurs. 2015 Dec;12(6):355-63. PMID: 26630088. doi: 10.1111/wvn.12121.

143. Vriend I, Coehoorn I, Verhagen E. Implementation of an app-based neuromuscular training programme to prevent ankle sprains: a process evaluation using the RE-AIM Framework. Br J Sports Med. 2015 Apr;49(7):484-8. PMID: 24470587. doi: 10.1136/bjsports-2013-092896.

144. Vuorinen M. Registered Nurses' experiences with, and feelings and attitudes towards, the International Resident Assessment Instrument for Long-Term Care Facilities in New Zealand in 2017. Journal of Research in Nursing. 2020;25(2):141-55. PMID: 142247530. . doi: 10.1177/1744987119890651.

145. Wang SL, Lin HI. Integrating TTF and IDT to evaluate user intention of big data analytics in mobile cloud healthcare system. Behaviour & Information Technology. 2019;38(9):974-85. PMID: 138138852. . doi: 10.1080/0144929x.2019.1626486.

146. Ware P, Ross HJ, Cafazzo JA, Laporte A, Gordon K, Seto E. Evaluating the Implementation of a Mobile Phone-Based Telemonitoring Program: Longitudinal Study Guided by the Consolidated Framework for Implementation Research. JMIR Mhealth Uhealth. 2018 Jul 31;6(7):e10768. PMID: 30064970. doi: 10.2196/10768.

147. Warner G, Lawson B, Sampalli T, Burge F, Gibson R, Wood S. Applying the consolidated framework for implementation research to identify barriers affecting implementation of an online frailty tool into primary health care: a qualitative study. BMC Health Serv Res. 2018 May 31;18(1):395. PMID: 29855306. doi: 10.1186/s12913-018-3163-1.

148. Watkinson F, Dharmayat KI, Mastellos N. A mixed-method service evaluation of health information exchange in England: technology acceptance and barriers and facilitators to adoption. BMC Health Services Research. 2021 Jul 25;21(1):737. PMID: 34303379. doi: https://dx.doi.org/10.1186/s12913-021-06771-z.

149. Webb MJ, Wadley G, Sanci LA. Experiences of General Practitioners and Practice Support Staff Using a Health and Lifestyle Screening App in Primary Health Care: Implementation Case Study. JMIR Mhealth Uhealth. 2018 Apr 24;6(4):e105. PMID: 29691209. doi: 10.2196/mhealth.8778.

150. Whittaker AA, Aufdenkamp M, Tinley S. Barriers and facilitators to electronic documentation in a rural hospital. J Nurs Scholarsh. 2009 2009 3rd Quarter;41(3):293-300. PMID: 19723278. doi: 10.1111/j.1547-5069.2009.01278.x.

151. Williams KM, Kirsh S, Aron D, Au D, Helfrich C, Lambert-Kerzner A, et al. Evaluation of the Veterans Health Administration's Specialty Care Transformational Initiatives to Promote Patient-Centered Delivery of Specialty Care: A Mixed-Methods Approach. Telemed J E Health. 2017 Jul;23(7):577-89. PMID: 28177858. doi: 10.1089/tmj.2016.0166.

152. Williamson A, Barbarin A, Campbell B, Campbell T, Franzen S, Reischl TM, et al. Uptake of and Engagement With an Online Sexual Health Intervention (HOPE eIntervention) Among African American Young Adults: Mixed Methods Study. J Med Internet Res. 2021 Jul 16;23(7):e22203. PMID: 34269689. doi: 10.2196/22203.

153. Workman A, Jones PJ, Wheeler AJ, Campbell SL, Williamson GJ, Lucani C, et al. Environmental Hazards and Behavior Change: User Perspectives on the Usability and Effectiveness of the AirRater Smartphone App. Int J Environ Res Public Health. 2021 Mar 30;18(7):30. PMID: 33808395. doi: 10.3390/ijerph18073591.

154. Yeshua-Katz D. The Role of Communication Affordances in Post-Traumatic Stress Disorder Facebook and WhatsApp Support Groups. Int J Environ Res Public Health. 2021 Apr 26;18(9). PMID: 33925904. doi: 10.3390/ijerph18094576.

155. Yu CH, Gall Casey C, Ke C, Lebovic G, Straus SE. Process Evaluation of the Diabetes Canada Guidelines Dissemination Strategy Using the Reach Effectiveness Adoption Implementation Maintenance (RE-AIM) Framework. Can J Diabetes. 2019 Jun;43(4):263-70 e9. PMID: 30415910. doi: 10.1016/j.jcjd.2018.08.189.

156. Yusof MM. A case study evaluation of a Critical Care Information System adoption using the socio-technical and fit approach. Int J Med Inform. 2015 Jul;84(7):486-99. PMID: 25881560. doi: 10.1016/j.ijmedinf.2015.03.001.
