# Supplementary material for: Mapping Theories, Models, and Frameworks to Evaluate Digital Health Interventions: Scoping Review
Source: J Med Internet Res. 2024 Feb 5;26:e51098. doi: 10.2196/51098 (PMC10877497; doi:10.2196/51098)
Supplement: Multimedia Appendix 5 [file jmir_v26i1e51098_app5.docx]

**Multimedia Appendix 5.** List of all TMFs (n=68) across all the studies (n=156)

| **Frameworks’ name as reported by authors** | **Number of studies that used that TMF** | **References** |
| --- | --- | --- |
| Consolidated Framework for Implementation Research (CFIR) | 39 | See Multimedia Appendix 6 |
| Reach Effectiveness Adoption Implementation Maintenance (RE-AIM) framework | 17 | See Multimedia Appendix 6 |
| Technology Acceptance Model (TAM) | 16 | See Multimedia Appendix 6 |
| Unified Theory of Acceptance and Use of Technology (UTAUT) | 11 | See Multimedia Appendix 6 |
| Diffusion of Innovation (DOI) Theory | 10 | See Multimedia Appendix 6 |
| Normalization Process Theory (NPT) | 9 | See Multimedia Appendix 6 |
| Non-adoption, Abandonment, Scale-up, Spread, Sustainability (NASSS) framework | 5 | [1-5] |
| Promoting Action on Research Implementation in the Health Service (PARiHS) Framework | 4 | [6-9] |
| Theoretical Domains Framework (TDF) | 4 | [10-13] |
| Promoting Action on Research Implementation in the Health Service (PARiHS) Framework | 4 | [6-9] |
| Theoretical Domains Framework (TDF) | 4 | [10-13] |
| Theory of Planned Behavior (TPB) | 3 | [14-16] |
| Kirkpatrick's Model for Evaluation | 3 | [17-19] |
| Self-Determination Theory (SDT) | 3 | [20-22] |
| Behaviour Change Wheel (BCW) | 2 | [23] |
| A conceptual framework illustrating the relationship between technological, environmental, organizational and human factors regarding the successful adoption of health information systems. | 1 | [24] |
| Adaptive Structuration Theory | 1 | [25] |
| Actor Network Theory | 1 | [25] |
| Affordance Approach | 1 | [26] |
| Almere Model | 1 | [27] |
| Behavioral Model of Health Service Use (BMHSU) | 1 | [28] |
| Capability Approach Theoretical Framework | 1 | [29] |
| Capability, Opportunity, Motivation, Behaviour (COM-B) Model | 1 | [11] |
| Chronic Care Model | 1 | [30] |
| Clinical Adoption Framework | 1 | [31] |
| Constructive eHealth evaluation method (CeHEM) | 1 | [32] |
| Contingency Model | 1 | [33] |
| Curran et al.’s approach to Evidence-Based Quality Improvement (EBQI) | 1 | [34] |
| Determinants of Innovation (MIDI) framework | 1 | [35] |
| Effective Technology Use (ETU) | 1 | [36] |
| Expert Recommendations for Implementing Change | 1 | [37] |
| Evidence–Based Practice Improvement Model (EBPI) | 1 | [9] |
| Fit between Individuals, Task and Technology (FITT) framework | 1 | [38] |
| Grol and Wensing framework | 1 | [39] |
| Health Information Technology Research-Based Evaluation Framework (HITREF) | 1 | [40] |
| Healthcare Improvements Framework | 1 | [41] |
| Holistic human factors evaluation | 1 | [42] |
| Human-Organization-Technology Fit (HOT-Fit) | 1 | [43] |
| ICT4HC Model | 1 | [44] |
| Implementation Outcomes Framework | 1 | [45] |
| Information Systems Design Theory (ISDT) | 1 | [46] |
| Information Systems Success Models | 1 | [47] |
| Information-Motivation-Behavioural Skills Model of Diabetes Self-Care (IMB-DSC) | 1 | [48] |
| Interactive SocioTechnical Analysis (ITSA) Framework | 1 | [49] |
| Knowledge-to-Action framework | 1 | [50] |
| Mahlke's User Experience | 1 | [51] |
| mHealth Engagement Framework | 1 | [52] |
| Miller's Clinical Assessment Framework | 1 | [18] |
| Model for Assessment of Telemedicine | 1 | [53] |
| Monitoring and Assessment Framework for the European Innovation Partnership on Active and Healthy Ageing (MAFEIP) | 1 | [53] |
| National Quality Forum (NQF) Telehealth Assessment Framework | 1 | [54] |
| Organizational Change Management Model | 1 | [55] |
| Plan-Do-Study-Act (PDSA) framework | 1 | [9] |
| Policy, human/person, activity, assistance, technology and environment (PHAATE) model | 1 | [56] |
| Predisposing, Reinforcing, and Enabling Causes in Educational Diagnosis and Evaluation (PRECEDE) Model | 1 | [57] |
| Reasoned Action Approach | 1 | [58] |
| Sekhon's Acceptability Conceptual Framework | 1 | [59] |
| Shediac-Rizkallah and Bone framework | 1 | [60] |
| Social Cognitive Theory (SCT) | 1 | [21] |
| Sociotechnical Model | 1 | [61] |
| Staggers and Parks Nurse-Computer Interaction Framework | 1 | [62] |
| Strong Structuration Theory (SST) | 1 | [63] |
| Structuration Theory (ST) | 1 | [25] |
| Task Technology Fit (TTF) Theory | 1 | [64] |
| Technology, Organization, and Environment (TOE) framework | 1 | [65] |
| The transtheoretical model of behavior change | 1 | [66] |
| Theoretical framework is adapted from Chau and Hu’s model of telemedicine acceptance | 1 | [67] |
| Three-phase EHR safety model | 1 | [61] |
| Tool+Team+Routine Framework | 1 | [68] |
| USE IT-Interview Model | 1 | [69] |
| Weiner Organizational Theory of Implementation Effectiveness | 1 | [70] |

**References**

1. Abimbola S, Patel B, Peiris D, Patel A, Harris M, Usherwood T, et al. The NASSS framework for ex post theorisation of technology-supported change in healthcare: worked example of the TORPEDO programme. BMC Medicine. 2019;17(1):1-17. PMID: 140956184. . doi: 10.1186/s12916-019-1463-x.

2. Davies SM, Jardine J, Gutridge K, Bernard Z, Park S, Dawson T, et al. Preventive Digital Mental Health for Children in Primary Schools: Acceptability and Feasibility Study. JMIR formative research. 2021;5(12):e30668. PMID: 34898446. doi: https://dx.doi.org/10.2196/30668.

3. Dijkstra A, Heida A, Rheenen PFv, van Rheenen PF. Exploring the Challenges of Implementing a Web-Based Telemonitoring Strategy for Teenagers With Inflammatory Bowel Disease: Empirical Case Study. Journal of Medical Internet Research. 2019;21(3):e11761. PMID: 135716133. . doi: 10.2196/11761.

4. Liverani M, Por I, Perel P, Khan M, Balabanova D, Wiseman V. Assessing the Potential of Wearable Health Monitors for Health System Strengthening in Low- and Middle-Income Countries: A Prospective Study of Technology Adoption in Cambodia. Health policy and planning. 2022. doi: https://dx.doi.org/10.1093/heapol/czac019.

5. Schultz K, Vickery H, Campbell K, Wheeldon M, Barrett-Beck L, Rushbrook E. Implementation of a virtual ward as a response to the COVID-19 pandemic. Aust Health Rev. 2021 Aug;45(4):433-41. PMID: 33840420. doi: https://dx.doi.org/10.1071/AH20240.

6. Lee K, Lim K, Jung SY, Ji H, Hong K, Hwang H, et al. Perspectives of Patients, Health Care Professionals, and Developers Toward Blockchain-Based Health Information Exchange: Qualitative Study. Journal of Medical Internet Research. 2020;22(11). PMID: 147360168. doi: 10.2196/18582.

7. Naik AD, Lawrence B, Kiefer L, Ramos K, Utech A, Masozera N, et al. Building a primary care/research partnership: lessons learned from a telehealth intervention for diabetes and depression. Family Practice. 2015;32(2):216-23. PMID: 109714487. . doi: 10.1093/fampra/cmu084.

8. Sadasivam RS, Hogan TP, Volkman JE, Smith BM, Coley HL, Williams JH, et al. Implementing point of care "e-referrals" in 137 clinics to increase access to a quit smoking internet system: The Quit-Primo and National Dental PBRN HI-QUIT Studies. References. Translational Behavioral Medicine. 2013;3(4):370-8. doi: http://dx.doi.org/10.1007/s13142-013-0230-3.

9. Vetter MJ. The influence of clinical decision support on diagnostic accuracy in nurse practitioners. References. Worldviews on Evidence Based Nursing. 2015;12(6):355-63. PMID: 26630088. doi: http://dx.doi.org/10.1111/wvn.12121.

10. Clarke AL, Roscoe J, Appleton R, Dale J, Nanton V. "My gut feeling is we could do more..." a qualitative study exploring staff and patient perspectives before and after the implementation of an online prostate cancer-specific holistic needs assessment. BMC Health Services Research. 2019;19(1). PMID: 134666323. . doi: 10.1186/s12913-019-3941-4.

11. Cox NS, Scrivener K, Holland AE, Jolliffe L, Wighton A, Nelson S, et al. A Brief Intervention to Support Implementation of Telerehabilitation by Community Rehabilitation Services During COVID-19: A Feasibility Study. Archives of Physical Medicine and Rehabilitation. 2021. PMID: 2010787239. doi: http://dx.doi.org/10.1016/j.apmr.2020.12.007.

12. Drabble SJ, O'Cathain A, Scott AJ, Arden MA, Keating S, Hutchings M, et al. Mechanisms of Action of a Web-Based Intervention With Health Professional Support to Increase Adherence to Nebulizer Treatments in Adults With Cystic Fibrosis: Qualitative Interview Study. Journal of Medical Internet Research. 2020;22(10). PMID: 146783896. doi: 10.2196/16782.

13. Newton A, Bagnell A, Rosychuk R, Duguay J, Wozney L, Huguet A, et al. A Mobile Phone-Based App for Use During Cognitive Behavioral Therapy for Adolescents With Anxiety (MindClimb): User-Centered Design and Usability Study. JMIR MHealth and UHealth. 2020;8(12):e18439. PMID: 33289671. doi: https://dx.doi.org/10.2196/18439.

14. Curtis AC, Satre DD, Ly K, Wamsley M, Satterfield J. Implementation of alcohol and drug screening, brief intervention, and referral to treatment: Nurse practitioner learner perspectives on a mobile app. Journal of the American Association of Nurse Practitioners. 2019;31(4):219-25. PMID: 139245017. doi: 10.1097/jxx.0000000000000136.

15. Sassen B, Kok G, Schepers J, Vanhees L. Supporting health care professionals to improve the processes of shared decision making and self-management in a web-based intervention: randomized controlled trial. Journal of Medical Internet Research. 2014 Oct 21;16(10):e211. PMID: 25337988. doi: 10.2196/jmir.3170.

16. Vandenkerkhof EG, Hall S, Wilson R, Gay A, Duhn L. Evaluation of an innovative communication technology in an acute care setting. CIN: Computers, Informatics, Nursing. 2009;27(4):254-62. PMID: 105393572. . doi: 10.1097/NCN.0b013e3181a91bf6.

17. Chaves RO, de Oliveira PAV, Rocha LC, David JPF, Ferreira SC, de Assis Santos dos Santos A, et al. An Innovative Streaming Video System With a Point-of-View Head Camera Transmission of Surgeries to Smartphones and Tablets: An Educational Utility. Surgical Innovation. 2017;24(5):462-70. PMID: 125221918. doi: 10.1177/1553350617715162.

18. Jewer J, Parsons MH, Dunne C, Smith A, Dubrowski A. Evaluation of a Mobile Telesimulation Unit to Train Rural and Remote Practitioners on High-Acuity Low-Occurrence Procedures: Pilot Randomized Controlled Trial. Journal of Medical Internet Research. 2019;21(8). PMID: 138652980. . doi: 10.2196/14587.

19. Meyer AND, Thompson PJ, Khanna A, Desai S, Mathews BK, Yousef E, et al. Evaluating a mobile application for improving clinical laboratory test ordering and diagnosis. Journal of the American Medical Informatics Association. 2018;25(7):841-7. PMID: 130459175. . doi: 10.1093/jamia/ocy026.

20. Haque MS, Kangas M, Jamsa T. A persuasive mhealth behavioral change intervention for promoting physical activity in the workplace: Feasibility randomized controlled trial. Journal of Medical Internet Research. 2020;4(5). PMID: 2005811030. doi: http://dx.doi.org/10.2196/15083.

21. Harris BS, Melton B, Bland H, Carpentier A, Gonzales J, Catenacci K. Enhancing Psychosocial Constructs Associated with Technology-Based Physical Activity: A Randomized Trial Among African American Women. American Journal of Health Education. 2018;49(2):74-85. PMID: 128375908. . doi: 10.1080/19325037.2017.1414642.

22. Knoerl R, Dudley WN, Smith G, Bridges C, Kanzawa-Lee G, Lavoie Smith EM. Pilot Testing a Web-Based System for the Assessment and Management of Chemotherapy-Induced Peripheral Neuropathy. CIN: Computers, Informatics, Nursing. 2017;35(4):201-11. PMID: 123137208. .

23. Workman A, Jones PJ, Wheeler AJ, Campbell SL, Williamson GJ, Lucani C, et al. Environmental Hazards and Behavior Change: User Perspectives on the Usability and Effectiveness of the AirRater Smartphone App. Int J Environ Res Public Health. 2021 03 30;18(7):30. PMID: 33808395. doi: https://dx.doi.org/10.3390/ijerph18073591.

24. Malik M, Kazi AF, Hussain A. Adoption of health technologies for effective health information system: Need of the hour for Pakistan. PLoS ONE. 2021;16(10):e0258081. PMID: 34618842. doi: https://dx.doi.org/10.1371/journal.pone.0258081.

25. Tobler N. Technology, organizational change, and the nonhuman agent: Exploratory analysis of electronic health record implementation in a small practice ambulatory care. Dissertation Abstracts International Section A: Humanities and Social Sciences. 2008;69(5-A).

26. Yeshua-Katz D. The role of communication affordances in post-traumatic stress disorder facebook and whatsapp support groups. International Journal of Environmental Research and Public Health. 2021 01 May;18(9). PMID: 2007038857. doi: http://dx.doi.org/10.3390/ijerph18094576.

27. Stara V, Vera B, Bolliger D, Rossi L, Felici E, Di Rosa M, et al. Usability and Acceptance of the Embodied Conversational Agent Anne by People With Dementia and Their Caregivers: Exploratory Study in Home Environment Settings. JMIR MHealth and UHealth. 2021 06 25;9(6):e25891. PMID: 34170256. doi: https://dx.doi.org/10.2196/25891.

28. Chiu TML, Eysenbach G. Stages of use: consideration, initiation, utilization, and outcomes of an internet-mediated intervention. BMC Medical Informatics & Decision Making. 2010;10(1):73-. PMID: 104997449. . doi: 10.1186/1472-6947-10-73.

29. Nyemba-Mudenda M, Chigona W. mHealth outcomes for pregnant mothers in Malawi: A capability perspective. References. Information Technology for Development. 2018;24(2):245-78. doi: http://dx.doi.org/10.1080/02681102.2017.1397594.

30. Robins LS, Jackson JE, Green BB, Korngiebel D, Force RW, Baldwin LM. Barriers and Facilitators to Evidence-based Blood Pressure Control in Community Practice. Journal of the American Board of Family Medicine. 2013;26(5):539-57. PMID: 107911485. . doi: 10.3122/jabfm.2013.05.130060.

31. Harsha P, Paul JE, Chong MA, Buckley N, Tidy A, Clarke A, et al. Challenges With Continuous Pulse Oximetry Monitoring and Wireless Clinician Notification Systems After Surgery: Reactive Analysis of a Randomized Controlled Trial. JMIR Medical Informatics. 2019;7(4):e14603. PMID: 31661079. doi: https://dx.doi.org/10.2196/14603.

32. Høstgaard AMB, Bertelsen P, Nøhr C. Constructive eHealth evaluation: lessons from evaluation of EHR development in 4 Danish hospitals. BMC Medical Informatics & Decision Making. 2017;17:1-15. PMID: 122656490. . doi: 10.1186/s12911-017-0444-2.

33. Theys S, Lust E, Heinen M, Verhaeghe S, Beeckman D, Eeckloo K, et al. Barriers and enablers for the implementation of a hospital communication tool for patient participation: A qualitative study. Journal of Clinical Nursing (John Wiley & Sons, Inc). 2020;29(11/12):1945-56. PMID: 143305116. . doi: 10.1111/jocn.15055.

34. Adeoye-Olatunde OA, Curran GM, Jaynes HA, Hillman LA, Sangasubana N, Chewning BA, et al. Preparing for the spread of patient-reported outcome (PRO) data collection from primary care to community pharmacy: a mixed-methods study. Implementation science communications. 2022;3(1):29. doi: https://dx.doi.org/10.1186/s43058-022-00277-3.

35. Dugstad J, Eide T, Nilsen ER, Eide H. Towards successful digital transformation through co-creation: a longitudinal study of a four-year implementation of digital monitoring technology in residential care for persons with dementia. BMC Health Services Research. 2019;19(1):366. PMID: 31182093. doi: https://dx.doi.org/10.1186/s12913-019-4191-1.

36. Lesselroth B, Adams K, Mastarone G, Tallett S, Ragland S, Laing A, et al. Applying the Effective Technology Use Model to Implementation of Electronic Consult Management Software. Studies in Health Technology & Informatics. 2019;257:261-5. PMID: 134901606. Language: English. Entry Date: 20190227. Revision Date: 20201217. Publication Type: Article. doi: 10.3233/978-1-61499-951-5-261.

37. Kummer BR, Sweetnam C, Vickrey BG, Naasan G, Harvey D, Gallagher K, et al. Teleneurology Expansion in Response to the COVID-19 Outbreak at a Tertiary Health System in New York City. Neurol. 2021;11(2):e102-e11. PMID: 33842078. doi: https://dx.doi.org/10.1212/CPJ.0000000000001057.

38. Cho H, Flynn G, Saylor M, Gradilla M, Schnall R. Use of the FITT framework to understand patients' experiences using a real-time medication monitoring pill bottle linked to a mobile-based HIV self-management app: A qualitative study. International journal of medical informatics. 2019 Nov;131(103949). PMID: 31561192. doi: 10.1016/j.ijmedinf.2019.08.009.

39. van Gaalen JL, van Bodegom-Vos L, Bakker MJ, Snoeck-Stroband JB, Sont JK. Internet-based self-management support for adults with asthma: a qualitative study among patients, general practitioners and practice nurses on barriers to implementation. BMJ Open. 2016 08 26;6(8):e010809. PMID: 27566627. doi: https://dx.doi.org/10.1136/bmjopen-2015-010809.

40. Sockolow PS, Bowles KH, Lehmann HP, Abbott PA, Weiner JP. Community-based, interdisciplinary geriatric care team satisfaction with an electronic health record: a multimethod study. CIN: Computers, Informatics, Nursing. 2012;30(6):300-11. PMID: 108123363. .

41. McGovern M, Quinlan M, Doyle G, Moore G, Geiger S. Implementing a National Electronic Referral Program: Qualitative Study. JMIR Medical Informatics. 2018;6(3):e10488. PMID: 30021709. doi: https://dx.doi.org/10.2196/10488.

42. Mei YY, Marquard J, Jacelon C, Defeo AL. Designing and evaluating an electronic patient falls reporting system: Perspectives for the implementation of health information technology in long-term residential care facilities. International Journal of Medical Informatics. 2013;82(11):e294-306. PMID: 104109328. . doi: 10.1016/j.ijmedinf.2011.03.008.

43. Yusof MM. A case study evaluation of a Critical Care Information System adoption using the socio-technical and fit approach. International Journal of Medical Informatics. 2015;84(7):486-99. PMID: 109733455. . doi: 10.1016/j.ijmedinf.2015.03.001.

44. Chib A. The aceh besar midwives with mobile phones project: Design and evaluation perspectives using the information and communication technologies for healthcare development model. References. Journal of Computer Mediated Communication. 2010;15(3):500-25. doi: http://dx.doi.org/10.1111/j.1083-6101.2010.01515.x.

45. Ware P, Ross HJ, Cafazzo JA, Laporte A, Gordon K, Seto E. Evaluating the Implementation of a Mobile Phone-Based Telemonitoring Program: Longitudinal Study Guided by the Consolidated Framework for Implementation Research. JMIR MHealth and UHealth. 2018 Jul 31;6(7):e10768. PMID: 30064970. doi: https://dx.doi.org/10.2196/10768.

46. Schooley B, Abed Y, Murad A, Horan TA, Roberts J. Design and field test of an mHealth system for emergency medical services. Health and Technology. 2013 December;3(4):327-40. PMID: 370405366. doi: http://dx.doi.org/10.1007/s12553-013-0064-5.

47. Lin JC, Lee TT, Mills ME. Evaluation of a Barcode Medication Administration Information System. CIN: Computers, Informatics, Nursing. 2018;36(12):596-602. PMID: 133443770. . doi: 10.1097/cin.0000000000000459.

48. Jeon E, Park HA. Experiences of Patients With a Diabetes Self-Care App Developed Based on the Information-Motivation-Behavioral Skills Model: Before-and-After Study. JMIR Diabetes. 2019;4(2):e11590. PMID: 30998218. doi: https://dx.doi.org/10.2196/11590.

49. Cady RG. Measuring the Impact of Technology on Nurse Workflow: A Mixed Methods Approach. Minnesota, US: University of Minnesota Digital Conservancy; 2012.

50. Yu CH, Gall Casey C, Ke C, Lebovic G, Straus SE. Process Evaluation of the Diabetes Canada Guidelines Dissemination Strategy Using the Reach Effectiveness Adoption Implementation Maintenance (RE-AIM) Framework. Canadian Journal of Diabetes. 2019 Jun;43(4):263-70. PMID: 30415910. doi: https://dx.doi.org/10.1016/j.jcjd.2018.08.189.

51. Rageliene T, Aschemann-Witzel J, Gronhoj A. Efficacy of a smartphone application-based intervention for encouraging children's healthy eating in Denmark. Health Promot Internation. 2021 Jul 10;10:10. PMID: 34245283. doi: https://dx.doi.org/10.1093/heapro/daab081.

52. Psihogios AM, King-Dowling S, O'Hagan B, Darabos K, Maurer L, Young J, et al. Contextual Predictors of Engagement in a Tailored mHealth Intervention for Adolescent and Young Adult Cancer Survivors. Annals of Behavioral Medicine. 2021;01:01. PMID: 33674863. doi: https://dx.doi.org/10.1093/abm/kaab008.

53. Piera-Jimenez J, Etzelmueller A, Kolovos S, Folkvord F, Lupianez-Villanueva F. Guided Internet-Based Cognitive Behavioral Therapy for Depression: Implementation Cost-Effectiveness Study. Journal of Medical Internet Research. 2021 05 11;23(5):e27410. PMID: 33973857. doi: https://dx.doi.org/10.2196/27410.

54. Nachum S, Gogia K, Clark S, Hsu H, Sharma R, Greenwald PW. An Evaluation of Kiosks for Direct-to-Consumer Telemedicine Using the National Quality Forum Assessment Framework. Telemedicine Journal & E-Health. 2021;27(2):178-83. PMID: 32589518. doi: https://dx.doi.org/10.1089/tmj.2019.0318.

55. McAlearney AS, Hefner JL, Sieck CJ, Huerta TR. The journey through grief: Insights from a qualitative study of electronic health record implementation. References. Health Services Research. 2015;50(2):462-88. doi: http://dx.doi.org/10.1111/1475-6773.12227.

56. Darcy S, Maxwell H, Green J. Disability citizenship and independence through mobile technology? A study exploring adoption and use of a mobile technology platform. Disability & Society. 2016;31(4):497-519. PMID: 116100232. Language: English. Entry Date: 20160617. Revision Date: 20190213. Publication Type: Article. doi: 10.1080/09687599.2016.1179172.

57. Verhoeven F, Steehouder MF, Hendrix RMG, van Gemert-Pijnen JEWC. Factors affecting health care workers' adoption of a website with infection control guidelines. International Journal of Medical Informatics. 2009 October;78(10):663-78. PMID: 50570073. doi: http://dx.doi.org/10.1016/j.ijmedinf.2009.06.001.

58. Laurie J, Blandford A. Making time for mindfulness. International Journal of Medical Informatics. 2016;96:38-50. PMID: 119341958. . doi: 10.1016/j.ijmedinf.2016.02.010.

59. Renard M, Gaboury I, Michaud F, Tousignant M. The acceptability of two remote monitoring modalities for patients waiting for services in a physiotherapy outpatient clinic. Musculoskeletal care. 2022;20(3):616-24. PMID: 35142425. doi: doi: 10.1002/msc.1622.

60. Dharmayat KI, Tran T, Hardy V, Chirambo BG, Thompson MJ, Ide N, et al. Sustainability of 'mHealth' interventions in sub- Saharan Africa: a stakeholder analysis of an electronic community case management project in Malawi. Malawi Medical Journal. 2019 09;31(3):177-83. PMID: 31839886. doi: https://dx.doi.org/10.4314/mmj.v31i3.3.

61. Meeks DW, Takian A, Sittig DF, Singh H, Barber N. Exploring the sociotechnical intersection of patient safety and electronic health record implementation. Journal of the American Medical Informatics Association. 2014;21(e1):e28-34. PMID: 104010867. Language: English. Entry Date: 20140328. Revision Date: 20200708. Publication Type: journal article. doi: 10.1136/amiajnl-2013-001762.

62. Whittaker AA, Aufdenkamp M, Tinley S. Barriers and facilitators to electronic documentation in a rural hospital. Journal of Nursing Scholarship. 2009;41(3):293-300. PMID: 105431347. doi: 10.1111/j.1547-5069.2009.01278.x.

63. Shaw S, Wherton J, Vijayaraghavan S, Morris J, Bhattacharya S, Hanson P, et al. Advantages and limitations of virtual online

consultations in a NHS acute trust: the VOCAL mixed-methods study. 2018 June 2018. Report No.

64. Wang SL, Lin HI. Integrating TTF and IDT to evaluate user intention of big data analytics in mobile cloud healthcare system. Behaviour & Information Technology. 2019;38(9):974-85. PMID: 138138852. . doi: 10.1080/0144929x.2019.1626486.

65. Aboelmaged M, Hashem G. RFID application in patient and medical asset operations management: A technology, organizational and environmental (TOE) perspective into key enablers and impediments. International Journal of Medical Informatics. 2018;118:58-64. PMID: 131430379. . doi: 10.1016/j.ijmedinf.2018.07.009.

66. Levesque DA, Johnson JL, Prochaska JM. Teen Choices, an Online Stage-Based Program for Healthy, Nonviolent Relationships: Development and Feasibility Trial. Journal of School Violence. 2017;16(4):376-85. PMID: 125435850. . doi: 10.1080/15388220.2016.1147964.

67. Gagnon MP, Orruno E, Asua J, Abdeljelil AB, Emparanza J. Using a modified technology acceptance model to evaluate healthcare professionals' adoption of a new telemonitoring system. Telemedicine journal and e-health. 2012 Jan-Feb;18(1):54-9. PMID: 22082108. doi: 10.1089/tmj.2011.0066.

68. Lieshout Fv, Yang R, Stamenova V, Agarwal P, Palma DC, Sidhu A, et al. Evaluating the Implementation of a Remote-Monitoring Program for Chronic Obstructive Pulmonary Disease: Qualitative Methods from a Service Design Perspective. Journal of Medical Internet Research. 2020;22(10). PMID: 146783865. . doi: 10.2196/18148.

69. Vasalampi A. Adoption and Use of a Mobile System at Home Care. Studies in Health Technology & Informatics. 2017;242:1042-6. PMID: 134729642. . doi: 10.3233/978-1-61499-798-6-1042.

70. Shaw RJ, Kaufman MA, Bosworth HB, Weiner BJ, Zullig LL, Lee SYD, et al. Organizational factors associated with readiness to implement and translate a primary care based telemedicine behavioral program to improve blood pressure control: the HTN-IMPROVE study. Implementation Science. 2013;8(1):106-. PMID: 104115546. . doi: 10.1186/1748-5908-8-106.
