# Supplementary material for: Mapping Theories, Models, and Frameworks to Evaluate Digital Health Interventions: Scoping Review
Source: J Med Internet Res. 2024 Feb 5;26:e51098. doi: 10.2196/51098 (PMC10877497; doi:10.2196/51098)
Supplement: Multimedia Appendix 6 [file jmir_v26i1e51098_app6.docx]

**Multimedia Appendix 6**. Descriptive table of most reported theories, models, and frameworks (TMFs)

**Consolidated Framework for Implementation Research (CFIR)**

|  | **DHI user** | **Study Type** | **TMF used in isolation or combination?** | **Role(s) of framework** |
| --- | --- | --- | --- | --- |
| Bandini, A., et al. (2021). "Perspectives and recommendations of individuals with tetraplegia regarding wearable cameras for monitoring hand function at home: Insights from a community-based study." The journal of spinal cord medicine 44(sup1): S173-S184. | 1.0 Clients | Mixed-Methods | Isolation | 2. To inform data collection  9. To specify outcomes |
| Bardosh, K. L., et al. (2017). "Operationalizing mHealth to improve patient care: A qualitative implementation science evaluation of the WelTel texting intervention in Canada and Kenya." Globalization and Health 13(1). | 2.0 Healthcare Providers | Qualitative | Isolation | 1. To identify key constructs that may serve as barriers and facilitators  2. To inform data collection  3. To guide implementation planning 8. To guide the selection of implementation strategies |
| Barker, L. T., et al. (2020). "A novel in situ simulation framework for introduction of a new technology: the 3-Act-3-Debrief model." Advances in Simulation 5: 25. | 2.0 Healthcare Providers | Quantitative | Isolation | 1. To identify key constructs that may serve as barriers and facilitators 2. To inform data collection  3. To guide implementation planning  8. To guide the selection of implementation strategies |
| Bastos de Carvalho, A., et al. (2021). "Evaluation of multi-level barriers and facilitators in a large diabetic retinopathy screening program in federally qualified health centers: a qualitative study." Implementation Science Communications 2(1): 54. | 1.0 Clients | Qualitative | Isolation | 1. To identify key constructs that may serve as barriers and facilitators 7. To inform data analysis |
| Batsis, J. A., et al. (2020). "Barriers and facilitators in implementing a pilot, pragmatic, telemedicine-delivered healthy lifestyle program for obesity management in a rural, academic obesity clinic." Implementation Science Communications 1: 83. | 1.0 Clients | Mixed-Methods | RE-AIM | 1. To identify key constructs that may serve as barriers and facilitator 2. To inform data collection  7. To inform data analysis |
| Black, W. E., et al. (2020). "Leveraging Health Information Technology to Meet The Joint Commission's Standard for Measurement-Based Care: A Case Study." Joint Commission Journal on Quality & Patient Safety 46(6): 353-358. | 1.0 Clients | Quantitative | Isolation | 1. To identify key constructs that may serve as barriers and facilitators 5. To specify the process of implementation  6. To frame an evaluation |
| Boet, S., et al. (2021). "Implementation of the Operating Room Black Box Research Program at the Ottawa Hospital Through Patient, Clinical, and Organizational Engagement: Case Study." Journal of Medical Internet Research 23(3): e15443. | 2.0 Healthcare Providers | Qualitative | Isolation | 1. To identify key constructs that may serve as barriers and facilitators 2. To inform data collection  7. To inform data analysis |
| Brunet, N., et al. (2020). "Increasing buprenorphine access for veterans with opioid use disorder in rural clinics using telemedicine." Substance Abuse(Pagination). | 1.0 Clients | Qualitative | Isolation | 1. To identify key constructs that may serve as barriers and facilitators 7. To inform data analysis |
| Busse, T. S., et al. (2022). "Planning for Implementation Success of an Electronic Cross-Facility Health Record for Pediatric Palliative Care Using the Consolidated Framework for Implementation Research (CFIR)." International Journal of Environmental Research and Public Health 19(1). | 2.0 Healthcare Providers | Qualitative | Isolation | 1. To identify key constructs that may serve as barriers and facilitators 3. To guide implementation planning 7. To inform data analysis |
| Christie, H. L., et al. (2020). "Developing a Plan for the Sustainable Implementation of an Electronic Health Intervention (Partner in Balance) to Support Caregivers of People With Dementia: Case Study." 24058297 3(1): e18624. | 1.0 Clients | Mixed-Methods | Isolation | 2. To inform data collection |
| Cohn, W. F., et al. (2021). "An Implementation Strategy to Expand Mobile Health Use in HIV Care Settings: Rapid Evaluation Study Using the Consolidated Framework for Implementation Research." JMIR mHealth and uHealth 9(4): e19163. | 2.0 Healthcare Providers | Qualitative | Isolation | 1. To identify key constructs that may serve as barriers and facilitators 2. To inform data collection  3. To guide implementation planning 7. To inform data analysis  9. To specify outcomes |
| Crane, M. E., et al. (2021). "A Qualitative Examination of a School-Based Implementation of Computer-Assisted Cognitive-Behavioral Therapy for Child Anxiety." School Mental Health 13(2): 347-361. | 2.0 Healthcare Providers | Qualitative | Isolation | 1. To identify key constructs that may serve as barriers and facilitators 7. To inform data analysis 8. To guide the selection of implementation strategies |
| Damschroder, L. J., et al. (2017). "Implementation evaluation of the Telephone Lifestyle Coaching (TIC) program: Organizational factors associated with successful implementation. References." Translational Behavioral Medicine 7(2): 233-241. | 1.0 Clients | Mixed-Methods | Isolation | 2. To inform data collection  6. To frame an evaluation 7. To inform data analysis |
| El Joueidi, S., et al. (2021). "Evaluation of the implementation process of the mobile health platform 'WelTel' in six sites in East Africa and Canada using the modified consolidated framework for implementation research (mCFIR)." BMC Medical Informatics and Decision Making 21(1): 293. | 1.0 Clients 2.0 Healthcare Providers | Mixed-Methods | Isolation | 1. to identify key constructs that may serve as barriers and facilitators 7. to inform data analysis |
| Fehrenbacher, C., et al. (2020). "Referral to digital parent training in primary care: Facilitators and barriers. References." Clinical Practice in Pediatric Psychology 8(3): 268-277. | 2.0 Healthcare Providers | Qualitative | Isolation | 1. To identify key constructs that may serve as barriers and facilitators 2. To inform data collection  7. To inform data analysis |
| Garg, S. K., et al. (2016). "Qualitative analysis of programmatic initiatives to text patients with mobile devices in resource-limited health systems." BMC Medical Informatics & Decision Making: 1-12. | 2.0 Healthcare Providers | Qualitative | Isolation | 1. To identify key constructs that may serve as barriers and facilitators 2. To inform data collection  7. To inform data analysis |
| Hadjistavropoulos, H. D., et al. (2017). "Implementation of Internet-delivered cognitive behavior therapy within community mental health clinics: A process evaluation using the consolidated framework for implementation research. References." BMC Psychiatry 17: 331. | 1.0 Clients | Mixed-Methods | Isolation | 1. To identify key constructs that may serve as barriers and facilitators 2. To inform data collection  7. To inform data analysis |
| Harry, M. L., et al. (2019). "Barriers and facilitators to implementing cancer prevention clinical decision support in primary care: a qualitative study." BMC Health Services Research 19(1): N.PAG-N.PAG. | 2.0 Healthcare Providers | Qualitative | Isolation | 1. To identify key constructs that may serve as barriers and facilitators 2. To inform data collection  7. To inform data analysis |
| Haverhals, L. M., et al. (2015). "E-Consult Implementation: Lessons Learned Using Consolidated Framework for Implementation Research." American Journal of Managed Care 21(12): 294-301. | 2.0 Healthcare Providers | Mixed-Methods | Isolation | 1. To identify key constructs that may serve as barriers and facilitators 2. To inform data collection  7. To inform data analysis 9. To specify outcomes |
| Knox, M., et al. (2020). "e-Consult Implementation Success: Lessons From 5 County-Based Delivery Systems." American Journal of Managed Care 26(1): e21-e27. | 2.0 Healthcare Providers | Mixed-Methods | Isolation | 7. To inform data analysis |
| Kummer, B. R., et al. (2021). "Teleneurology Expansion in Response to the COVID-19 Outbreak at a Tertiary Health System in New York City." Neurology Clinical Practice 11(2): e102-e111. | 1.0 Clients | Mixed-Methods | Isolation | 1. To identify key constructs that may serve as barriers and facilitators 7. To inform data analysis |
| Lambert-Kerzner, A. C., et al. (2019). "Use of the consolidated framework for implementation research to guide dissemination and implementation of new technologies in surgery." Journal of Thoracic Disease 11(Supplement4): S487-S499. | 1.0 Clients | Qualitative | Isolation | 1. To identify key constructs that may serve as barriers and facilitators 3. To guide implementation planning 7. To inform data analysis |
| Lamontagne, M. E., et al. (2019). "Implementation Evaluation of an Online Peer-Mentor Training Program for Individuals With Spinal Cord Injury." Topics in Spinal Cord Injury Rehabilitation 25(4): 303-315. | 1.0 Clients | Mixed-Methods | Isolation | 1. To identify key constructs that may serve as barriers and facilitators 2. To inform data collection |
| Levinson, A. J., et al. (2020). "Barriers and Facilitators to Implementing Web-Based Dementia Caregiver Education From the Clinician's Perspective: Qualitative Study." 24058297 3(2): e21264. | 2.0 Healthcare Providers | Qualitative | Isolation | 1. To identify key constructs that may serve as barriers and facilitators 3. To guide implementation planning, 7. To inform data analysis 9. To specify outcomes |
| Lord, S., et al. (2016). "Implementation of a Substance Use Recovery Support Mobile Phone App in Community Settings: Qualitative Study of Clinician and Staff Perspectives of Facilitators and Barriers." JMIR Mental Health 3(2): e24. | 1.0 Clients | Qualitative | Isolation | 1. To identify key constructs that may serve as barriers and facilitators 9. To specify outcomes |
| McCreesh-Toselli, S., et al. (2021). "Staff Perceptions of Preimplementation Barriers and Facilitators to a Mobile Health Antiretroviral Therapy Adherence Counseling Intervention in South Africa: Qualitative Study." JMIR mHealth and uHealth 9(4): e23280. | 2.0 Healthcare Providers | Qualitative | Isolation | 1. To identify key constructs that may serve as barriers and facilitators 2. To inform data collection  7. To inform data analysis |
| Meijer, E., et al. (2021). ""At least someone thinks I'm doing well": a real-world evaluation of the quit-smoking app StopCoach for lower socio-economic status smokers." Addiction science & clinical practice 16(1): 48. | 2.0 Healthcare Providers | Mixed-Methods | Unified Theory of Acceptance and Use of Technology (UTAUT) | 7. To inform data analysis |
| Meyer, A. J., et al. (2020). "Implementing mHealth Interventions in a Resource-Constrained Setting: Case Study From Uganda." JMIR mHealth and uHealth 8(7): e19552. | 2.0 Healthcare Providers | Qualitative | Isolation | 9. To specify outcomes |
| Paulsen, M. M., et al. (2019). "Barriers and Facilitators for Implementing a Decision Support System to Prevent and Treat Disease-Related Malnutrition in a Hospital Setting: Qualitative Study." JMIR Formative Research 3(2): e11890. | 1.0 Clients 2.0 Healthcare Providers | Qualitative | Isolation | 2. To inform data collection  7. To inform data analysis |
| Robins, L. S., et al. (2013). "Barriers and Facilitators to Evidence-based Blood Pressure Control in Community Practice." Journal of the American Board of Family Medicine 26(5): 539-557. | 2.0 Healthcare Providers | Qualitative | Chronic Care Model | 1. To identify key constructs that may serve as barriers and facilitators 7. To inform data analysis 9. To specify outcomes, |
| Rogers, E., et al. (2019). "Barriers and Facilitators to the Implementation of a Mobile Insulin Titration Intervention for Patients With Uncontrolled Diabetes: A Qualitative Analysis." JMIR mHealth and uHealth 7(7): e13906. | 1.0 Clients 2.0 Healthcare Providers | Qualitative | Isolation | 1. To identify key constructs that may serve as barriers and facilitators 2. To inform data collection 7. To inform data analysis 9. To specify outcomes |
| Seljelid, B., et al. (2021). "A Digital Patient-Provider Communication Intervention (InvolveMe): Qualitative Study on the Implementation Preparation Based on Identified Facilitators and Barriers." Journal of Medical Internet Research 23(4): e22399. | 1.0 Clients | Qualitative | Isolation | 1. To identify key constructs that may serve as barriers and facilitators 2. To inform data collection  3. To guide implementation planning 7. To inform data analysis 9. To specify outcomes |
| Shea, C. M., et al. (2018). "Telestroke Adoption Among Community Hospitals in North Carolina: A Cross-Sectional Study." Journal of Stroke & Cerebrovascular Diseases 27(9): 2411-2417. | 3.0 Health System Managers | Quantitative | Isolation | 1. To identify key constructs that may serve as barriers and facilitators 2. To inform data collection  7. To inform data analysis 9. To specify outcomes |
| Stevenson, L., et al. (2018). "Evaluation of a national telemedicine initiative in the Veterans Health Administration: Factors associated with successful implementation." Journal of Telemedicine & Telecare 24(3): 168-178. | 2.0 Healthcare Providers | Mixed-Methods | Isolation | 1. To identify key constructs that may serve as barriers and facilitators 2. To inform data collection 7. To inform data analysis 9. To specify outcomes, |
| Varsi, C., et al. (2015). "Using the Consolidated Framework for Implementation Research to Identify Barriers and Facilitators for the Implementation of an Internet-Based Patient-Provider Communication Service in Five Settings: A Qualitative Study." Journal of Medical Internet Research 17(11): 1-1. | 1.0 Clients 2.0 Healthcare Providers | Qualitative | Isolation | 1. To identify key constructs that may serve as barriers and facilitators 2. To inform data collection 7. To inform data analysis  9. To specify outcomes, |
| Ware, P., et al. (2018). "Evaluating the Implementation of a Mobile Phone-Based Telemonitoring Program: Longitudinal Study Guided by the Consolidated Framework for Implementation Research." JMIR mHealth and uHealth 6(7): e10768. | 1.0 Clients 2.0 Healthcare Providers | Qualitative | Implementation Outcomes Framework | 1. To identify key constructs that may serve as barriers and facilitators 2. To inform data collection  7. To inform data analysis 9. To specify outcomes |
| Warner, G., et al. (2018). "Applying the consolidated framework for implementation research to identify barriers affecting implementation of an online frailty tool into primary health care: a qualitative study." BMC Health Services Research 18(1): 395. | 2.0 Healthcare Providers | Qualitative | Isolation | 1. To identify key constructs that may serve as barriers and facilitators 2. To inform data collection 7. To inform data analysis,  9. To specify outcomes |
| Williams, K. M., et al. (2017). "Evaluation of the Veterans Health Administration's Specialty Care Transformational Initiatives to Promote Patient-Centered Delivery of Specialty Care: A Mixed-Methods Approach." Telemedicine Journal & E-Health 23(7): 577-589. | 2.0 Healthcare Providers | Mixed-Methods | RE-AIM | 1. To identify key constructs that may serve as barriers and facilitators 2. To inform data collection  7. To inform data analysis 9. To specify outcomes |
| Adeoye-Olatunde, O. A., et al. (2022). "Preparing for the spread of patient-reported outcome (PRO) data collection from primary care to community pharmacy: a mixed-methods study." Implementation Science Communications 3(1): 29. | 3.0 Health System Managers | Mixed-Methods | Curran et al.’s approach to Evidence-Based Quality  Expert Recommendations for Implementing Change | 2. To inform data collection  7. To inform data analysis |

**Reach, Effectiveness, Adoption, Implementation, and Maintenance (RE-AIM)**

| **Author** | **DHI user** | **Study Type** | **TMF used in isolation or combination?** | **Role(s) of framework** |
| --- | --- | --- | --- | --- |
| Beukes, E. W., et al. (2018). "Process evaluation of Internet-based cognitive behavioural therapy for adults with tinnitus in the context of a randomised control trial." International Journal of Audiology 57(2): 98-109. | 1.0 Clients | Quantitative | Isolation | 1. To identify key constructs that may serve as barriers and facilitators  2. To inform data collection  7. To inform data analysis |
| Hale-Gallardo, J. L., et al. (2020). "Telerehabilitation for Rural Veterans: A Qualitative Assessment of Barriers and Facilitators to Implementation." Journal of Multidisciplinary Healthcare 13: 559-570. | 1.0 Clients | Qualitative | Isolation | 1. To identify key constructs that may serve as barriers and facilitators  2. To inform data collection  7. To inform data analysis  9. To specify outcomes |
| Johnson, N. L., et al. (2020). "Engaging Parents in Education for Discharge (ePED): Evaluating the Reach, Adoption & Implementation of an Innovative Discharge Teaching Method." Journal of Pediatric Nursing 54: 42-49. | 1.0 Clients | Mixed-Methods | Isolation | 2. To inform data collection  6. To frame an evaluation  7. To inform data analysis  9. To specify outcomes |
| Koot, D., et al. (2019). "A Mobile Lifestyle Management Program (GlycoLeap) for People With Type 2 Diabetes: Single-Arm Feasibility Study." JMIR mHealth and uHealth 7(5): e12965. | 1.0 Clients | Quantitative | Isolation | 2. To inform data collection  3. To guide implementation planning,  6. To frame an evaluation  7. To inform data analysis  9. To specify outcomes |
| Liddy, C., et al. (2019). "Supporting the spread and scale-up of electronic consultation across Canada: cross-sectional analysis." BMJ Open 9(5): e028888. | 2.0 Healthcare Providers | Qualitative | Isolation | 2. To inform data collection  3. To guide implementation planning  6. To frame an evaluation  7. To inform data analysis  9. To specify outcomes |
| Palermo, T. M., et al. (2020). "A digital health psychological intervention (WebMAP Mobile) for children and adolescents with chronic pain: results of a hybrid effectiveness-implementation stepped-wedge cluster randomized trial." Pain (03043959) **161**(12): 2763-2774. | 1.0 Clients 2.0 Healthcare Providers | Mixed-Methods | Isolation | 8. To guide the selection of implementation strategies |
| Peels, D. A., et al. (2012). "Development of Web-based computer-tailored advice to promote physical activity among people older than 50 years. References." Journal of Medical Internet Research 14(2): 15-27. | 1.0 Clients | Quantitative | Isolation | 6. To frame an evaluation  9. To specify outcomes |
| Peracca, S. B., et al. (2021). "Implementing Teledermatology for Rural Veterans: An Evaluation Using the RE-AIM Framework." Telemedicine Journal & E-Health 27(2): 218-226. | 2.0 Healthcare Providers | Quantitative | Isolation | 2. To inform data collection  7. To inform data analysis |
| Schultz, K., et al. (2021). "Implementation of a virtual ward as a response to the COVID-19 pandemic." Australian Health Review 45(4): 433-441. | 1.0 Clients | Quantitative | Non-adoption, Abandonment, Scale-up, Spread, Sustainability (NASSS) framework  Consolidated Framework for Implementation Research (CFIR) | 2. To inform data collection |
| Spaulding, A., et al. (2019). "Qualitative study of implementation of patient self‐reported measures in a consultation‐liaison psychiatry practice." Journal of Evaluation in Clinical Practice 25(3): 482-490. | 1.0 Clients 2.0 Healthcare Providers | Mixed-Methods | Isolation | 1. To identify key constructs that may serve as barriers and facilitators  2. To inform data collection  6. To frame an evaluation  7. To inform data analysis  9. To specify outcomes |
| van den Berg, M. H., et al. (2008). "Implementation of a physical activity intervention for people with rheumatoid arthritis: a case study." Musculoskeletal Care 6(2): 69-85. | 1.0 Clients | Mixed-Methods | Isolation | 1. To identify key constructs that may serve as barriers and facilitators  2. To inform data collection  7. To inform data analysis  9. To specify outcomes |
| Vriend, I., et al. (2015). "Implementation of an App-based neuromuscular training programme to prevent ankle sprains: a process evaluation using the RE-AIM Framework." British Journal of Sports Medicine 49(7): 484-488. | 1.0 Clients | Mixed-Methods | Isolation | 2. To inform data collection  6. To frame an evaluation  7. To inform data analysis  9. To specify outcomes |
| Yu, C. H., et al. (2019). "Process Evaluation of the Diabetes Canada Guidelines Dissemination Strategy Using the Reach Effectiveness Adoption Implementation Maintenance (RE-AIM) Framework." Canadian Journal of Diabetes 43(4): 263-270.e269. | 1.0 Clients 2.0 Healthcare Providers | Qualitative | Knowledge-to-Action framework | 2. To inform data collection  6. To frame an evaluation  7. To inform data analysis  9. To specify outcomes |
| Gong, E., et al. (2021). "The Implementation of a Primary Care-Based Integrated Mobile Health Intervention for Stroke Management in Rural China: Mixed-Methods Process Evaluation." Frontiers in Public Health **9**: 774907. | 1.0 Clients 2.0 Healthcare Providers | Mixed-Methods | Isolation | 6. To frame an evaluation  9. To specify outcomes |
| Batsis, J. A., et al. (2020). "Barriers and facilitators in implementing a pilot, pragmatic, telemedicine-delivered healthy lifestyle program for obesity management in a rural, academic obesity clinic." Implementation Science Communications 1: 83. | 1.0 Clients | Quantitative | CFIR | 1. To identify key constructs that may serve as barriers and facilitators  2. To inform data collection  7. To inform data analysis |
| Williams, K. M., et al. (2017). "Evaluation of the Veterans Health Administration's Specialty Care Transformational Initiatives to Promote Patient-Centered Delivery of Specialty Care: A Mixed-Methods Approach." Telemedicine Journal & E-Health 23(7): 577-589. | 2.0 Healthcare Providers | Mixed-Methods | CFIR | 2. To inform data collection  6. To frame an evaluation  7. To inform data analysis  9. To specify outcomes |
| Pelletier, A. C., et al. (2011). "Implementing a web-based home monitoring system within an academic health care network: Barriers and facilitators to innovation diffusion." Journal of Diabetes Science and Technology 5(1): 32-38. | 1.0 Clients 2.0 Healthcare Providers | Mixed-Methods | Roger's Influential Diffusion of Innovation (DOI) Theory | 9. To specify outcomes |

**Technology Acceptance Model (TAM)**

| **Author** | **DHI user** | **Study Type** | **TMF used in isolation or combination?** | **Role(s) of framework** |
| --- | --- | --- | --- | --- |
| Akdur, G., et al. (2020). "Adoption of Mobile Health Apps in Dietetic Practice: Case Study of Diyetkolik." JMIR mHealth and uHealth 8(10): e16911. | 1.0 Clients | Quantitative | Isolation | 2. To inform data collection  7. To inform data analysis |
| Alvarado, N., et al. (2021). "Analysis of a Web-Based Dashboard to Support the Use of National Audit Data in Quality Improvement: Realist Evaluation." Journal of Medical Internet Research 23(11): e28854. | 3.0 Health System Managers | Mixed-Methods | Isolation | 2. To inform data collection  7. To inform data analysis 9. To specify outcomes |
| Catchpole, K., et al. (2021). "A Smartphone Application for Teamwork and Communication in Trauma: Pilot Evaluation "in the Wild"." Human Factors: 187208211021717. | 2.0 Healthcare Providers | Mixed-Methods | Isolation | 1. To identify key constructs that may serve as barriers and facilitators 7. To inform data analysis |
| Croff, R. L., et al. (2019). "Things Are Changing so Fast: Integrative Technology for Preserving Cognitive Health and Community History." Gerontologist **59**(1): 147-157. | 1.0 Clients | Mixed-Methods | Isolation | 7. To inform data analysis |
| Day, M., et al. (2007). "Exploring underutilization of videophones in hospice settings." Telemedicine Journal and e-Health **13**(1): 25-31. | 2.0 Healthcare Providers | Qualitative | Isolation | 2. To inform data collection  7. To inform data analysis |
| Ehteshami A. (2017). Barcode Technology Acceptance and Utilization in Health Information Management Department at Academic Hospitals According to Technology Acceptance Model. Acta Inform Med. **25**(1):4-8. | 2.0 Healthcare Providers | Quantitative | Isolation | 2. To inform data collection  7. To inform data analysis |
| Kinshella, M.-L. W., et al. (2021). ""Now You Have Become Doctors": Lady Health Workers' Experiences Implementing an mHealth Application in Rural Pakistan." Frontiers in global women's health 2: 645705. | 2.0 Healthcare Providers | Qualitative | Isolation | 2. To inform data collection |
| Liu, M. C. and C. C. Lee (2018). "An Investigation of Pharmacists’ Acceptance of NHI-PharmaCloud in Taiwan." Journal of Medical Systems 42(11): 1-1. | 2.0 Healthcare Providers | Quantitative | Self-efficacy within the healthcare system  Perceived risk and pharmacists’ behaviour | 2. To inform data collection 9. To specify outcome |
| Ljubicic, V., et al. (2020). "Drivers of intentions to use healthcare information systems among health and care professionals." Health Informatics Journal 26(1): 56-71. | 2.0 Healthcare Providers | Quantitative | Unified theory of acceptance and use of technology (UTAUT) | 2. To inform data collection  9. To specify outcomes |
| Mei, Y. Y., et al. (2013). "Designing and evaluating an electronic patient falls reporting system: Perspectives for the implementation of health information technology in long-term residential care facilities." International Journal of Medical Informatics 82(11): e294-306. | 4.0 Data Services | Mixed-Methods | Holistic human factors evaluation | 2. To inform data collection  6. To frame an evaluation |
| Nápoles, A. M., et al. (2016). "Perceptions of clinicians and staff about the use of digital technology in primary care: qualitative interviews prior to implementation of a computer-facilitated 5As intervention." BMC Medical Informatics & Decision Making 16: 1-13. | 1.0 Clients 2.0 Healthcare Providers | Qualitative | Isolation | 2. To inform data collection  9. To specify outcomes |
| Ndlovu, K., et al. (2022). "Acceptance of the District Health Information System Version 2 Platform for Malaria Case-Based Surveillance By Health Care Workers in Botswana: Web-Based Survey." JMIR Formative Research **6**(3): e32722. | 3.0 Health System Managers | Mixed-methods | Isolation | 1. To identify key constructs that may serve as barriers and facilitators 2. To inform data collection |
| Pérez-Rodríguez, R., et al. (2020). "FriWalk robotic walker: usability, acceptance and UX evaluation after a pilot study in a real environment." Disability & Rehabilitation: Assistive Technology 15(6): 718-727. | 1.0 Clients | Mixed-Methods | Isolation | 2. To inform data collection |
| van der Kamp, M., et al. (2021). "Feasibility, Efficacy, and Efficiency of eHealth-Supported Pediatric Asthma Care: Six-Month Quasi-Experimental Single-Arm Pretest-Posttest Study." JMIR Formative Research 5(7): e24634. | 2.0 Healthcare Providers | Mixed-Methods | Isolation | 2. To inform data collection |
| Ebenso, B., et al. (2021). "What Are the Contextual Enablers and Impacts of Using Digital Technology to Extend Maternal and Child Health Services to Rural Areas? Findings of a Qualitative Study From Nigeria." Frontiers in global women's health 2: 670494. | 2.0 Healthcare Providers | Mixed-Methods | Isolation | 1. to identify key constructs that may serve as barriers and facilitators  2. To inform data collection  7. to inform data analysis 9. To specify outcomes |
| Watkinson, F., et al. (2021). "A mixed-method service evaluation of health information exchange in England: technology acceptance and barriers and facilitators to adoption." BMC Health Services Research 21(1): 737. | 2.0 Healthcare Providers | Mixed-Methods | Unified Theory of Acceptance and Use of Technology (UTAUT)  Normalisation Process Theory (NPT) | 2. To inform data collection |

**Unified Theory of Acceptance and Use of Technology (UTAUT)**

| **Author** | **DHI user** | **Study Type** | **TMF used in isolation or combination?** | **Role(s) of framework** |
| --- | --- | --- | --- | --- |
| Chiu, T. M. L. and G. Eysenbach (2010). "Stages of use: consideration, initiation, utilization, and outcomes of an internet-mediated intervention." BMC Medical Informatics & Decision Making 10(1): 73-73. | 2.0 Healthcare Providers | Quantitative | Behavioral Model of Health Service Use (BMHSU) | 2. To inform data collection |
| den Bakker Cm, H. J. A. S. F. G. d. G. C. B. H. J. A. J. R. (2019). "Electronic Health Program to Empower Patients in Returning to Normal Activities After Colorectal Surgical Procedures: mixed-Methods Process Evaluation Alongside a Randomized Controlled Trial." Journal of Medical Internet Research 21(1): e10674. | 1.0 Clients | Mixed-Methods | Isolation | 6. To frame an evaluation  7. To inform data analysis |
| Geerds, M. A. J., et al. (2020). "Mobile App for Monitoring 3-Month Postoperative Functional Outcome After Hip Fracture: Usability Study." JMIR Human Factors 7(3): e16989. | 1.0 Clients | Quantitative | Isolation | 2. To inform data collection |
| Jaana, M., et al. (2019). "A prospective evaluation of telemonitoring use by seniors with chronic heart failure: Adoption, self-care, and empowerment." Health Informatics Journal 25(4): 1800-1814. | 1.0 Clients | Mixed-Methods | Isolation | 1. To identify key constructs that may serve as barriers and facilitators  2. To inform data collection  7. To inform data analysis 9. To specify outcomes |
| Lalitaphanit, K. and A. Theeraroungchaisri (2016). "Factors affecting community pharmacy customers' decision to use personal health records via smartphone." Thai Journal of Pharmaceutical Sciences 40: 163-167. | 1.0 Clients 2.0 Healthcare Providers | Quantitative | Isolation | 1. To identify key constructs that may serve as barriers and facilitators 9. To specify outcomes |
| Ngo, V., et al. (2020). "The Personal Health Network Mobile App for Chemotherapy Care Coordination: Qualitative Evaluation of a Randomized Clinical Trial." JMIR mHealth and uHealth 8(5): e16527. | 1.0 Clients | Qualitative | Isolation | 2. To inform data collection |
| Sheshadri, A., et al. (2022). "Home-Based Spirometry Telemonitoring After Allogeneic Hematopoietic Cell Transplantation: Mixed Methods Evaluation of Acceptability and Usability." JMIR Formative Research 6(2): e29393. | 2.0 Healthcare Providers | Mixed-Methods | Isolation | 1. To identify key constructs that may serve as barriers and facilitators 2. To inform data collection  8. To guide the selection of implementation strategies |
| Watkinson, F., et al. (2021). "A mixed-method service evaluation of health information exchange in England: technology acceptance and barriers and facilitators to adoption." BMC Health Services Research **21**(1): 737. | 2.0 Healthcare Providers | Mixed-Methods | TAM  Normalisation Process Theory (NPT) | 2. To inform data collection |
| Vuorinen, M. (2020). "Registered Nurses' experiences with, and feelings and attitudes towards, the International Resident Assessment Instrument for Long-Term Care Facilities in New Zealand in 2017." Journal of Research in Nursing 25(2): 141-155. | 2.0 Healthcare Providers | Qualitative | Isolation | 1. To identify key constructs that may serve as barriers and facilitators  2. To inform data collection |
| Meijer, E., et al. (2021). ""At least someone thinks I'm doing well": a real-world evaluation of the quit-smoking app StopCoach for lower socio-economic status smokers." Addiction science & clinical practice 16(1): 48. | 2.0 Healthcare Providers | Mixed-Methods | CFIR | 7. To inform data analysis |
| Ljubicic, V., et al. (2020). "Drivers of intentions to use healthcare information systems among health and care professionals." Health Informatics Journal 26(1): 56-71. | 2.0 Healthcare Providers | Quantitative | Roger's Influential Diffusion of Innovation (DOI) Theory  Technology Acceptance Model (TAM) | 2. To inform data collection  9. To specify outcomes |
| Haque, M. S., et al. (2020). "A persuasive mhealth behavioral change intervention for promoting physical activity in the workplace: Feasibility randomized controlled trial." Journal of Medical Internet Research **4**(5). | 1.0 Clients | Mixed-Methods | Self-determination theory | 2. To inform data collection |

**Normalisation Process Theory (NPT)**

| **Author** | **DHI user** | **Study Type** | **TMF used in isolation or combination?** | **Role(s) of framework** |
| --- | --- | --- | --- | --- |
| Farr, M., et al. (2018). "Implementing online consultations in primary care: A mixed-method evaluation extending normalisation process theory through service co-production." BMJ Open 8(3). | 2.0 Healthcare Providers | Mixed-Methods | Isolation | 2. To inform data collection  3. To guide implementation planning 7. To inform data analysis 8. To guide the selection of implementation strategies |
| Farr, M., et al. (2019). "Pilot implementation of co-designed software for co-production in mental health care planning: a qualitative evaluation of staff perspectives." Journal of Mental Health 28(5): 495-504. | 2.0 Healthcare Providers | Qualitative | Isolation | 1. To identify key constructs that may serve as barriers and facilitators  2. To inform data collection 7. To inform data analysis |
| Jones, C. H. D., et al. (2016). "Embedding new technologies in practice - a normalization process theory study of point of care testing." BMC Health Services Research **16**: 591-591. | 2.0 Healthcare Providers | Mixed-Methods | Isolation | 2. To inform data collection  6. To frame an evaluation  7. To inform data analysis |
| Lennon, M. R., et al. (2017). "Readiness for Delivering Digital Health at Scale: Lessons From a Longitudinal Qualitative Evaluation of a National Digital Health Innovation Program in the United Kingdom." Journal of Medical Internet Research **19**(2): 1-1. | 1.0 Clients | Qualitative | Isolation | 1. To identify key constructs that may serve as barriers and facilitators  2. To inform data collection  6. To frame an evaluation 7. To inform data analysis |
| Mishuris Rg, P. J. M. L. H. R. F. D. S. P. D. M. T. M. D. M. (2017). "Using normalization process theory to understand work flow implications of decision support implementation across diverse primary care settings." Journal of General Internal Medicine 32(2): S370-. | 2.0 Healthcare Providers | Quantitative | Isolation | 2. To inform data collection  9. To specify outcomes |
| Myall, M., et al. (2015). "RESTORE: an exploratory trial of a web-based intervention to enhance self-management of cancer-related fatigue: findings from a qualitative process evaluation." BMC Medical Informatics & Decision Making 15: 1-9. | 1.0 Clients | Qualitative | Isolation | 2. To inform data collection  9. To specify outcomes |
| Steele Gray C., et al. (2021). "Assessing the Implementation and Effectiveness of the Electronic Patient-Reported Outcome Tool for Older Adults With Complex Care Needs: mixed Methods Study." Journal of Medical Internet Research 23(12): e29071. | 1.0 Clients | Mixed-Methods | Combination*  (Combined with Medical Research Council guideline, which was excluded) | 7. To inform data analysis 9. To specify outcomes |
| Webb, M. J., et al. (2018). "Experiences of General Practitioners and Practice Support Staff Using a Health and Lifestyle Screening App in Primary Health Care: Implementation Case Study." JMIR mHealth and uHealth **6**(4): e105. | 1.0 Clients  2.0 Healthcare Providers | Qualitative | Plan-Do-Study-Act (PDSA) | 2. To inform data collection 7. To inform data analysis 9. To specify outcomes |
| Watkinson, F., et al. (2021). "A mixed-method service evaluation of health information exchange in England: technology acceptance and barriers and facilitators to adoption." BMC Health Services Research **21**(1): 737. | 2.0 Healthcare Providers | Mixed-Methods | UTAUT  TAM | 2. To inform data collection |

**Diffusion of Innovation (DOI) Theory**

| **Author** | **DHI user** | **Study Type** | **TMF used in isolation or combination?** | **Role(s) of framework** |
| --- | --- | --- | --- | --- |
| Carper, M. M., et al. (2013). "The dissemination of computer-based psychological treatment: A preliminary analysis of patient and clinician perceptions. References." Administration and Policy in Mental Health and Mental Health Services Research 40(2): 87-95. | 1.0 Clients | Quantitative | Isolation | 2. To inform data collection  4. To enhance conceptual clarity 6. To frame an evaluation 10. To clarify terminology |
| Emani, S., et al. (2018). "Who adopts a patient portal?: An application of the diffusion of innovation model." Journal of Innovation in Health Informatics **25**(3): 149-157. | 1.0 Clients | Quantitative | Isolation | 9. To specify outcomes |
| Haun, M. W., et al. (2020). "Intent to adopt video-based integrated mental health care and the characteristics of its supporters: Mixed methods study among general practitioners applying diffusion of innovations theory." JMIR Mental Health 7(10). | 2.0 Healthcare Providers | Mixed-Methods | Isolation | 1. To identify key constructs that may serve as barriers and facilitators 7. To inform data analysis |
| Helitzer, D., et al. (2003). "Assessing or Predicting Adoption of Telehealth Using the Diffusion of Innovations Theory: A Practical Example from a Rural Program in New Mexico. References." Telemedicine Journal and e Health **9**(2): 179-187. | 1.0 Clients | Qualitative | Isolation | 1. To identify key constructs that may serve as barriers and facilitators 7. To inform data analysis |
| Knoerl, R., et al. (2017). "Pilot Testing a Web-Based System for the Assessment and Management of Chemotherapy-Induced Peripheral Neuropathy." CIN: Computers, Informatics, Nursing 35(4): 201-211. | 1.0 Clients | Mixed-Methods | Isolation | 1. To identify key constructs that may serve as barriers and facilitators 6. To frame an evaluation |
| Newman, L., et al. (2016). "Service providers' experiences of using a telehealth network 12 months after digitisation of a large Australian rural mental health service." International Journal of Medical Informatics **94**: 8-20. | 2.0 Healthcare Providers | Qualitative | Isolation | 9. To specify outcomes |
| Pelletier, A. C., et al. (2011). "Implementing a web-based home monitoring system within an academic health care network: Barriers and facilitators to innovation diffusion." Journal of Diabetes Science and Technology **5**(1): 32-38. | 1.0 Clients  2.0 Healthcare Providers | Mixed-Methods | RE-AIM | 9. To specify outcomes |
| Williamson, A., et al. (2021). "Uptake of and Engagement With an Online Sexual Health Intervention (HOPE Intervention) Among African American Young Adults: Mixed Methods Study." Journal of Medical Internet Research 23(7): e22203. | 1.0 Clients | Mixed-Methods | Isolation | 1. To identify key constructs that may serve as barriers and facilitators 7. To inform data analysis 8. To guide the selection of implementation strategies |
| Ljubicic, V., et al. (2020). "Drivers of intentions to use healthcare information systems among health and care professionals." Health Informatics Journal 26(1): 56-71. | 2.0 Healthcare Providers | Quantitative | Technology acceptance model  Technology acceptance model 2  Unified theory of acceptance and use of technology (UTAUT) | 2. To inform data collection  9. To specify outcomes |
| Wang, S. L. and H. I. Lin (2019). "Integrating TTF and IDT to evaluate user intention of big data analytics in mobile cloud healthcare system." Behaviour & Information Technology **38**(9): 974-985. | 1.0 Clients | Quantitative | Task Technology Fit (TTF) Theory | 2. To inform data collection 12. To specify hypothesized relationships between constructs |
