# Supplementary material for: Mapping Theories, Models, and Frameworks to Evaluate Digital Health Interventions: Scoping Review
Source: J Med Internet Res. 2024 Feb 5;26:e51098. doi: 10.2196/51098 (PMC10877497; doi:10.2196/51098)
Supplement: Multimedia Appendix 7 [file jmir_v26i1e51098_app7.docx]

**Multimedia Appendix 7.** Constructs of the most prevailing TMFs

The constructs and sub-constructs of the prevailing TMFs are presented in the Table below, along with their description. CFIR [1] comprises five categories of determinants influencing implementation outcomes: interventions (DHIs) characteristics, outer and inner settings (i.e. contextual determinants), individual characteristics and implementation process. The RE-AIM [2] has been developed to evaluate the following implementation outcomes: reach, efficacy, adoption, implementation, and maintenance. TAM [3], as well as its extension to TAM2 [4] and TAM3 [5] aimed to predict and explain the individual acceptance and use of technologies in end-users. The core hypothesized relationships of the variables of these models are: intention to use technology is predicted by 2 factors: 1) perceived ease of use, 2) perceived usefulness. Those 2 factors being predicted by another set of variables in TAM2 and TAM3 (such as social influence and cognitive processes). The DOI [6] suggests that the individual adopter perceptions of five attributes of innovations (independent variable) predict the rate of adoption of an innovation (dependent variable), including but not limited to DHIs: 1) perceived attributes of innovations, 2) type of innovation-decision, 3) nature of communication channels diffusing the innovation at various stages in the innovation-decision process (e.g., mass media vs interpersonal), 4) nature of the social system in which the innovation is diffusing (e.g., its norm), 5) extent of change agents’ promotion efforts in diffusing the innovation. Finally, the NPT [7] allows the identification and explanation of 4 key mechanisms promoting and inhibiting the implementation, embedding, and integration of a variety of interventions including DHIs [8]: 1) coherence building, 2) cognitive participation, 3) collective action, 4) reflexive monitoring.

Constructs of the most prevailing TMFs

| **Description** | **Constructs**  **S*ub-constructs in italic*** |
| --- | --- |
| **Consolidated Framework for Implementation Research (CFIR)** | |
| A meta-theoretical framework that specifies a list of constructs within general domains that are believed to influence (positively or negatively, as specified) implementation. | 1) Intervention characteristics  *Intervention source, evidence strength and quality, relative advantage, adaptability, trialability, complexity, design quality and packaging, cost*  2) Outer setting  *Patient needs and resources, cosmopolitanism, peer pressure, external & incentives*  3) Inner setting  *Structural characteristics, networks and communications, culture, implementation climate, readiness for implementation*  4) Characteristics of the individuals  *Knowledge & beliefs about the intervention, self-efficacy, individual stage of change, individual identification with organization, other personal attributes*  5) Process of implementation  *Planning, engaging, executing, reflecting and evaluating* |
| **Reach, Effectiveness, Adoption, Implementation, and Maintenance (RE-AIM)** | |
| Planning and evaluation framework that focuses on issues, dimensions, and steps in the design, dissemination, and implementation process that can either facilitate or impede success in achieving broad and equitable population-based impact. | 1) Reach  2) Efficacy  3) Adoption  4) Implementation  5) Maintenance |
| **Technology Acceptance Model (TAM)** | |
| An information systems theory used to predict and explain the individual acceptance (or adoption) and use of technologies in end-users. Three versions have been developed with the most recent reported here. | **TAM 3**  1) Perceived ease of use  *Computer self-efficacy, perception of external control, computer anxiety, computer playfulness, perceived enjoyment, objective usability*  2) Perceived usefulness  *Social influence processes:* *subjective norm, voluntariness, experience; Cognitive processes: image, job relevance, output quality, result demonstrability*. |
| **Unified Theory on Acceptance and Use of Technology (UTAUT)** | |
| To explain user intentions to use an information system and subsequent usage behaviour by exploring factors that determine behavioural intention. | 1) Performance Expectancy  *Perceived usefulness, extrinsic motivation, job-fit, relative advantage, outcome expectations*  2) Effort Expectancy  *Perceived ease of use, complexity, ease of use*  3) Social Influence  *Subjective norm, social factors, image*  4) Facilitating Condition  *Perceived behavioral control, facilitating conditions, compatibility* |
| **Normalization Process Theory (NPT)** | |
| NPT provides a set of conceptual tools that support understanding and evaluation of the adoption, implementation, and sustainment of socio-technical and organizational innovations. NPT has been widely used to explain how and why digital health can (or can’t) be integrated into existing working relationships and organizational structures. | 1) Coherence Building  *Differentiation, individual specification, communal specification, internalization, meaning*  2) Cognitive participation  *Initiation, legitimation, enrolment, activation, commitment*  3) Collective action  *Interactional workability, relational integration, contextual integration, skill-set workability, effort*  4) Reflexive monitoring  *Systematization, individual appraisal, communal appraisal, reconfiguration, comprehension* |
| **Diffusion of Innovation (DOI) Theory** | |
| A change model suggesting that the individual receiver’s perceptions (adopters) of five attributes of innovations (independant variable) predict an innovation’s rate of adoption (dependant variable). Five levels or categories of adopters are presented in the model. | 1) Perceived attributes of innovations  *Relative advantage, compatibility, complexity, trialability, observability*  2) Type of innovation-decision *(optional, collective, authority)*  3) Nature of communication channels diffusing the innovation at various stages in the innovation-decision process (e.g., mass media vs interpersonal)  4) Nature of the social system in which the innovation is diffusing (e.g., its norm)  5) Extent of change agents’ promotion efforts in diffusing the innovation  Adopter Categories: Innovators, Early adopters, Early Majority, Late majority, Laggards |

**References**

1. Damschroder LJ, Aron DC, Keith RE, Kirsh SR, Alexander JA, Lowery JC. Fostering implementation of health services research findings into practice: a consolidated framework for advancing implementation science. Implement Sci 2009 Aug 7;4:50. doi: 10.1186/1748-5908-4-50

2. Glasgow RE, Vogt TM, Boles SM. Evaluating the public health impact of health promotion interventions: the RE-AIM framework. Am J Public Health 1999 Sep;89(9):1322–1327. PMID:10474547

3. Davis FD. Perceived usefulness, perceived ease of use, and user acceptance of information technology | MIS Quarterly. 1989;13(3):319–339.

4. Venkatesh V, Davis FD. A Theoretical Extension of the Technology Acceptance Model: Four Longitudinal Field Studies. Manag Sci INFORMS; 2000 Feb;46(2):186–204. doi: 10.1287/mnsc.46.2.186.11926

5. Venkatesh V, Bala H. Technology Acceptance Model 3 and a Research Agenda on Interventions. Decis Sci 2008;39(2):273–315. doi: 10.1111/j.1540-5915.2008.00192.x

6. Rogers EM. Diffusion of Innovations. Fourth Edition. New York, US: Free Press; 1995.

7. May C, Finch T. Implementing, Embedding, and Integrating Practices: An Outline of Normalization Process Theory. Sociology SAGE Publications Ltd; 2009 Jun 1;43(3):535–554. doi: 10.1177/0038038509103208

8. May CR, Cummings A, Girling M, Bracher M, Mair FS, May CM, Murray E, Myall M, Rapley T, Finch T. Using Normalization Process Theory in feasibility studies and process evaluations of complex healthcare interventions: a systematic review. Implement Sci 2018 Jun 7;13(1):80. doi: 10.1186/s13012-018-0758-1
