# Supplementary material for: Mapping Theories, Models, and Frameworks to Evaluate Digital Health Interventions: Scoping Review
Source: J Med Internet Res. 2024 Feb 5;26:e51098. doi: 10.2196/51098 (PMC10877497; doi:10.2196/51098)
Supplement: Multimedia Appendix 8 [file jmir_v26i1e51098_app8.docx]

**Multimedia Appendix 8.** Use cases supported by scientific literature – Application of CFIR

| **Use case 1: Using the Consolidated Framework for Implementation Research to identify barriers and facilitators to implementation, informing implementation outcomes**  **Use case 1.1.** Hadjistavropoulos et al. [1] conducted a process evaluation to identify barriers and facilitators that influenced the implementation of an internet-delivered cognitive behavior therapy (ICBT) across seven community mental health clinics. The ICBT targeted patients suffering from anxiety and depression and it was implemented for an average of two-years. The ICBT course includes five online core lessons (text-based with visual images) delivered over 8 weeks. In the meantime, patients could contact therapists by secure email for 8 weeks and they got email responses from therapists once a week. If needed, the therapists spent 15-20 minutes per week with patients (on phone calls).  Authors utilized CFIR to identify what factors impacted implementation and those that could impact future uptake of ICBT and its sustainability. In doing so, they used a survey administered to managers and therapists (survey available in the paper). The findings showed that ICBT characteristics (e.g., relative advantage, quality of the program, evidence underlying the intervention) and implementation process facilitated the endeavor while inner setting components (e.g., limited resources, face-to-face care as a priority, readiness for implementation, implementation climate) were identified as the main barriers impacting implementation. Authors reported potential strategies that can be deployed to improve implementation efforts, such as personalizing the ICBT intervention, better integration of ICBT in therapist’s workflow, and reorganizing the model of delivering the intervention (e.g., involve mental primary care clinics).  **Use case 1.2.** Robins et al. [2] aimed to identify barriers and facilitators (determinants) to the implementation of an Electronic Communications and Home Blood Pressure Monitoring which integrated a pharmacist intensifying the blood pressure management strategies through web-messaging with patients. The CFIR was used in combination with the Chronic Care Model to identify what was influencing implementation in community settings so that adaptations to the DHI could be made. The assessment of determinants was gathered through interviews among physicians, medical assistants, licensed practice nurses, pharmacists, and patients. Barriers of implementation included the difficulty of integrating an unfamiliar pharmacist into the healthcare team (when there is no pharmacist in the “regular” team), concerns about the functioning of the collaborative practice (especially between physicians and pharmacists), lack of implementation readiness and implementation climate, limited resources (technological, financial and human), clinicians’ concerns to using the blood pressure management protocol. The sustainability of such an intervention was raised as a concern. Facilitators of implementation included the perceived advantage of the DHI in improving quality of care, empowering patients, offering additional support to patients, saving staff time, and collaborative care benefits. This highlighted the need to adapt the DHIs by addressing those barriers, for example, by looking for additional technological options for ensuring secure and synchronous communication between patient and pharmacist when the electronic health record is not available. Mechanisms to support sustainability can target incentives that value and reward team-based care models such as pay-for-performance initiative. |
| --- |

DHI(s): digital health intervention(s), CFIR: Consolidated Framework for Implementation Research; ICBT: internet-delivered cognitive behavior therapy

**References**

1. Hadjistavropoulos HD, Nugent MM, Dirkse D, Pugh N. Implementation of internet-delivered cognitive behavior therapy within community mental health clinics: a process evaluation using the consolidated framework for implementation research. BMC Psychiatry 2017 Sep 12;17(1):331. PMID:28899365

2. Robins LS, Jackson JE, Green BB, Korngiebel D, Force RW, Baldwin L-M. Barriers and facilitators to evidence-based blood pressure control in community practice. J Am Board Fam Med JABFM 2013;26(5):539–557. PMID:24004706
