# Supplementary material for: Mapping Theories, Models, and Frameworks to Evaluate Digital Health Interventions: Scoping Review
Source: J Med Internet Res. 2024 Feb 5;26:e51098. doi: 10.2196/51098 (PMC10877497; doi:10.2196/51098)
Supplement: Multimedia Appendix 9 [file jmir_v26i1e51098_app9.docx]

**Multimedia Appendix 9.** Use cases supported by scientific literature – Application of RE-AIM

| **Use case 1: Using the RE-AIM Framework to understand outcomes**  **Use case 1.1.** The RE-AIM Framework was predominantly used to inform evaluation activities (i.e., data collection and data analysis). Spaulding et al. [1] used the RE-AIM to qualitatively explore the impact of a DHI that collected electronic patient self‐reported measures (ePSRM) in a consultation-liaison psychiatry practice. Interviews were conducted among physicians, nurses, and front desk staff. The application of the RE-AIM surfaced key insights including the “reach” dimension in which the intervention was perceived as an issue for patients with cognitive or language impairments. The ePSRM tool was seen as efficient and saved time, led to more conversational sessions with patients and was patient-centered (effectiveness). One important factor of implementation was the availability of a technical support person (implementation). These insights supported areas of improvement that can address the identified barriers and that can be applied to any ePSRM implementation study, such as providing support for specific patient population in completing the ePSRM.  **Use case 1.2.** Koot et al. [2] conducted a quantitative evaluation of a mobile lifestyle management program for people with type 2 diabetes mellitus as an add-on to standard care aimed to assess the potential effectiveness and feasibility of the intervention. This program encompassed two components: online lessons and self-reporting, that were offered on two different online platforms. The RE-AIM was used to frame the evaluation, guide implementation planning, and inform data collection and analysis. Specifically, the findings showed statistically significant improvements for HbA1c (–1.3 percentage points, P<.001) that were greater among those who logged their weight more often (P=.007)(effectiveness outcome). Engagement in the intervention was higher in the first week and decreased over time (implementation dimension). 13.2 % of all patients approached for the study consented to participate (reach dimension). The authors did recommend to evaluate the effectiveness of this intervention (through randomized trial) based on promising results on health outcomes. However, those results can inform future evaluation in, for example, proactively targeting the population to whom referring the intervention (i.e improving “reach”), and identifying strategies to improve engagement over time such as making available the online lessons on the same platform than the self-reporting component of the intervention. One potential benefit of using this framework would be to collect data across RE-AIM dimensions in order to triangulate outcomes to understand the reason(s) for the observed effect. |
| --- |

DHI: digital health intervention; ePSRM: electronic patient self‐reported measures () RE-AIM: Reach, Effectiveness, Adoption, Implementation, and Maintenance; HbA1c: hemoglobin A1c

**References**

1. Spaulding A, Nordan L, Blanchfield L, Asiedu GB, Saltivan J, Pecenka S, Uitti R, Naessens J, Niazi S. Qualitative study of implementation of patient self-reported measures in a consultation-liaison psychiatry practice. J Eval Clin Pract 2019 Jun;25(3):482–490. PMID:31037796

2. Koot D, Goh PSC, Lim RSM, Tian Y, Yau TY, Tan NC, Finkelstein EA. A Mobile Lifestyle Management Program (GlycoLeap) for People With Type 2 Diabetes: Single-Arm Feasibility Study. JMIR MHealth UHealth 2019 May 24;7(5):e12965. PMID:31127720
